# Design, Catalyst-Free Synthesis of New Novel α-Trifluoromethylated Tertiary Alcohols Bearing Coumarins as Potential Antifungal Agents

**DOI:** 10.3390/molecules28010260

**Published:** 2022-12-28

**Authors:** Shengfei Jiang, Guoyu Yang, Lijun Shi, Liangxin Fan, Zhenliang Pan, Caixia Wang, Xiaodan Chang, Bingyi Zhou, Meng Xu, Lulu Wu, Cuilian Xu

**Affiliations:** 1 College of Sciences, Henan Agricultural University, Zhengzhou 450002, China; 2 Department of Information, The First Affiliated Hospital, Zhengzhou University, Zhengzhou 450052, China

**Keywords:** α-trifluoromethylated tertiary alcohol, coumarin, catalyst-free, antifungal activity

## Abstract

A new method for the synthesis of α-trifluoromethylated tertiary alcohols bearing coumarins is described. The reaction of 3-(trifluoroacetyl)coumarin and pyrrole provided the target compounds with high yields under catalyst-free, mild conditions. The crystal structure of compound **3fa** was investigated by X-ray diffraction analysis. The biological activities, such as in vitro antifungal activity of the α-trifluoromethylated tertiary alcohols against Fusarium graminearum, Fusarium oxysporum, Fusarium moniliforme, Rhizoctonia solani Kuhn, and Phytophthora parasitica var nicotianae, were investigated. The bioassay results indicated that compounds **3ad**, **3gd**, and **3hd** showed broad-spectrum antifungal activity in vitro. Compound **3cd** exhibited excellent fungicidal activity against Rhizoctonia solani Kuhn, with an EC_50_ value of 10.9 μg/mL, which was comparable to that of commercial fungicidal triadimefon (EC_50_ = 6.1 μg/mL). Furthermore, molecular docking study suggested that **3cd** had high binding affinities with 1W9U, like argifin.

## 1. Introduction

**Figure 1:**
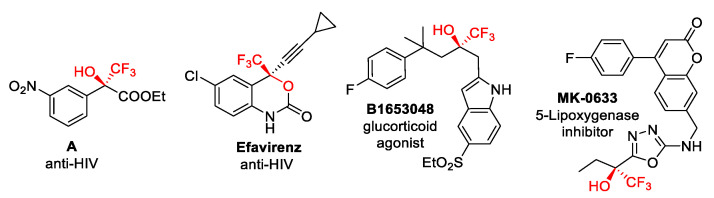
Examples of pharmaceuticals containing α-trifluoromethylated tertiary alcohol motifs.

Trifluoromethylated compounds, due to their unique properties, are of utmost interest for a wide cross-section of chemists [1,2,3,4,5]. Among them, α-trifluoromethylated tertiary alcohol motifs are found in various pharmaceuticals (Figure 1) [6,7,8,9,10,11,12,13]. For example, α-trifluoromethylated tertiary alcohol **A** and efavirenz are HIV reverse transcriptase inhibitors, B1653048 and MK-0633, respectively, are glucorticoid agonist and 5-lipoxygenase inhibitor.

**Figure 2:**
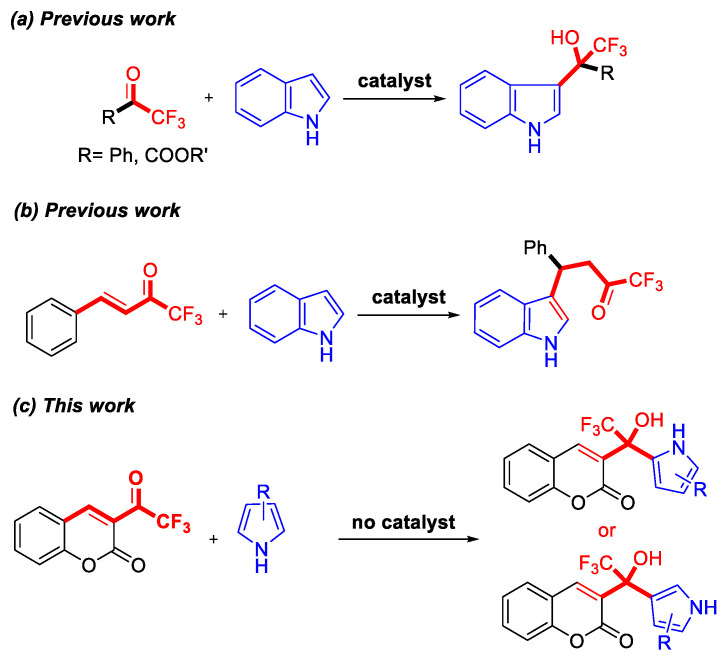
Friedel–Crafts alkylation of indole or pyrrole and trifluoromethylketone or α,β-unsaturated trifluoromethyl ketone.

Therefore, several examples of the synthesis of trifluoromethylated tertiary alcohols have been described [14,15,16,17,18]. From all the methodologies described, the Friedel−Crafts alkylation reaction with trifluoromethyl ketones is one of the most straightforward approaches for the synthesis of this kind of tertiary alcohols [19,20,21,22]. Among these reported examples, most of them use trifluoromethyl ketones as acceptors in addition reactions (Figure 2a) [23,24,25]. Interestingly, we noted that when α,β-unsaturated trifluoromethyl ketones were used as acceptors, the adducts are 1,4-addition rather than 1,2-addition (Figure 2b) [26,27,28,29]. As a part of our continuing work in the functionalization of coumarins [30,31,32,33,34], coumarin-bearing α,β-unsaturated trifluoromethyl ketones were selected as electrophiles instead of trifluoromethyl ketones to obtain trifluoromethylated tertiary alcohols (Figure 2c).

Coumarin is an important heterocyclic skeleton frequently found in numerous natural products, pharmaceutical molecules, fluorescent probes, and materials [35,36,37,38,39,40,41,42]. The combination of some privileged structures, a benzopyrone ring, a trifluoromethyl moiety, and a pyrrole ring for the synthesis of quaternary carbon organic molecules could be of significant importance, especially for new drugs and materials.

In this paper, we describe the first synthetic method for the preparation of α-trifluoromethylated tertiary alcohols bearing coumarins. Our approach is based on the two-component reaction of 3-(trifluoroacetyl)coumarin and pyrrole, and this method required neither catalyst/additive nor special conditions. In addition, the in vitro antifungal activity of the title compounds was also studied.

## 2. Results and Discussion

We readily prepared 3-(trifluoroacetyl)coumarin **1a** in two steps and directly used it as an electrophile for the Friedel–Crafts alkylation of 2-methylpyrrole **2a**. During the process of exploring reaction conditions, we were initially pleased to find that the reaction proceeded smoothly in CH_2_Cl_2_ at room temperature to provide the desired product 3aa with 46% yield in the presence of 5 mol % of AlCl_3_ (Table 1, entry 1). The success indicated that the reaction could be promoted by Lewis acid. Subsequently, a series of Lewis acids were screened, and the reactions could proceed (Table 1, entries 2–6). However, unfortunately, only low yields of tertiary alcohol product were formed. To our surprise, 97% yield was observed by performing the reaction in the absence of catalyst (Table 1, entry 7). Finally, the reaction media were screened. Solvent evaluation indicated that the solvents have a remarkable influence on the yields (Table 1, entries 7–15). The yield sharply decreased when chloroform, ethyl acetate, acetonitrile, toluene, and tetrahydrofuran were used as the solvents (Table 1, entries 8–12). Methylene chloride was the best solvent, giving the highest yield of 97%. Unfortunately, the yield sharply decreased when reducing the reaction temperature to 0 ℃ (Table 1, entry 16).

Having established the preferred reaction conditions, we next examined the substrate scope of the Friedel–Crafts reaction (Table 2). A range of 3-(trifluoroacetyl)coumarins with different substituents at the 6-, 7-, or 8- positions proceeded smoothly to afford the corresponding α-trifluoromethyl tertiary alcohols **3aa**–**ia** with excellent yields. Subsequently, pyrroles **2b**–**f** with different groups were also tested for this synthesis. They proceeded well, furnishing the desired products **3ab**–**af** with moderate to excellent yields. The position and electronic property of substituents had a remarkable influence on the yields. The pyrrole ring with electron-donating substituents at the 2-, 3-, or 4- positions proceeded smoothly to afford the corresponding α-trifluoromethyl tertiary alcohols with excellent yields, while the presence of electron-donating substituents in the 2- and 5- positions had a detrimental effect on the yield, due to the activity of the 2-position pyrrole higher than the 3-positon. It was gratifying that excellent yields could be achieved by adding Sc(OTf)_3_ and increasing the reaction temperature. Unfortunately, the pyrrole ring with an electron-deficient group was an infeasible substrate, presumably because electron withdrawal affected the activity of the pyrrole. Notably, when the pyrrole and N-methylpyrrole were employed, a comparable yield was obtained.

**Scheme 1:**
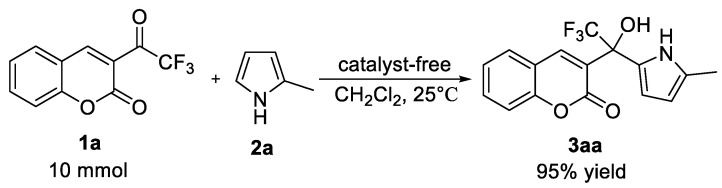
Scaled-up experiment of the one-pot reaction.

To test the feasibility of potential large-scale application of the process, the reaction of 3-(trifluoroacetyl)coumarin **1a** with 2-methylpyrrole **2a** was carried out at the 10 mmol scale under the standard conditions (Scheme 1). The reaction furnished a 95% yield of the product **3aa,** which is quite comparable to the yield obtained in the small scale reaction (97% yield).

**Figure 3:**
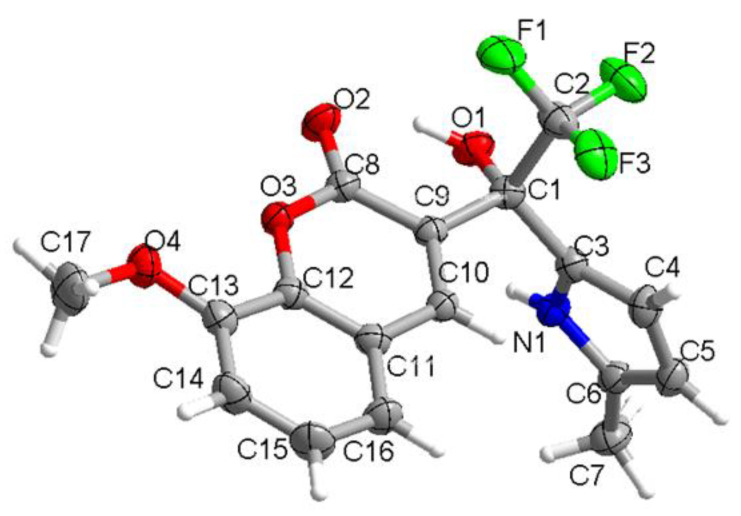
X-ray crystal structure of compound **3fa** (CCDC 2167896).

To determine the structures of the products, a single crystal of compound **3fa** was obtained (Figure 3). The structures of other products can therefore be determined by analogy. 

The in vitro antifungal activity of target compounds **3aa-3hd** against six representative phytopathogens is summarized in Table 3. Triadimefon was used as a positive control at a concentration of 10 μg/mL. In general, the title compounds exhibited a certain degree of fungicidal activity at a concentration of 500 μg/mL. Among them, compounds **3ad**, **3bd,** and **3cd** showed over 90% inhibition against F. graminearum (from corn). Compounds **3ad**, **3bd**, **3gd,** and **3hd** showed over 80% inhibition against F. oxysporum. Compounds **3ad** and **3bd** showed over 80% inhibition against F. graminearum (from wheat). In particular, **3ad**, **3gd,** and **3hd** exhibited excellent activity (>98%) against R. solani Kuhn. Compounds **3ac**, **3ad**, **3bd**, **3gd,** and **3hd** exhibited higher antifungal activity (> 80%) against P. parasitica var nicotianae. Furthermore, it was also worth noting that **3ad**, **3gd,** and **3hd** exhibited broad-spectrum antifungal activity and could be considered as new fungicidal leads for further optimization.

The in vitro fungicidal activity of the title compounds against six phytopathogens showed that the substituents have a great impact on the activity. Overall, the fungicidal activity of 3-ethyl-2,4-dimethyl-1H-pyrrole derivatives (**3ad**, **3bd**, **3cd**, **3gd,** and **3hd**) showed generally higher inhibition against the tested fungi than other substituted pyrrole derivatives.

Several compounds with higher preliminary antifungal activities at a concentration of 500 μg/mL were selected for determination of the median effective concentration (EC_50_) values. As shown in Table 4, four compounds displayed good fungicidal activity against R. solani Kuhn. Among them, compound **3cd** was the most potent and had the EC_50_ values of 10.9 μg/mL. The results indicated that most of the coumarin derivatives containing α-trifluoromethylated tertiary alcohols exhibited good fungicidal activity.

**Figure 4:**
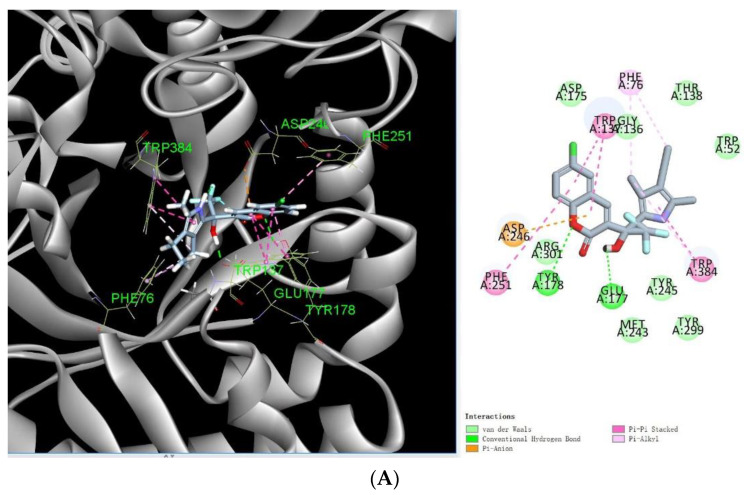

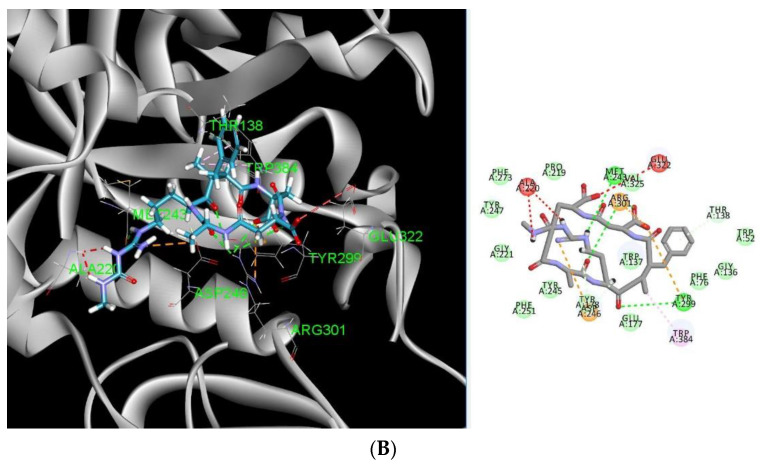
Docking modes of **3cd** (**A**) and inhibitor argifin (**B**) with 1W9U.

Studies have reported that coumarin compounds can bind with the groove of chitinese and revealed better fungal inhibitory activity [43]. The molecular docking of the compound **3cd** with 1W9U was conducted to explore the probable interaction with chitinese, and argifin was used as the standard of comparison. As can be seen from Figure 4, compared with argifin, the coumarin derivative **3cd** shows some similar aminoacid residues interacting with the receptor. For instance, TYR 245 formed a strong hydrogen bond with the fluorine that emerged as a hydrogen bond donor in the title compound. Then, another hydrogen bond appeared between the hydrogen in the pyrrole ring and ASP 246. It is worth noting that the target compound bonds to GLU 177 by a strong hydrogen bond, while argifin bonds to GLU 177 by the Van der Waals force. Although **3cd** does not bind to GLU 178 by hydrogen bond as argifin does, it also binds to GLU 178 by Pi-Pi stacked interaction. Concurrently, the title compound **3cd** connected with the TRP52, PHE76, GLY136, TRP137, PHE251, and TRP384 residues, which also formed weak interactions with the chitinese inhibitor argifin. As can be seen from the above results, our synthesized coumarin compounds are expected to be novel chitinese inhibitors, and the relative work is on the way.

## 3. Materials and Methods

### 3.1. Chemicals and Instruments

All chemicals, except 3-(trifluoroacetyl)coumarin 1, which was synthesized according our reported procedure, were purchased from commercial sources and used without further purification. ^1^H NMR, ^19^F NMR, and ^13^C NMR spectra were obtained using a Bruker DPX-400 spectrometer (Brucker Technologies Co., Karlsruhe, Germany) in CDCl_3_ or DMSO solution with TMS as an internal standard. HR-MS(APCI) spectra were performed using a Waters Q-Tof MicroTM instrument (Thermo Fisher Scientific, Waltham, Massachusetts, USA), and X-rays were measured at 293 K on a Rigaku RAXIAS-IV type diffractometer (Brucker Technologies Co., Karlsruhe, Germany). Most reaction yields, except compound **3fa**, were not optimized. CCDC 2,167,896 contains the supplementary crystallographic data for this paper (Table S1 in the Supplement Materials). These data may be obtained free of charge via http://www.ccdc.cam.ac.uk; access on 15 November 2022.

#### 3.1.1. General Procedure for the Preparation of Compounds **3aa**–**3af**

A mixture of 3-(trifluoroacetyl)coumarin (1.0 mmol) and pyrrole (1.0 mmol) was stirred in methylene chloride (10 mL) at room temperature. After completion of the reaction, the mixture was concentrated under vacuum to yield the crude product, which was further purified by column chromatography.

To a mixture of 3-(trifluoroacetyl)coumarin (1.0 mmol) and pyrrole (1.0 mmol) in methylene chloride (10 mL) was added Sc(OTf)_3_ (5%), and the resulting mixture was heated under reflux. After completion of the reaction, the mixture was concentrated under vacuum to yield the crude product, which was further purified by column chromatography.

#### 3.1.2. Compounds Data

3-(2,2,2-Trifluoro-1-hydroxy-1-(5-methyl-1H-pyrrol-2-yl)ethyl)-2H-chromen-2-one (**3aa**): Light Brown solid, mp: 134.5-135.6 °C. ^1^H NMR (400 MHz, CDCl_3_) δ 8.43 (s, 1H), 7.87 (s, 1H), 7.61 (t, *J* = 7.8 Hz, 1H), 7.52 (d, *J* = 7.7 Hz, 1H), 7.40–7.32 (m, 2H), 7.28 (s, 1H), 6.25 (s, 1H), 5.92 (s, 1H), 2.26 (s, 3H). ^13^C NMR (100 MHz, CDCl_3_) δ 162.47 (C=O), 153.22, 145.16, 133.21, 129.12, 128.97, 125.29, 124.49 (q, *J* = 286.6 Hz, C-CF_3_), 123.50, 122.35, 118.39, 116.60, 108.76, 106.82, δ 76.71 (q, *J* = 31.3 Hz, CF_3_), 12.96. ^19^F NMR (376 MHz, CDCl_3_) δ -78.01(CF_3_). HRMS (APCI): *m/z* calculated for C_16_H_11_F_3_NO_3_ [M-H]^−^ 322.0691, found: 322.0685.

6-Methyl-3-(2,2,2-trifluoro-1-hydroxy-1-(5-methyl-1H-pyrrol-2-yl)ethyl)-2H-chromen-2-one (**3ba**): Light yellow solid, mp: 207.9–208.7 °C. ^1^H NMR (400 MHz, DMSO-*d*_6_) δ10.46 (s, 1H), 8.29 (s, 1H), 7.70 (s, 1H), 7.48 (d, *J* = 8.5 Hz, 1H), 7.33 (d, *J* = 8.4 Hz, 1H), 7.16 (s, 1H), 6.01 (s, 1H), 5.69 (s, 1H), 2.38 (s, 3H), 2.12 (s, 3H). ^13^C NMR (101 MHz, DMSO-*d*_6_) δ 158.48(C=O), 152.02, 143.74, 134.41, 133.92, 129.46, 128.37, 125.48, 125.30 (q, *J* = 287.4 Hz, C-CF_3_), 124.53, 118.64, 115.98, 107.68, 105.78, δ 74.36 (q, *J* = 29.9 Hz, CF_3_), 20.68, 13.09. ^19^F NMR (376 MHz, DMSO-*d*_6_) δ -74.08(CF_3_). HRMS (APCI): *m/z* calculated for C_17_H_13_F_3_NO_3_ [M-H]^−^ 336.0848, found: 336.0833.

6-Chloro-3-(2,2,2-trifluoro-1-hydroxy-1-(5-methyl-1H-pyrrol-2-yl)ethyl)-2H-chromen-2-one (**3ca**): White solid, mp: 235.9-236.5 °C. ^1^H NMR (400 MHz, DMSO-*d*_6_) δ 10.43 (s, 1H), 8.45 (s, 1H), 8.13 (d, *J* = 2.5 Hz, 1H), 7.71 (dd, *J* = 8.8, 2.6 Hz, 1H), 7.48 (d, *J* = 8.9 Hz, 1H), 7.20 (s, 1H), 6.01 (s, 1H), 5.69 (s, 1H), 2.11 (s, 3H). 13C NMR (100 MHz, DMSO-*d*_6_) δ 157.47(C=O), 152.62, 142.80, 132.58, 129.50, 128.95, 128.81, 128.38, 126.01, 125.35, 125.20(q, *J* = 287.4 Hz, C-CF_3_), 120.41, 118.23, 107.69, 105.86, 74.07(q, *J* = 30.1 Hz, CF_3_), 13.09. ^19^F NMR (376 MHz, DMSO-*d*_6_) δ -73.76(CF_3_). HRMS (APCI): *m/z* calculated for C_16_H_10_ClF_3_NO_3_ [M-H]^−^ 356.0301, found: 356.0295.

6-Bromo-3-(2,2,2-trifluoro-1-hydroxy-1-(5-methyl-1H-pyrrol-2-yl)ethyl)-2H-chromen-2-one (**3da**): Cyan solid, mp: 236.5–236.9 °C. ^1^H NMR (400 MHz, DMSO-*d*_6_) δ 10.41 (s, 1H), 8.43 (s, 1H), 8.24 (d, *J* = 2.3 Hz, 1H), 7.81 (dd, *J* = 8.8, 2.4 Hz, 1H), 7.40 (d, *J* = 8.8 Hz, 1H), 7.17 (s, 1H), 6.01 (s, 1H), 5.68 (s, 1H), 2.11 (s, 3H). ^13^C NMR (100 MHz, DMSO-*d*_6_) δ 157.41(C=O), 153.01, 142.70, 135.33, 131.94, 128.36, 125.94, 125.34, 125.20 (q, *J* = 287.3 Hz, C-CF_3_), 120.90, 118.50, 116.58, 107.67, 105.84, 74.07 (q, *J* = 30.3 Hz, CF_3_), 13.09. ^19^F NMR (376 MHz, DMSO-*d*_6_) δ -73.78(CF_3_). HRMS (APCI): *m/z* calculated for C_16_H_10_BrF_3_NO_3_ [M-H]^−^ 399.9796, found: 399.9782.

7-Methoxy-3-(2,2,2-trifluoro-1-hydroxy-1-(5-methyl-1H-pyrrol-2-yl)ethyl)-2H-chromen-2-one (**3ea**): White solid, mp: 160.5-161.8 °C. ^1^H NMR (400 MHz, DMSO-*d*_6_) δ 10.45 (s, 1H), 8.27 (s, 1H), 7.82 (d, *J* = 8.6 Hz, 1H), 7.09 (s, 1H), 7.04 (d, *J* = 2.4 Hz, 1H), 6.99 (dd, *J* = 8.6, 2.4 Hz, 1H), 6.01 (s, 1H), 5.68 (s, 1H), 3.88 (s, 3H), 2.12 (s, 3H). ^13^C NMR (100 MHz, DMSO-*d*_6_) δ 163.53(C=O), 158.79, 155.83, 143.94, 130.98, 128.30, 125.65, 125.40 (q, *J* = 287.5 Hz, C-CF_3_), 120.75, 113.20, 112.48, 107.63, 105.73, 100.51, 74.31 (q, *J* = 60.1, 30.1 Hz, CF_3_), 56.52, 13.10. ^19^F NMR (376 MHz, DMSO-*d*_6_) δ -74.31(CF_3_). HRMS (APCI): *m/z* calculated for C_17_H_13_F_3_NO_4_ [M-H]^−^ 352.0797, found: 352.0783.

8-Methoxy-3-(2,2,2-trifluoro-1-hydroxy-1-(5-methyl-1H-pyrrol-2-yl)ethyl)-2H-chromen-2-one (**3fa**): Pink solid, mp: 198.1-198.6 °C. ^1^H NMR (400 MHz, DMSO-*d*_6_) δ 10.44 (s, 1H), 8.36 (s, 1H), 7.46 (dd, *J* = 7.1, 1.9 Hz, 1H), 7.40–7.29 (m, 2H), 7.15 (d, *J* = 0.9 Hz, 1H), 6.01 (s, 1H), 5.69 (s, 1H), 3.93 (s, 3H), 2.11 (s, 3H). ^13^C NMR (100 MHz, CDCl_3_) δ 162.02(C=O), 147.06, 145.28, 142.86, 128.75, 125.17, 124.47 (q, *J* = 286.7 Hz, C-CF_3_), 123.46, 122.50, 120.22, 119.05, 114.72, 108.69, 106.77, 76.68 (q, *J* = 31.3 Hz, CF_3_), 56.35, 12.93. ^19^F NMR (376 MHz, CDCl_3_) δ -78.04(CF_3_). HRMS (APCI): *m/z* calculated for C_17_H_13_F_3_NO_4_ [M-H]^−^ 352.0797, found: 352.0791.

6,8-Dichloro-3-(2,2,2-trifluoro-1-hydroxy-1-(5-methyl-1H-pyrrol-2-yl)ethyl)-2H-chromen-2-one (**3ga**): Light yellow solid, mp: 168.5-169.1 °C. ^1^H NMR (400 MHz, CDCl_3_) δ 8.45 (s, 1H), 7.76 (s, 1H), 7.63 (d, *J* = 2.3 Hz, 1H), 7.43 (d, *J* = 2.3 Hz, 1H), 6.88 (s, 1H), 6.22 (s, 1H), 5.91 (s, 1H), 2.26 (s, 3H). ^13^C NMR (100 MHz, CDCl_3_) δ 160.77(C=O), 147.55, 143.78, 132.98, 130.53, 129.39, 128.53, 126.86, 124.71, 124.24 (q, *J* = 286.7 Hz, C-CF_3_). 122.72, 122.63, 120.13, 119.98, 76.73 (q, *J* = 31.5 Hz, CF_3_), 12.92. ^19^F NMR (376 MHz, CDCl_3_) δ -77.71(CF_3_). HRMS (APCI): *m/z* calculated for C_16_H_9_Cl_2_F_3_NO_3_ [M-H]^−^ 389.9912, found: 389.9903.

6,8-Dibromo-3-(2,2,2-trifluoro-1-hydroxy-1-(5-methyl-1H-pyrrol-2-yl)ethyl)-2H-chromen-2-one (**3ha**): Cyan solid, mp: 162.1–163.2 °C. ^1^H NMR (400 MHz, CDCl_3_) δ8.47 (s, 1H), 7.92 (d, *J* = 2.2 Hz, 1H), 7.74 (s, 1H), 7.61 (d, *J* = 2.2 Hz, 1H), 6.87 (s, 1H), 6.23 (s, 1H), 5.91 (s, 1H), 2.26 (s, 3H). ^13^C NMR (100 MHz, CDCl_3_) δ 160.85(C=O), 149.06, 143.75, 138.01, 130.57, 129.38, 124.64, 124.25 (q, *J* = 287.0 Hz, C-CF_3_), 122.74, 120.53, 117.88, 111.52, 108.96, 106.95, 76.69 (q, *J* = 31.7 Hz, CF_3_), 12.95. ^19^F NMR (376 MHz, CDCl_3_) δ -77.71(CF_3_). HRMS (APCI): *m/z* calculated for C_16_H_9_Br_2_F_3_NO_3_ [M-H]^−^ 479.8881, found: 479.8868.

3-(2,2,2-Trifluoro-1-hydroxy-1-(5-methyl-1H-pyrrol-2-yl)ethyl)-2H-benzo[h]chromen-2-one (**3ia**): Tawny solid, mp: 192.0-192.9 °C. ^1^H NMR (400 MHz, CDCl_3_) δ 8.68 (s, 1H), 8.54 (s, 1H), 8.12–8.00 (m, 2H), 7.91 (d, *J* = 8.1 Hz, 1H), 7.71 (t, *J* = 7.6 Hz, 1H), 7.60 (t, *J* = 7.5 Hz, 1H), 7.51–7.40 (m, 2H), 7.26 (s, 1H), 6.39 (s, 1H), 6.01 (s, 1H), 2.28 (s, 3H). ^13^C NMR (100 MHz, CDCl_3_) δ 162.56(C=O), 153.31, 141.04, 134.78, 130.47, 129.16, 129.01, 128.97, 128.90, 126.70, 124.65 (q, *J* = 286.8 Hz, C-CF_3_), 123.60, 121.48, 121.04, 116.24, 112.91, 108.65, 107.03, 76.95 (q, *J* = 31.2 Hz, CF_3_), 12.97. ^19^F NMR (376 MHz, CDCl_3_) δ -78.07(CF_3_). HRMS (APCI): *m/z* calculated for C_20_H_13_F_3_NO_3_ [M-H]^−^ 372.0848, found: 372.0838.

3-(1-(3,5-Dimethyl-1H-pyrrol-2-yl)-2,2,2-trifluoro-1-hydroxyethyl)-2H-chromen-2-one (**3ab**): Pink solid, mp: 131.7–132.8 °C. ^1^H NMR (400 MHz, CDCl_3_) δ 8.18 (s, 1H), 7.78 (s, 1H), 7.57–7.47 (m, 2H), 7.33–7.27 (m, 2H), 7.17 (s, 1H), 6.26 (s, 1H), 5.62 (d, *J* = 3.1 Hz, 1H), 2.14 (s, 3H), 1.78 (s, 3H). ^13^C NMR (100 MHz, CDCl_3_) δ 161.74(C=O), 153.09, 142.52, 133.01, 129.11, 128.81, 127.39 (q, *J* = 286.7 Hz, C-CF_3_), 127.25, 125.31, 123.10, 118.92, 118.19, 118.01, 116.66, 110.71, 76.50 (q, *J* = 31.3 Hz, CF_3_), 12.91, 12.48. 19F NMR (376 MHz, CDCl_3_) δ -75.55(CF_3_). HRMS (APCI): *m/z* calculated for C_17_H_13_F_3_NO_3_ [M-H]^−^ 336.0848, found: 336.0833.

3-(1-(3,5-Dimethyl-1H-pyrrol-2-yl)-2,2,2-trifluoro-1-hydroxyethyl)-8-methoxy-2H-chromen-2-one (**3fb**): Orange solid, mp: 67.1-68.5 °C. ^1^H NMR (400 MHz, CDCl_3_) δ 8.24 (s, 1H), 7.83 (s, 1H), 7.33–7.26 (m, 2H), 7.14 (dd, *J* = 7.1, 5.4 Hz, 2H), 6.40 (s, 1H), 5.69 (d, *J* = 3.1 Hz, 1H), 3.98 (s, 3H), 2.23 (s, 3H), 1.86 (s, 3H). ^13^C NMR (100 MHz, CDCl_3_) δ 161.34(C=O), 147.08, 142.74, 127.28, 125.25, 124.58 (q, *J* = 286.4 Hz, C-CF_3_), 123.27, 120.33, 120.24, 118.91, 118.86, 118.05, 114.62, 110.69, 76.54 (q, *J* = 31.1 Hz, CF_3_), 56.31, 12.87, 12.50. ^19^F NMR (376 MHz, CDCl_3_) δ -75.57(CF_3_). HRMS (APCI): *m/z* calculated for C_18_H_15_F_3_NO_3_ [M-OH]^+^ 350.0999, found: 350.1008.

3-(1-(2,5-Dimethyl-1H-pyrrol-3-yl)-2,2,2-trifluoro-1-hydroxyethyl)-2H-chromen-2-one (**3ac**): Cyan solid, mp: 109.7–110.8 °C. ^1^H NMR (400 MHz, CDCl_3_) δ 7.86 (s, 1H), 7.73 (s, 1H), 7.59 (t, *J* = 8.5 Hz, 1H), 7.50 (d, *J* = 7.7 Hz, 1H), 7.40–7.28 (m, 2H), 6.59 (s, 1H), 5.90 (s, 1H), 2.21 (s, 3H), 2.16 (s, 3H). ^13^C NMR (100 MHz, CDCl_3_) δ 162.19(C=O), 153.26, 144.34, 132.76, 128.94, 125.92, 125.34 (q, *J* = 287.4 Hz, C-CF_3_), 125.22, 125.10, 124.76, 121.07, 118.59, 116.54, 114.97, 106.45, 77.99 (q, *J* = 30.0 Hz, CF_3_), 12.96, 12.80. ^19^F NMR (376 MHz, CDCl_3_) δ -76.82(CF_3_). HRMS (APCI): *m/z* calculated for C_17_H_13_F_3_NO_2_ [M-OH]^+^ 320.0893, found: 320.0917.

3-(1-(2,5-Dimethyl-1H-pyrrol-3-yl)-2,2,2-trifluoro-1-hydroxyethyl)-6-methyl-2H-chromen-2-one (**3bc**): White solid, mp: 161.2–162.0 °C. ^1^H NMR (400 MHz, CDCl_3_) δ 7.75 (s, 1H), 7.67 (s, 1H), 7.39 (dd, *J* = 8.5, 1.3 Hz, 1H), 7.29 (d, *J* = 7.1 Hz, 1H), 6.61 (s, 1H), 5.89 (s, 1H), 2.39 (s, 1H), 2.21 (s, 1H), 2.17 (s, 1H). ^13^C NMR (100 MHz, CDCl_3_) δ 162.34(C=O), 151.42, 144.19, 134.87, 133.75, 128.62, 125.84, 125.35 (q, *J* = 287.5 Hz, C-CF_3_), 125.03, 124.63, 118.35, 116.24, 115.17, 106.54, 77.96 (q, *J* = 30.0 Hz, CF_3_), 20.71, 12.99, 12.82. ^19^F NMR (376 MHz, CDCl_3_) δ -76.88(CF_3_). HRMS (APCI): *m/z* calculated for C_18_H_15_F_3_NO_3_ [M-H]^−^ 350.1004, found: 350.0989.

6-Chloro-3-(1-(2,5-dimethyl-1H-pyrrol-3-yl)-2,2,2-trifluoro-1-hydroxyethyl)-2H-chromen-2-one (**3cc**): Yellow solid, mp: 145.2–146.5 °C. ^1^H NMR (400 MHz, CDCl_3_) δ 7.87 (s, 1H), 7.66 (s, 1H), 7.52 (dd, *J* = 10.9, 2.1 Hz, 2H), 7.32 (d, *J* = 8.6 Hz, 1H), 6.39 (s, 1H), 5.87 (s, 1H), 2.20 (s, 3H), 2.15 (s, 3H). ^13^C NMR (100 MHz, CDCl_3_) δ 157.47(C=O), 152.62, 142.80, 132.58, 129.50, 128.95, 126.01, 125.35, 125.18 (q, *J* = 287.1 Hz, C-CF_3_), 120.93, 120.41, 118.23, 107.69, 105.86, 78.00 (q, *J* = 30.0 Hz, CF_3_), 12.95, 12.77. ^19^F NMR (376 MHz, CDCl_3_) δ -76.72(CF_3_). HRMS (APCI): *m/z* calculated for C_17_H_12_ClF_3_NO_3_ [M-H]^−^ 370.0458, found: 370.0449.

6-Bromo-3-(1-(2,5-dimethyl-1H-pyrrol-3-yl)-2,2,2-trifluoro-1-hydroxyethyl)-2H-chromen-2-one (**3dc**): Brown solid, mp: 168.6–170.4 °C. ^1^H NMR (400 MHz, CDCl_3_) δ 7.74 (s, 1H), 7.65 (dd, J = 6.7, 2.9 Hz, 3H), 7.24 (s, 1H), 6.32 (s, 1H), 5.85 (s, 1H), 2.19 (s, 3H), 2.15 (s, 3H). ^13^C NMR (100 MHz, CDCl_3_) δ 161.34(C=O), 152.10, 142.81, 135.42, 131.12, 126.64, 125.86, 125.16 (q, *J* = 287.7 Hz, C-CF_3_), 124.88, 120.07, 118.28, 117.68, 114.78, 106.45, 77.97 (q, *J* = 30.0 Hz, CF_3_), 13.02, 12.82. ^19^F NMR (376 MHz, CDCl_3_) δ -76.70(CF_3_). HRMS (APCI): *m/z* calculated for C_17_H_12_BrF_3_NO_3_ [M-H]^−^ 413.9953, found: 413.9936.

3-(1-(2,5-Dimethyl-1H-pyrrol-3-yl)-2,2,2-trifluoro-1-hydroxyethyl)-7-methoxy-2H-chromen-2-one (**3ec**): Yellow solid, mp: 80.1–81.7 °C. ^1^H NMR (400 MHz, CDCl_3_) δ 8.28 (s, 1H), 7.81 (s, 1H), 7.24 (m, 1H), 7.08 (s, 1H), 6.38 (s, 1H), 5.66 (s, 1H), 3.93 (s, 3H), 2.18 (s, 3H), 1.84 (s, 3H). ^13^C NMR (100 MHz, CDCl_3_) δ 161.59(C=O), 147.06, 144.21, 126.74, 125.88, 125.51, 125.49 (q, *J* = 287.5 Hz, C-CF_3_), 124.90, 124.61, 123.89, 120.06, 115.11, 114.29, 106.53, 77.89 (q, *J* = 29.9 Hz, CF_3_), 56.33, 13.00, 12.82. ^19^F NMR (376 MHz, CDCl_3_) δ -76.86(CF_3_). HRMS (APCI): *m/z* calculated for C_18_H_15_F_3_NO_4_ [M-H]^−^ 366.0953, found: 366.0941.

3-(1-(2,5-Dimethyl-1H-pyrrol-3-yl)-2,2,2-trifluoro-1-hydroxyethyl)-8-methoxy-2H-chromen-2-one (**3fc**): Cyan solid, mp: 79.8–81.1 °C. ^1^H NMR (400 MHz, CDCl_3_) δ 7.72 (s, 1H), 7.69 (s, 1H), 7.23 (d, *J* = 7.9 Hz, 1H), 7.12 (d, *J* = 8.1 Hz, 1H), 7.06 (d, *J* = 7.8 Hz, 1H), 6.55 (s, 1H), 5.78 (d, *J* = 82.7 Hz, 1H), 4.02–3.88 (m, 1H), 2.21 (s, 1H), 2.17 (s, 1H). ^13^C NMR (100 MHz, CDCl_3_) δ 161.59(C=O), 147.06, 144.21, 125.88, 125.51, 125.49 (q, *J* = 287.5 Hz, C-CF_3_), 124.90, 124.61, 123.89, 120.06, 119.27, 115.11, 114.29, 106.53, 77.89 (q, *J* = 29.9 Hz, CF_3_), 56.33, 13.00, 12.82. ^19^F NMR (376 MHz, CDCl_3_) δ -76.86(CF_3_). HRMS (APCI): *m/z* calculated for C_18_H_15_F_3_NO_4_ [M-H]^−^ 366.0953, found: 366.0941.

6,8-Dichloro-3-(1-(2,5-dimethyl-1H-pyrrol-3-yl)-2,2,2-trifluoro-1-hydroxyethyl)-2H-chromen-2-one (**3gc**): Tawny solid, mp: 119.1–120.3 °C. ^1^H NMR (400 MHz, CDCl_3_) δ 7.81 (s, 1H), 7.68-7.60 (m, 2H), 7.42 (d, *J* = 2.3 Hz, 1H), 6.16 (s, 1H), 5.86 (s, 1H), 2.21 (s, 3H), 2.17 (s, 3H). ^13^C NMR (100 MHz, CDCl_3_), δ 160.74(C=O), 150.72, 143.67, 133.55, 130.24, 127.58, 126.66, 125.91, 125.05 (q, *J* = 287.4 Hz, C-CF_3_), 124.67, 122.56, 120.36, 115.05, 108.26, 77.96 (q, *J* = 30.2 Hz, CF_3_), 12.98, 12.78. ^19^F NMR (376 MHz, CDCl_3_) δ -76.73(CF_3_). HRMS (APCI): *m/z* calculated for C_17_H_11_Cl_2_F_3_NO_3_ [M-H]^−^ 404.0068, found: 404.0052.

6,8-Dibromo-3-(1-(2,5-dimethyl-1H-pyrrol-3-yl)-2,2,2-trifluoro-1-hydroxyethyl)-2H-chromen-2-one (**3hc**): Yellow solid, mp: 172.6–174.3 °C. ^1^H NMR (400 MHz, CDCl_3_) δ 7.92 (d, *J* = 2.1 Hz, 1H), 7.80 (s, 1H), 7.63–7.61 (m, 2H), 6.19 (s, 1H), 5.86 (s, 1H), 2.21 (s, 3H), 2.16 (s, 3H). ^13^C NMR (100 MHz, CDCl_3_), δ 160.40(C=O), 149.16, 142.67, 138.06, 129.36, 127.48, 125.53 (q, *J* = 287.5 Hz, C-CF_3_), 125.00, 120.79, 120.16, 117.60, 114.46, 111.06, 106.34, 77.91 (q, *J* = 30.1 Hz, CF_3_), 13.00, 12.80. ^19^F NMR (376 MHz, CDCl_3_) δ -77.71 (CF_3_). HRMS (APCI): *m/z* calculated for C_17_H_11_Br_2_F_3_NO_2_ [M-OH]^+^ 477.9083, found: 477.9106.

3-(1-(2,5-Dimethyl-1H-pyrrol-3-yl)-2,2,2-trifluoro-1-hydroxyethyl)-2H-benzo[h]chromen-2-one (**3ic**): Khaki solid, mp: 168.6–170.4 °C. ^1^H NMR (400 MHz, DMSO-*d*_6_) δ 10.69 (s, 1H), 9.38 (s, 1H), 8.67 (d, *J* = 8.5 Hz, 1H), 8.49 (d, *J* = 9.1 Hz, 1H), 8.35 (d, *J* = 8.1 Hz, 1H), 8.05 (t, *J* = 7.7 Hz, 1H), 7.97–7.83 (m, 2H), 6.16 (s, 1H), 2.47 (s, 3H), 2.30 (s, 3H). ^13^C NMR (100 MHz, CDCl_3_) δ 162.13(C=O), 153.25, 139.80, 134.20, 133.03, 130.48, 129.16, 128.63, 128.29, 127.58 (q, *J* = 294.1 Hz, C-CF_3_), 126.51, 125.94, 124.26, 121.52, 116.44, 115.18, 112.99, 106.55, 78.16 (q, *J* = 30.2 Hz, CF_3_), 13.11, 12.90. ^19^F NMR (376 MHz, CDCl_3_) δ -76.70(CF_3_). HRMS (APCI): *m/z* calculated for C_21_H_15_F_3_NO_3_ [M-H]^−^ 386.1004, found: 386.0996.

3-(1-(4-Ethyl-3,5-dimethyl-1H-pyrrol-2-yl)-2,2,2-trifluoro-1-hydroxyethyl)-2H-chromen-2-one (**3ad**): Yellow solid, mp: 162.9–163.5 °C. ^1^H NMR (400 MHz, CDCl_3_) δ 8.10 (s, 1H), 7.85 (s, 1H), 7.65–7.56 (m, 2H), 7.38 (dd, *J* = 14.8, 7.6 Hz, 2H), 6.35 (s, 1H), 2.34 (q, *J* = 7.4 Hz, 2H), 2.19 (s, 3H), 1.83 (s, 3H), 1.02 (t, *J* = 7.5 Hz, 3H). ^13^C NMR (100 MHz, CDCl_3_) δ 161.74(C=O), 153.14, 142.61, 132.95, 129.10, 126.09, 125.27, 124.72 (q, *J* = 272.1 Hz, C-CF_3_), 123.39, 123.26, 122.46, 118.28, 118.08, 116.68, 76.68 (q, *J* = 30.8 Hz, CF_3_), 17.51, 15.56, 11.19, 10.08. ^19^F NMR (376 MHz, CDCl_3_) δ -75.42(CF_3_). HRMS (APCI): *m/z* calculated for C_19_H_17_F_3_NO_3_ [M-H]^−^ 364.1161, found: 364.1150.

3-(1-(4-Ethyl-3,5-dimethyl-1H-pyrrol-2-yl)-2,2,2-trifluoro-1-hydroxyethyl)-6-methyl-2H-chromen-2-one (**3bd**): Yellow solid, mp: 160.8–161.9 °C. ^1^H NMR (400 MHz, CDCl_3_) δ 8.05 (s, 1H), 7.75 (s, 1H), 7.37 (m, 2H), 7.23 (d, *J* = 4.1 Hz, 1H), 6.39 (s, 1H), 2.42 (s, 3H), 2.36 (q, *J* = 7.5 Hz, 2H), 2.15 (s, 3H), 1.78 (s, 3H), 0.98 (t, *J* = 7.5 Hz, 3H). ^13^C NMR (100 MHz, CDCl_3_) δ 162.01(C=O), 151.29, 142.67, 135.15, 134.03, 128.82, 124.68 (q, *J* = 286.8 Hz, C-CF_3_), 123.18, 123.13, 122.43, 118.17, 118.04, 116.62, 116.35, 76.69 (q, *J* = 30.7 Hz, CF_3_), 20.75, 17.51, 15.57, 11.19, 10.06. ^19^F NMR (376 MHz, CDCl_3_) δ -75.46(CF_3_). HRMS (APCI): *m/z* calculated for C_20_H_19_F_3_NO_3_ [M-H]^−^ 378.1317, found: 378.1299.

6-Chloro-3-(1-(4-ethyl-3,5-dimethyl-1H-pyrrol-2-yl)-2,2,2-trifluoro-1-hydroxyethyl)-2H-chromen-2-one (**3cd**): Light yellow solid, mp:169.4-170.1 °C. ^1^H NMR (400 MHz, DMSO-*d*_6_) δ 9.76 (s, 1H), 8.26 (s, 1H), 8.10 (d, *J* = 2.3 Hz, 1H), 7.68 (d, *J* = 2.4 Hz, 1H), 7.44 (d, *J* = 8.9 Hz, 1H), 6.97 (s, 1H), 2.20 (q, *J* = 7.3 Hz, 2H), 2.04 (s, 3H), 1.74 (s, 3H), 0.91 (d, *J* = 7.5 Hz, 3H). ^13^C NMR (100 MHz, DMSO-*d*_6_) δ 157.32(C=O), 152.24, 140.16, 132.55, 129.04, 128.94, 126.11, 125.42 (q, *J* = 287.8 Hz, C-CF_3_), 122.89, 121.14, 120.69, 120.03, 118.87, 118.26, 74.98 (q, *J* = 30.0 Hz, CF_3_), 17.43, 16.19, 11.09, 10.04. ^19^F NMR (376 MHz, DMSO-*d*_6_) δ -73.50(CF_3_). HRMS (APCI): *m/z* calculated for C_19_H_16_ClF_3_NO_3_ [M-H]^−^ 398.0771, found: 398.0751.

6-Bromo-3-(1-(4-ethyl-3,5-dimethyl-1H-pyrrol-2-yl)-2,2,2-trifluoro-1-hydroxyethyl)-2H-chromen-2-one (**3dd**): Yellow solid, mp: 168.6–169.2 °C. ^1^H NMR (400 MHz, CDCl_3_) δ 8.06 (s, 1H), 7.77–7.67 (m, 2H), 7.27 (d, *J* = 9.6 Hz, 1H), 6.14 (s, 1H), 2.33 (q, *J* = 7.6 Hz, 2H), 2.18 (s, 3H), 1.81 (s, 3H), 1.01 (t, *J* = 7.5 Hz, 3H). ^13^C NMR (100 MHz, CDCl_3_) δ 160.99 (C=O), 151.97, 141.31, 135.69, 131.29, 124.67, 124.51 (q, *J* = 286.8 Hz, C-CF_3_), 123.48, 122.54, 119.76, 118.36, 117.90, 117.71, 116.77, 76.66 (q, *J* = 30.9 Hz, CF_3_), 17.49, 15.55, 11.20, 10.08. ^19^F NMR (376 MHz, CDCl_3_) δ -75.34(CF_3_). HRMS (APCI): *m/z* calculated for C_19_H_16_BrF_3_NO_3_ [M-H]^−^ 442.0266, found: 442.0243.

3-(1-(4-Ethyl-3,5-dimethyl-1H-pyrrol-2-yl)-2,2,2-trifluoro-1-hydroxyethyl)-7-methoxy-2H-chromen-2-one (**3ed**): Cyan solid, mp: 160.4–161.1 °C. ^1^H NMR (400 MHz, DMSO-*d*_6_) δ 9.77 (s, 1H), 8.18 (s, 1H), 7.86 (d, *J* = 8.6 Hz, 1H), 7.03–6.97 (m, 2H), 6.79 (s, 1H), 3.86 (s, 3H), 2.25–2.19 (m, 2H), 2.06 (s, 3H), 1.74 (s, 3H), 0.93 (t, *J* = 7.5 Hz, 3H). ^13^C NMR (101 MHz, DMSO-*d*_6_) δ 163.39(C=O), 158.27, 155.58, 141.29, 131.12, 125.63 (d, *J* = 290.7 Hz, C-CF3), 122.61, 121.36, 120.53, 119.44, 114.52, 113.20, 112.15, 100.56, 74.99 (d, *J* = 29.0 Hz, CF_3_), 56.50, 17.50, 16.24, 11.14, 10.10. ^19^F NMR (376 MHz, DMSO-*d*_6_) δ -75.42(CF_3_). HRMS (APCI): *m/z* calculated for C_20_H_19_F_3_NO_4_ [M-H]^−^ 394.1266, found: 394.1247.

6,8-Dichloro-3-(1-(4-ethyl-3,5-dimethyl-1H-pyrrol-2-yl)-2,2,2-trifluoro-1-hydroxyethyl)-2H-chromen-2-one (**3gd**): Dark yellow solid, mp: 145.8–146.5 °C. ^1^H NMR (400 MHz, DMSO-*d*_6_) δ 9.77 (s, 1H), 8.33 (s, 1H), 8.17 (d, *J* = 2.3 Hz, 1H), 8.00 (d, *J* = 2.3 Hz, 1H), 7.04 (s, 1H), 2.23 (q, *J* = 7.4 Hz, 2H), 2.06 (s, 3H), 1.80 (s, 3H), 0.93 (d, *J* = 7.5 Hz, 3H). ^13^C NMR (100 MHz, DMSO-*d*_6_) δ 156.23(C=O), 148.16, 140.16, 131.95, 128.85, 128.31, 126.98, 125.38 (q, *J* = 287.6 Hz, C-CF3), 122.98, 121.15, 120.88, 118.73, 115.10, 74.95 (q, *J* = 29.7 Hz, CF_3_), 17.45, 16.20, 11.13, 11.09, 10.14. ^19^F NMR (376 MHz, DMSO-*d*_6_) δ -73.30. HRMS (APCI): *m/z* calculated for C_19_H_15_Cl_2_F_3_NO_3_ [M-H]^−^ 432.0381, found: 432.0357.

6,8-Dibromo-3-(1-(4-ethyl-3,5-dimethyl-1H-pyrrol-2-yl)-2,2,2-trifluoro-1-hydroxyethyl)-2H-chromen-2-one (**3hd**): Dark yellow solid, mp: 142.2–142.9 °C.^1^H NMR (400 MHz, DMSO-*d*_6_) δ 7.97 (s, 1H), 7.88 (d, *J* = 2.2 Hz, 1H), 7.65 (s, 1H), 7.60 (d, *J* = 2.2 Hz, 1H), 5.88 (s, 1H), 2.29–2.23 (m, 2H), 2.11 (s, 3H), 1.74 (s, 3H), 0.95 (t, *J* = 7.5 Hz, 3H). ^13^C NMR (100 MHz, DMSO-*d*_6_) δ 156.40(C=O), 149.64, 140.11, 137.24, 131.84, 126.84, 122.97, 122.54 (q, *J* = 287.5 Hz, C-CF_3_), 120.69, 118.75, 116.78, 115.09, 110.16, 74.94 (q, *J* = 29.8 Hz, CF_3_), 17.46, 16.21, 11.13, 11.09, 10.15. ^19^F NMR (376 MHz, DMSO-*d*_6_) δ -73.32(CF_3_). HRMS (APCI): *m/z* calculated for C_19_H_15_Br_2_F_3_NO_3_ [M-H]^−^ 521.9350, found: 521.9319.

3-(2,2,2-Trifluoro-1-hydroxy-1-(1H-pyrrol-2-yl)-ethyl)-2H-chromen-2-one (**3ae**): White solid, mp: 153.5-154.3 °C. ^1^H NMR (400 MHz, CDCl_3_) δ 8.80 (s, 1H), 7.84 (s, 1H), 7.62 (t, *J* = 7.9 Hz, 1H), 7.50 (s, 1H), 7.44–7.34 (m, 3H), 7.26 (s, 1H), 6.86 (s, 1H), 6.40 (s, 1H), 6.27 (s, 1H). ^13^C NMR (100 MHz, CDCl_3_) δ 162.44(C=O), 154.19, 146.88, 135.26, 129.13, 125.89, 125.20, 124.45 (q, *J* = 286.5 Hz, C-CF3), 118.81, 118.33, 116.62 110.00, 109.06, 108.40, 76.69 (q, *J* = 31.4 Hz, CF_3_). ^19^F NMR (376 MHz, CDCl_3_) δ -78.07(CF_3_). HRMS (APCI): m/z calculated for C_15_H_9_F_3_NO_2_ [M-H]^−^ 308.0540, found: 308.0537.

3-(2,2,2-Trifluoro-1-hydroxy-1-(1-methyl-1H-pyrrol-2-yl)ethyl)-2H-chromen-2-one (**3af)**: White solid, mp: 129.3-131.1 °C. ^1^H NMR (400 MHz, CDCl_3_) δ 7.66–7.60 (m, 1H), 7.46–7.40 (m, 2H), 7.36–7.31 (m, 2H), 7.21 (s, 1H), 6.70–6.64 (m, 1H), 6.46 (s, 1H), 6.17–6.10 (m, 1H), 3.55 (s, 3H). ^13^C NMR (100 MHz, CDCl_3_) δ 162.22 (C=O), 153.42, 146.17, 133.39, 129.06, 125.76, 125.35, 124.87, 124.71 (q, *J* = 287.8 Hz, C-CF_3_), 122.31, 118.39, 116.69, 111.43, 106.56, 77.85 (q, *J* = 30.6 Hz, *C*F_3_), 36.14. ^19^F NMR (376 MHz, CDCl_3_) δ -77.07(CF_3_). HRMS (APCI): *m/z* calculated for C_16_H_11_F_3_NO_2_ [M-OH]^+^ 306.0737, found: 306.0747.

### 3.2. In Vitro Antifungal Assay

Antifungal assays were performed against *F. graminearum* (from corn)*, F. oxysporum, F. graminearum* (from wheat)*, F. moniliforme, R. solani Kuhn,* and *P. parasitica var nicotianae* in vitro by the plate growth rate method. The synthesized compounds were dissolved in 2% DMSO to yield a 10 mg/mL stock solution. Then, each solution was added to sterile potato dextrose agar (PDA) to give final concentrations of 0.1 mg/mL. After the mixture was chilled, the mycelium of the fungi was transferred to the test plate and incubated at 26 °C. When the mycelium of the fungi reached the edges of the control plate (without sample), the inhibitory index was calculated as follows: Inhibitory index (%) = (Db − Da)/(Db − Dc) × 100%, where Da is the colony diameter of the growth zone in the test plate, Db is the colony diameter of the growth zone in the control plate, and Dc is the diameter of the mycelial disc. The median effective concentration (EC_50_) of each compound with a significant fungicidal activity was further evaluated in three independent experiments. The statistical analyses were performed using SPSS software (IBM SPSS Statistic 26).

### 3.3. Molecular Docking

The molecular docking studies of compound **3cd** and triadimefon were performed with the assistance of Discovery Studio 2016 software and pymol. The crystal structure of Tre was acquired from the Research Collaboratory for Structural Bioinformatics (RCSB) Protein Data Bank (PDB code 2JF4). The ligand validoxylamine was extracted, and all water molecules were eliminated from this crystal complex. Libdock was applied for simulating and evaluating the interactions between the compounds and the target protein by an empirical scoring function.

## 4. Conclusions

In conclusion, we have developed a new and practical catalyst-free method for novel α-trifluoromethylated tertiary alcohols bearing coumarins at ambient temperature. This procedure means a promising approach for attaining these new heterocycles, since it applies mild and easily operational conditions (no special reagents, catalysts, or additives). Altogether, 29 new α-trifluoromethylated tertiary alcohols were synthesized in high to excellent yields. The crystal structure of compound 3fa was studied by single-crystal XRD analysis. The biological activity of the α-trifluoromethylated tertiary alcohols was also tested in in vitro antifungal assays. The bioassay results indicated that the compounds showed broad-spectrum fungicidal activity in vitro.

## Figures and Tables

**Table 1:** Optimization of the reaction 3-(trifluoroacetyl)coumarin **1a** and 2-methylpyrrole **2a** ^a^.

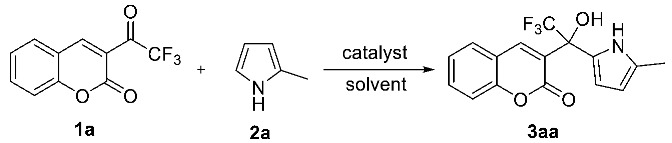				
Entry	Catalyst	Solvent	Temp. (℃)	Yield (%) ^b^
**1**	AlCl_3_ (5%)	CH_2_Cl_2_	25	46
**2**	FeCl_3_ (5%)	CH_2_Cl_2_	25	39
**3**	CuCl_2_ (5%)	CH_2_Cl_2_	25	41
**4**	CH_3_COOAg (5%)	CH_2_Cl_2_	25	40
**5**	Sc(OTf)_3_ (5%)	CH_2_Cl_2_	25	20
**6**	Cu(OTf)_2_ (5%)	CH_2_Cl_2_	25	46
**7**	—	CH_2_Cl_2_	25	97
**8**	—	CHCl_3_	25	48
**9**	—	EtOAc	25	20
**10**	—	CH_3_CN	25	35
**11**	—	toluene	25	21
**12**	—	THF	25	36
**13**	—	dioxane	25	55
**14**	—	ClCH_2_CH_2_Cl	25	78
**15**	—	BrCH_2_CH_2_Br	25	68
**16**	—	CH_2_Cl_2_	0	36

^a^ Reactions were performed with **1a** (1.0 mmol) and **2a** (1.0 mmol), with catalyst (1%) or without catalyst in the solvent (1.5 mL). ^b^ Isolated yield.

**Table 2:** Reaction scope of the Friedel–Crafts reaction of 3-(trifluoroacetyl)coumarin **1** with pyrroles **2** ^a^.

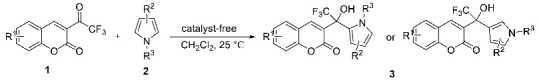					
Entry	R^1^	R^2^	R^3^	Product	Yield ^b^
**1**	H	2-Me	H	**3aa**	97
**2**	6-Me	2-Me	H	**3ba**	85
**3**	6-Cl	2-Me	H	**3ca**	91
**4**	6-Br	2-Me	H	**3da**	84
**5**	7-OMe	2-Me	H	**3ea**	97
**6**	8-OMe	2-Me	H	**3fa**	85
**7**	6,8-(Cl)_2_	2-Me	H	**3ga**	95
**8**	6,8-(Br)_2_	2-Me	H	**3ha**	90
**9**	naphthyl	2-Me	H	**3ia**	89
**10**	H	2,4-(Me)_2_	H	**3ab**	93
**11**	8-OMe	2,4-(Me)_2_	H	**3fb**	84
**12 ^c^**	H	2,5-(Me)_2_	H	**3ac**	78
**13 ^c^**	6-Me	2,5-(Me)_2_	H	**3bc**	85
**14 ^c^**	6-Cl	2,5-(Me)_2_	H	**3cc**	88
**15 ^c^**	6-Br	2,5-(Me)_2_	H	**3dc**	86
**16 ^c^**	7-OMe	2,5-(Me)_2_	H	**3ec**	91
**17 ^c^**	8-OMe	2,5-(Me)_2_	H	**3fc**	85
**18 ^c^**	6,8-(Cl)_2_	2,5-(Me)_2_	H	**3gc**	90
**19 ^c^**	6,8-(Br)_2_	2,5-(Me)_2_	H	**3hc**	87
**20 ^c^**	naphthyl	2,5-(Me)_2_	H	**3ic**	82
**21**	H	2,4-(Me)_2_-3-Et	H	**3ad**	92
**22**	6-Me	2,4-(Me)_2_-3-Et	H	**3bd**	89
**23**	6-Cl	2,4-(Me)_2_-3-Et	H	**3cd**	92
**24**	6-Br	2,4-(Me)_2_-3-Et	H	**3dd**	87
**25**	7-OMe	2,4-(Me)_2_-3-Et	H	**3ed**	92
**26**	6,8-(Cl)_2_	2,4-(Me)_2_-3-Et	H	**3gd**	86
**27**	6,8-(Br)_2_	2,4-(Me)_2_-3-Et	H	**3hd**	75
**28**	H	H	H	**3ae**	77
**29**	H	H	Me	**3af**	73
**30**	H	2-COOH	H	**—**	NR ^d^

^a^ Reactions were performed with **1a** (1.0 mmol) and **2a** (1.0 mmol) in the CH_2_Cl_2_ (10 mL). ^b^ Isolated yield. ^c^ The reaction was performed with Sc(OTf)_3_ (5%) at 45 ℃. ^d^ NR=No reaction.

**Table 3:** In Vitro Fungicidal Activity of Target Compounds against Phytopathogens ^a–c^.

Compound	A	B	C	D	E	F
**3aa**	31 ± 0	24 ± 0	<10	26 ± 3	64 ± 1	17 ± 1
**3ba**	25 ± 3	28 ± 0	<10	19 ± 2	58 ± 2	15 ± 0
**3ca**	<10	<10	<10	16 ± 1	44 ± 1	<10
**3da**	20 ± 1	21 ± 0	<10	14 ± 0	54 ± 1	<10
**3ea**	27 ± 1	24 ± 1	<10	14 ± 0	47 ± 0	21 ± 0
**3fa**	<10	<10	<10	<10	50 ± 1	<10
**3ga**	37 ± 2	73 ± 1	<10	22 ± 1	54 ± 0	21 ± 1
**3ha**	59 ± 2	36 ± 1	<10	55 ± 1	64 ± 0	79 ± 0
**3ia**	20 ± 0	17 ± 0	<10	29 ± 0	53 ± 1	43 ± 1
**3ab**	75 ± 3	67 ± 1	45 ± 2	74 ± 0	86 ± 2	64 ± 0
**3ac**	77 ± 0	<10	<10	77 ± 0	54 ± 1	82 ± 1
**3bc**	36 ± 1	48 ± 1	<10	40 ± 2	47 ± 0	20 ± 0
**3cc**	47 ± 3	56 ± 1	<10	55 ± 1	70 ± 0	61 ± 1
**3dc**	34 ± 0	42 ± 0	<10	46 ± 1	61 ± 1	29 ± 1
**3ec**	40 ± 0	49 ± 0	28 ± 0	51 ± 0	58 ± 0	40 ± 1
**3fc**	53 ± 2	45 ± 0	24 ± 0	46 ± 0	49 ± 0	45 ± 1
**3gc**	64 ± 1	56 ± 0	47 ± 0	56 ± 0	80 ± 2	50 ± 2
**3hc**	60 ± 2	50 ± 0	45 ± 1	43 ± 1	60 ± 1	32 ± 0
**3ic**	28 ± 1	24 ± 0	20 ± 0	33 ± 1	54 ± 1	15 ± 1
**3ad**	97 ± 0	84 ± 2	87 ± 0	82 ± 0	98 ± 1	88 ± 1
**3bd**	95 ± 0	88 ± 0	86 ± 1	62 ± 1	78 ± 2	85 ± 1
**3cd**	94 ± 2	69 ± 1	76 ± 1	62 ± 1	86 ± 1	57 ± 1
**3dd**	76 ± 2	58 ± 1	35 ± 1	47 ± 3	54 ± 1	35 ± 3
**3ed**	64 ± 2	73 ± 1	71 ± 1	52 ± 1	74 ± 1	33 ± 1
**3gd**	87 ± 2	93 ± 1	62 ± 2	94 ± 1	100	87 ± 1
**3hd**	89 ± 2	84 ± 1	53 ± 1	55 ± 0	100	88 ± 1
**triadimefon**	45 ± 1	41 ± 1	25 ± 1	85 ± 1	70 ± 1	30 ± 1

^a^ **A**: Fusarium graminearum (from corn), **B**: Fusarium oxysporum, **C**: Fusarium graminearum (from wheat), **D**: Fusarium moniliforme, **E**: Rhizoctonia solani Kuhn, **F**: Phytophthora parasitica var nicotianae. ^b^ Values are means of three replicates. ^c^ The compounds exhibited a certain degree of fungicidal activity at a concentration of 500 μg/mL.

**Table 4:** In Vitro Potency (EC_50_) of Compounds with Higher Preliminary Activities ^a^.

Fungicidal	Compound	Regression Equation	R^2^	EC_50_ (μg/mL)
*F. graminearum*	**3ad**	y = 0.401x + 1.007	0.941	54.4
	**3bd**	y = 0.445x + 0.930	0.930	107.8
	**triadimefon**	y = 0.346x + 1.181	0.974	10.7
*F. oxysporum*	**3hd**	y = 0.434x + 0.941	0.947	96.7
	**triadimefon**	y = 0.498x + 1.181	0.990	42.9
*F. moniliforme*	**3gd**	y = 0.654x + 1.182	0.937	90.9
	**triadimefon**	y = 0.234x + 1.194	0.977	1.1
*R. solani Kuhn*.	**3ad**	y = 0.317x + 1.032	0.971	21.0
	**3cd**	y = 0.226x + 0.944	0.985	10.9
	**3gd**	y = 0.321x + 1.065	0.979	17.4
	**3hd**	y = 0.339x + 1.075	0.993	20.2
	**triadimefon**	y = 0.329x + 1.227	0.974	6.1
*P. parasitica var nicotianae*	**3gd**	y = 0.616x + 1.074	0.994	117.0
	**3hd**	y = 0.440x + 1.027	0.964	63.4
	**triadimefon**	y = 0.248x + 0.745	0.984	102.3

^a^ Values are means of three replicates.

## Data Availability

Not applicable.

## Supplementary Materials

The following supporting information can be downloaded at: https://www.mdpi.com/article/10.3390/molecules28010260/s1, Figure S1: Molecular packing of compound **3fa** (CCDC 2167896); Table S1: The refinement information and crystallographic data of **3fa**; and Figures S2–S88: ^1^H-NMR, ^13^C-NMR and ^19^F-NMR spectra of compounds **3aa**–**3fa**. **Figures S89–S117**: HRMS spectra of compounds **3aa**–**3fa**.
