# Supplementary material for: Design, Catalyst-Free Synthesis of New Novel α-Trifluoromethylated Tertiary Alcohols Bearing Coumarins as Potential Antifungal Agents
Source: Molecules. 2022 Dec 28;28(1):260. doi: 10.3390/molecules28010260 (PMC9822406; doi:10.3390/molecules28010260)

Supplementary Information

Table of Contents

1. **Single crystal X-ray diffraction measurements** **S1**
2. **^{1}H NMR, ^{13}C NMR and ^{19}F NMR spectra** **S3**
3. **HRMS spectra** **S47**

Single crystal X-ray diffraction measurements

The crystallographic data are listed in Table S1. Deposition Numbers CCDC 2167896 contain the supplementary crystallographic data for this paper. These data are provided free of charge by the joint Cambridge Crystallographic Data Centre and Fachinformationszentrum Karlsruhe Access Structures service www.ccdc.cam.ac.uk/structures.

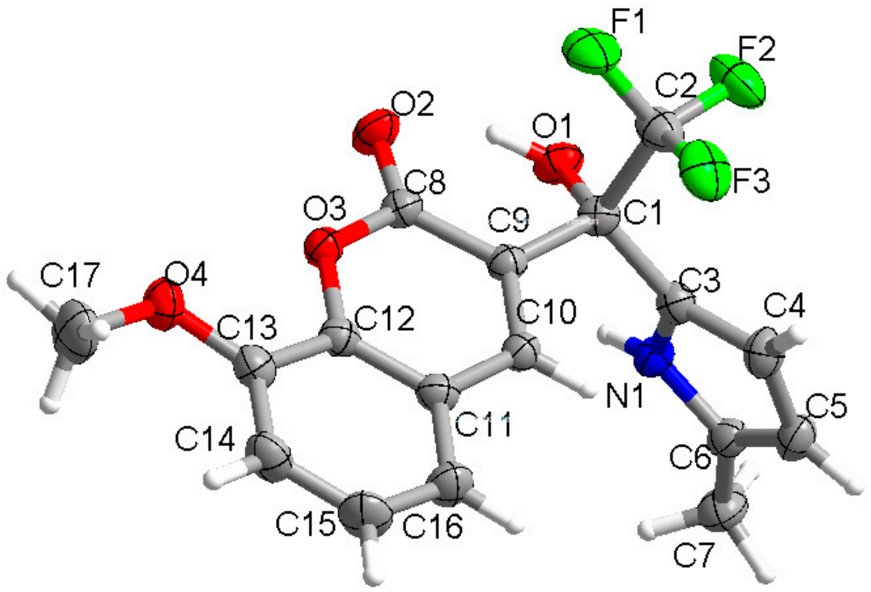

Figure S1.

Table S1 Crystal data and structure refinement for compound **3fa**.

	**3fa**
**CCDC number**	2167896
**Empirical formula**	$C_{17}H_{14}F_3NO_4$
**Foimula weight**	353.29
**T/K**	293(2)
**Crystal system**	monoclinic
**Space group**	$P2_1/n$
***a*/Å**	12.8173(4)
***b*/Å**	9.7642(3)

***c*/Å**	12.8760(4)
***α*/°**	90
***β*/°**	99.231(3)
***γ*/°**	90
***V*/Å³**	1590.57(9)
Z	4
**D_{calc} /g cm^{-3}**	1.475
**μ/mm^{-1}**	1.112
**F(000)**	728.0
**Crystal size/mm^3**	0.14 × 0.1 × 0.08
**Radiation**	CuKα (λ = 1.54184)
**2Θ range for data collection/°**	9.038 to 134.154
**Index ranges**	-15 ≤ h ≤ 15, -11 ≤ k ≤ 11, -9 ≤ l ≤ 15
**Reflections collected**	11054
**Data/restraints/parameters**	2842/0/237
**Independent reflections**	2842 [R_{int} = 0.0341, R_{sigma} = 0.0274]
**Goodness-of-fit on F^2**	1.037
**Final R indexes [I>=2σ (I)]**	R_1 = 0.0406, wR_2 = 0.1127
**Final R indexes [all data]**	R_1 = 0.0516, wR_2 = 0.1232
**Largest diff. peak/hole / e Å^{-3}**	0.17/-0.18

^{1}H NMR, ^{13}C NMR and ^{19}F NMR spectra

Figure S2. ^{1}H NMR spectrum of the compound (**3aa**)

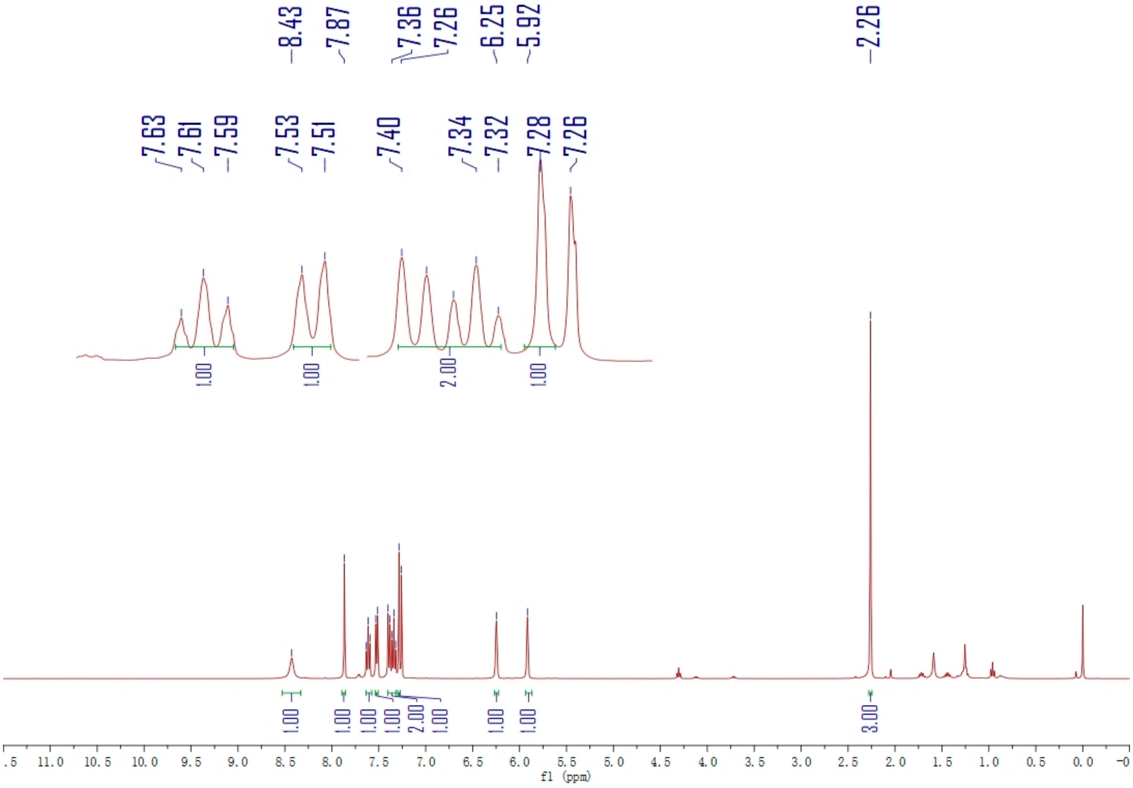

Figure S3. ^{13}C NMR spectrum of the compound (**3aa**)

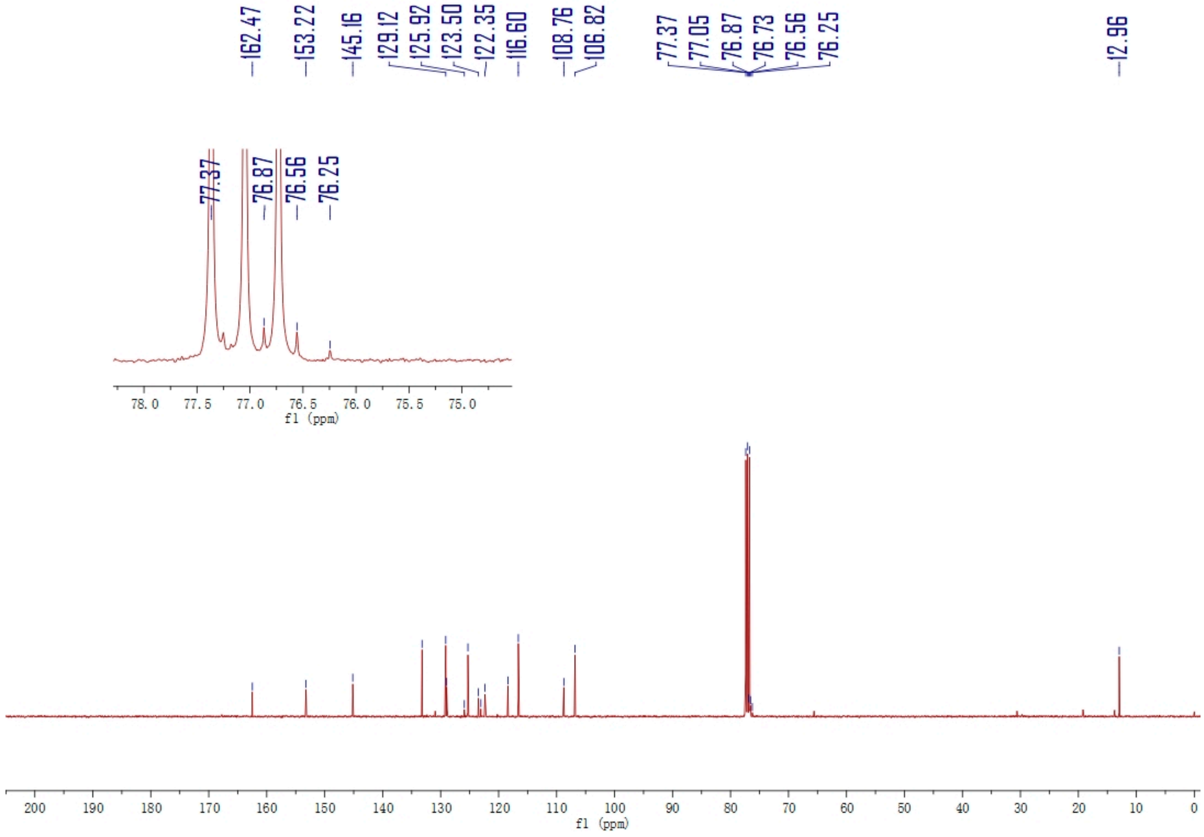

Figure S4. ^{19}F NMR spectrum of the compound(**3aa**)

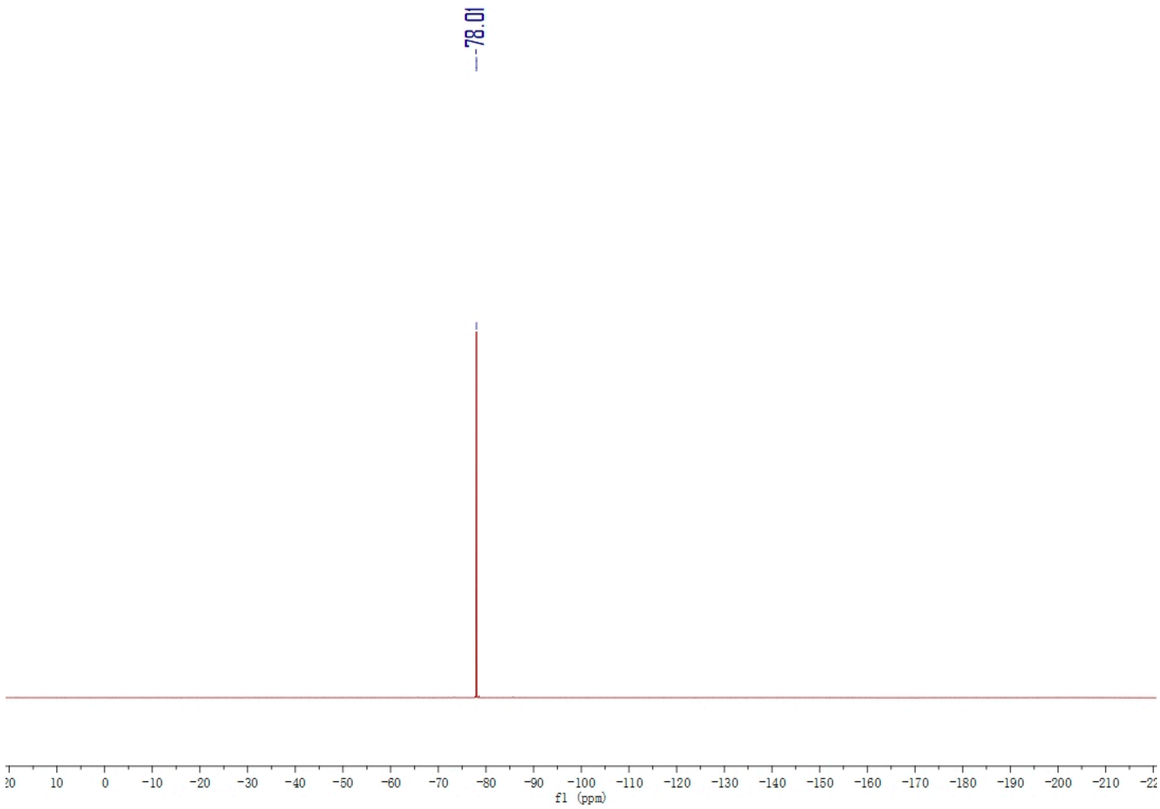

Figure S5. ^{1}H NMR spectrum of the compound (**3ba**)

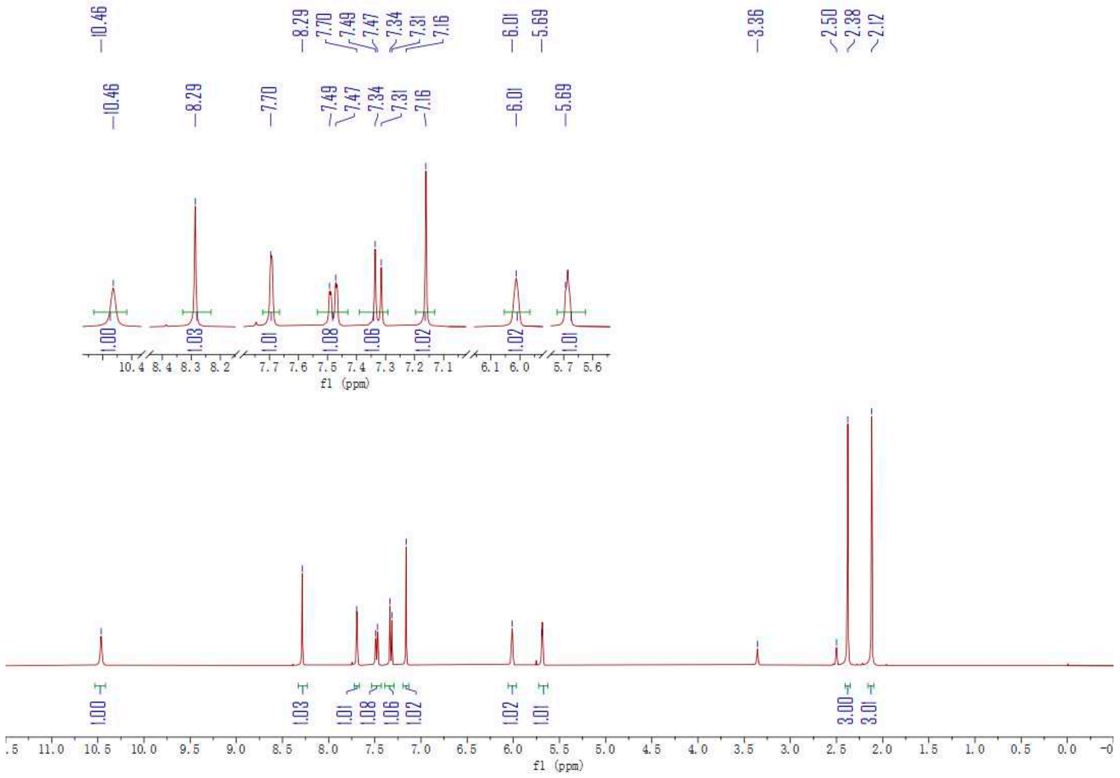

Figure S6. ^{13}C NMR spectrum of the compound (**3ba**)

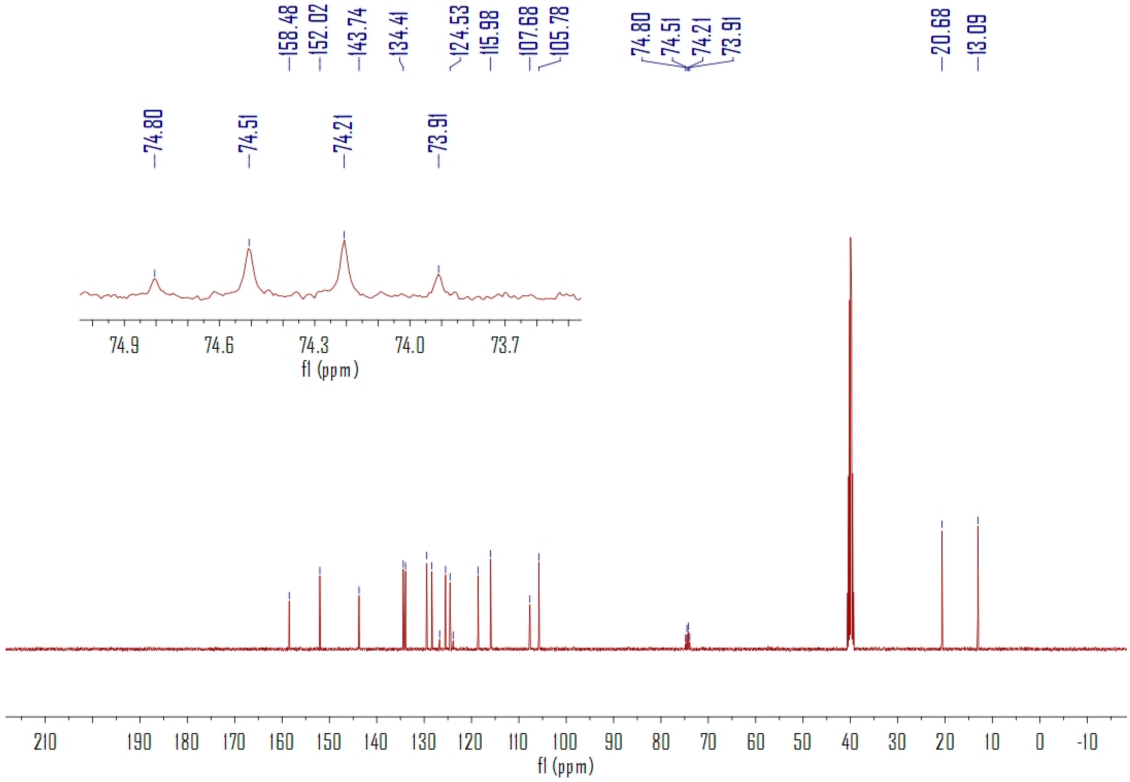

Figure S7. ^{19}F NMR spectrum of the compound (**3ba**)

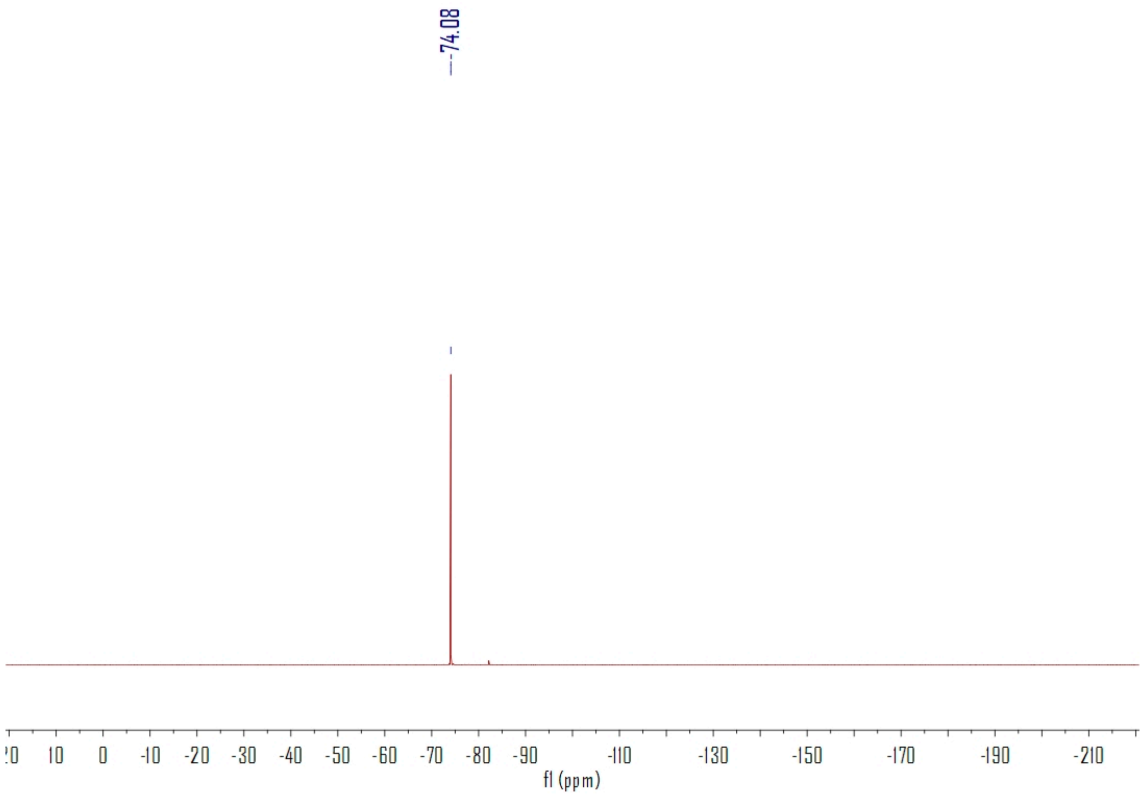

Figure S8. ^{1}H NMR spectrum of the compound (**3ca**)

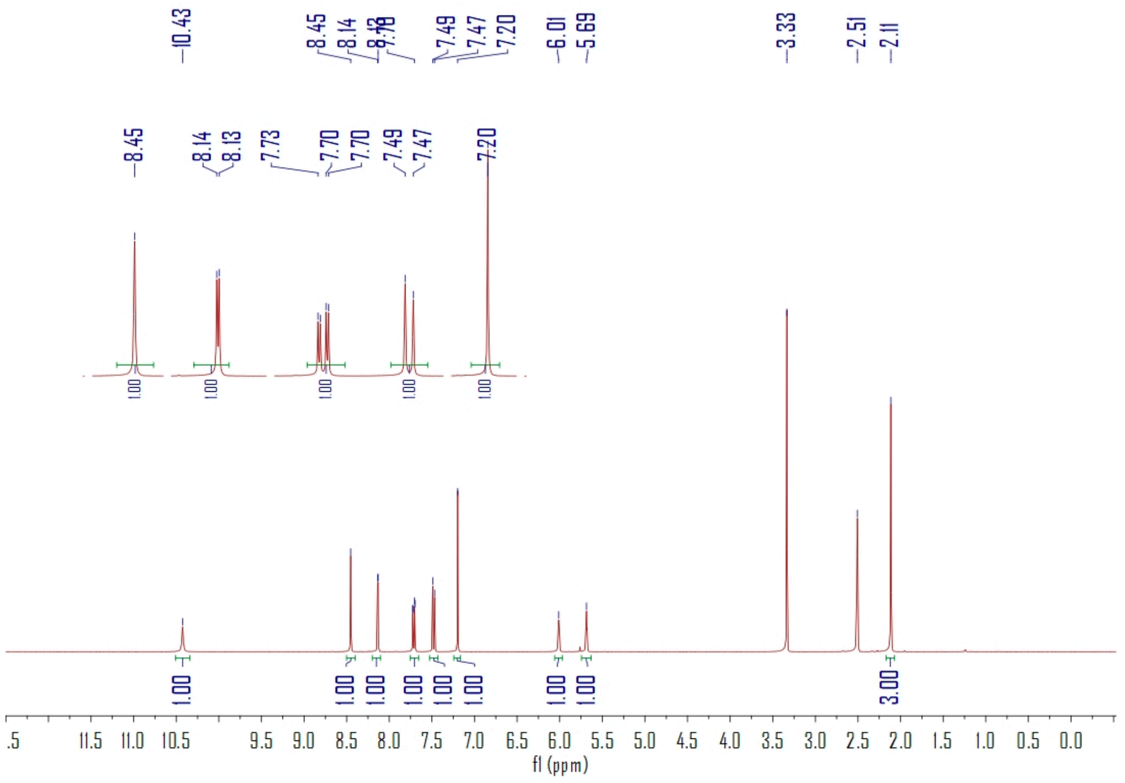

Figure S9. ^{13}C NMR spectrum of the compound (**3ca**)

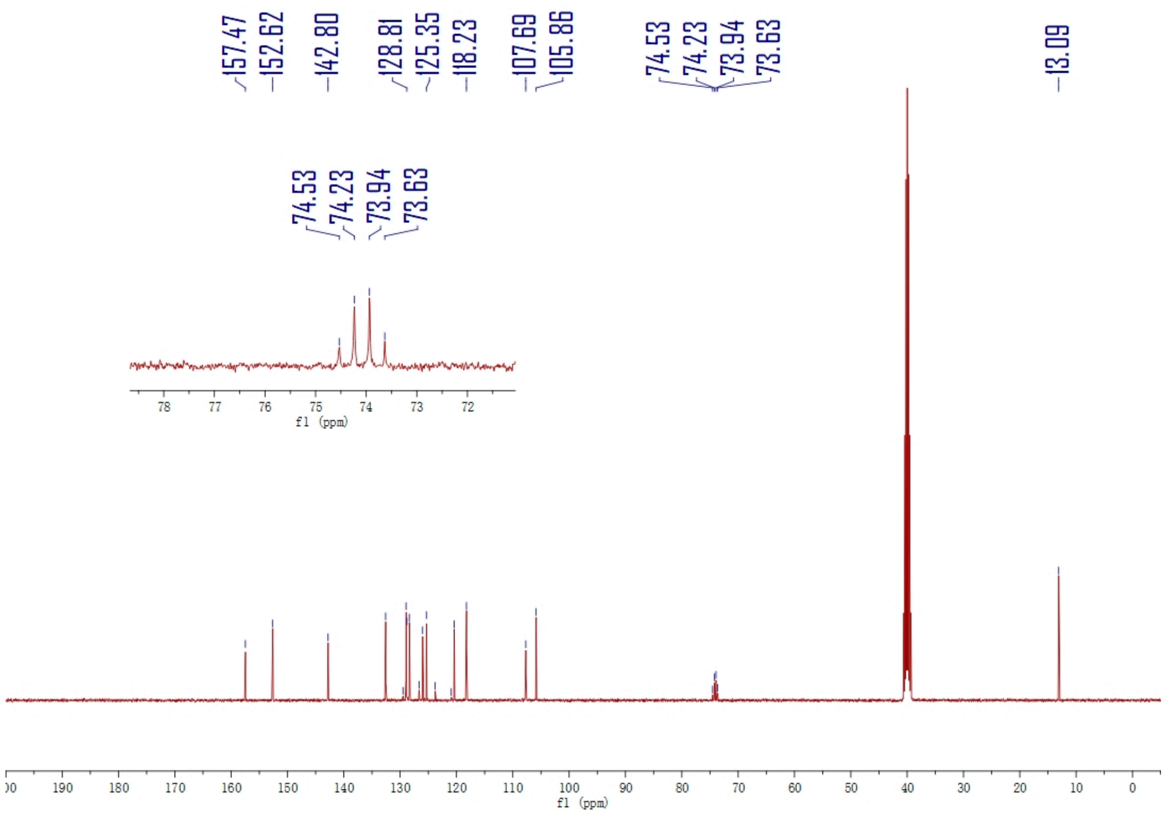

Figure S10. ^{19}F NMR spectrum of the compound(**3ca**)

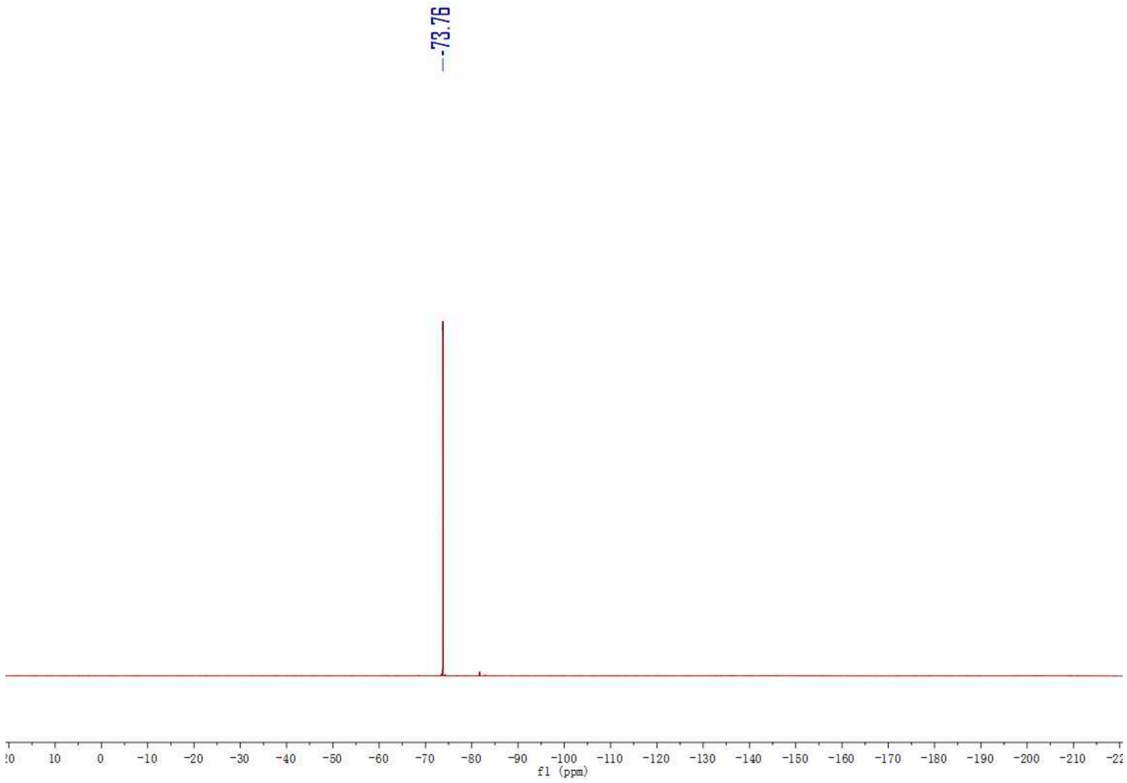

Figure S11. ^{1}H NMR spectrum of the compound (**3da**)

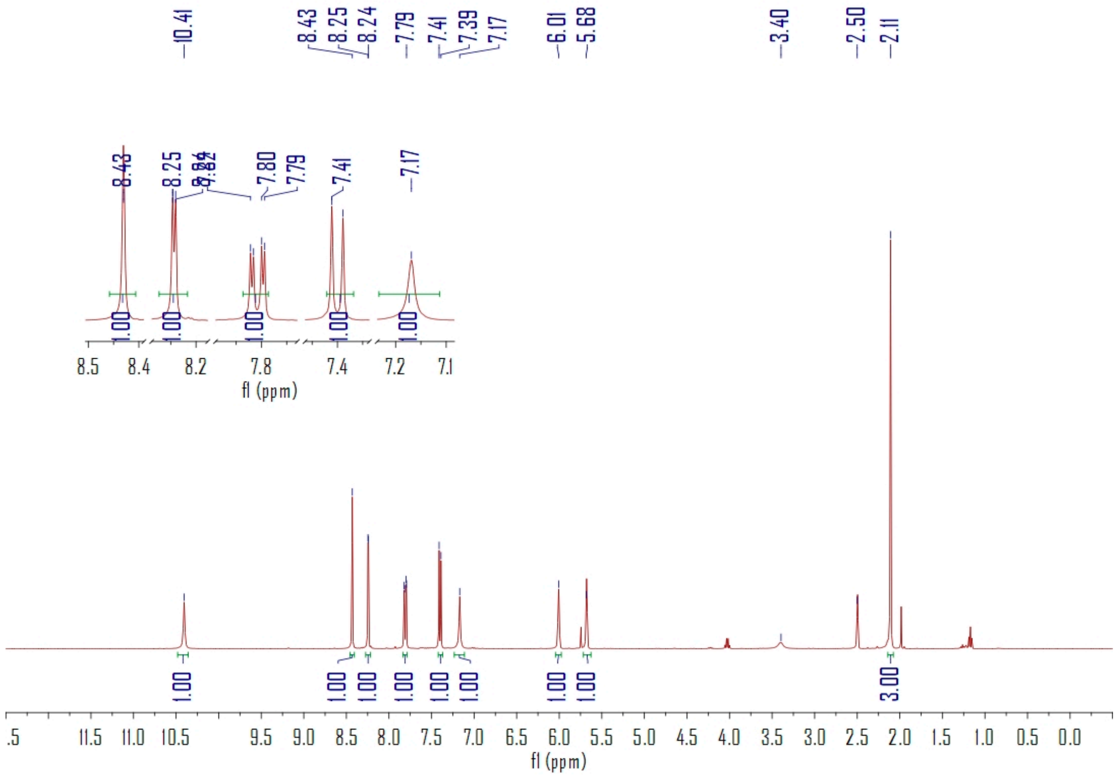

Figure S12. ^{13}C NMR spectrum of the compound (**3da**)

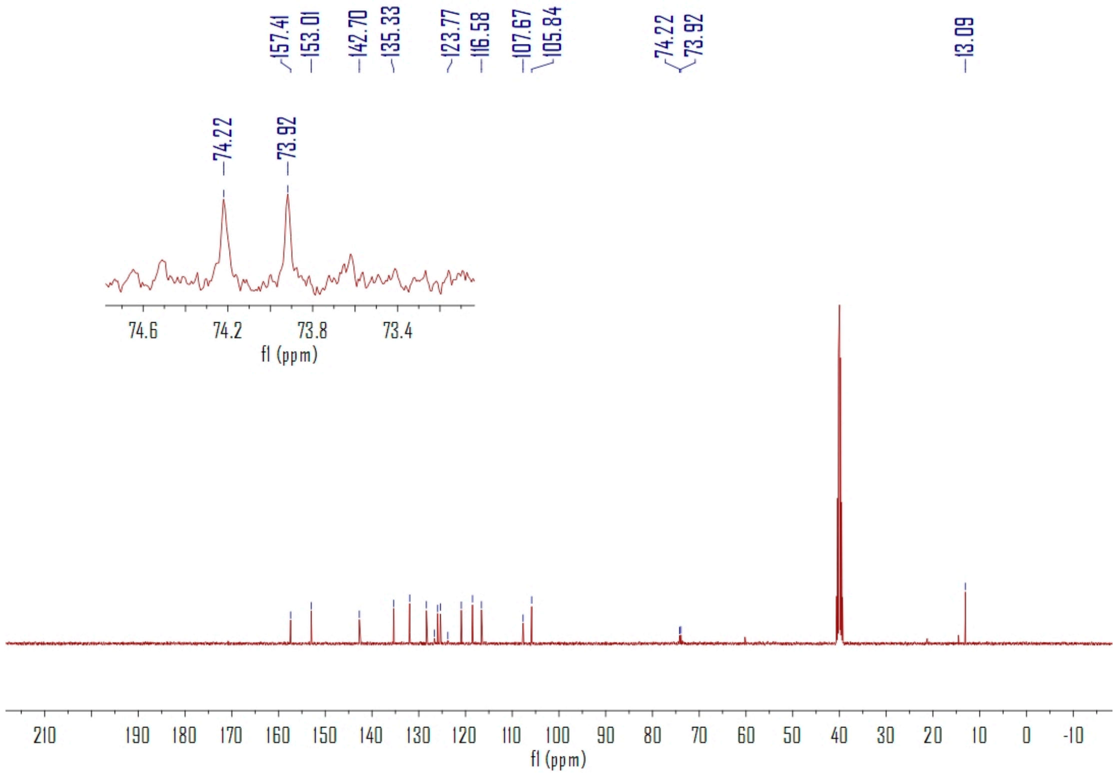

Figure S13. ^{19}F NMR spectrum of the compound (**3da**)

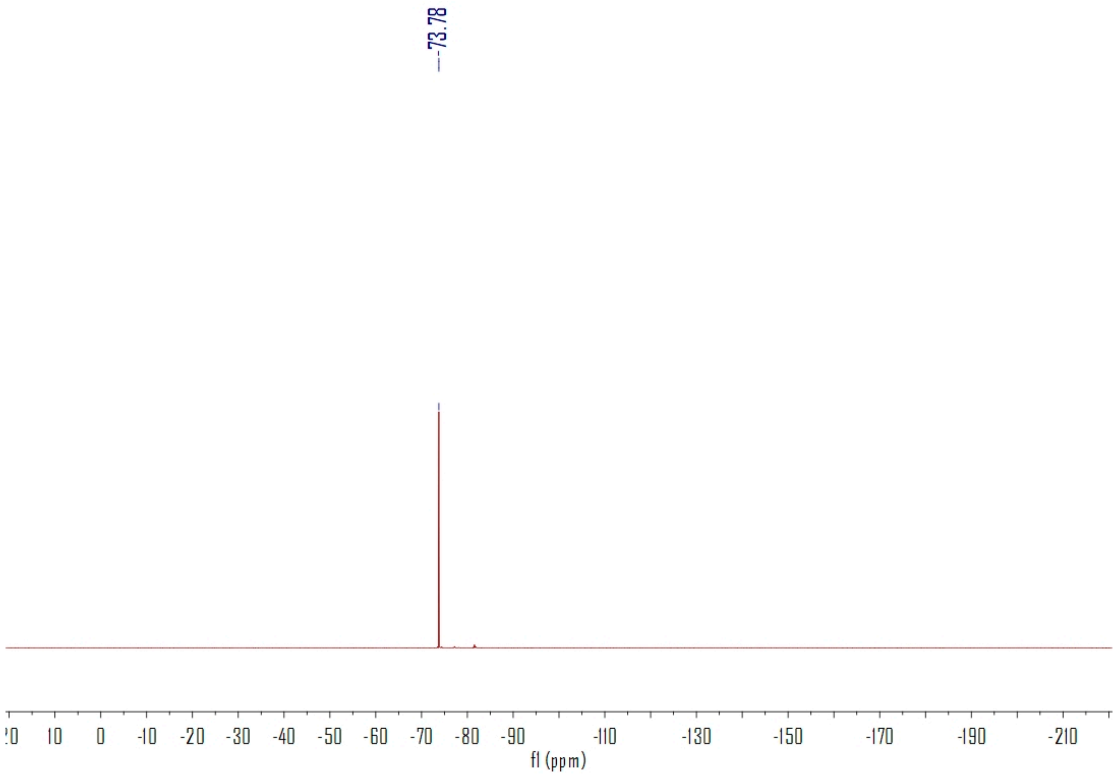

Figure S14. ^{1}H NMR spectrum of the compound (**3ea**)

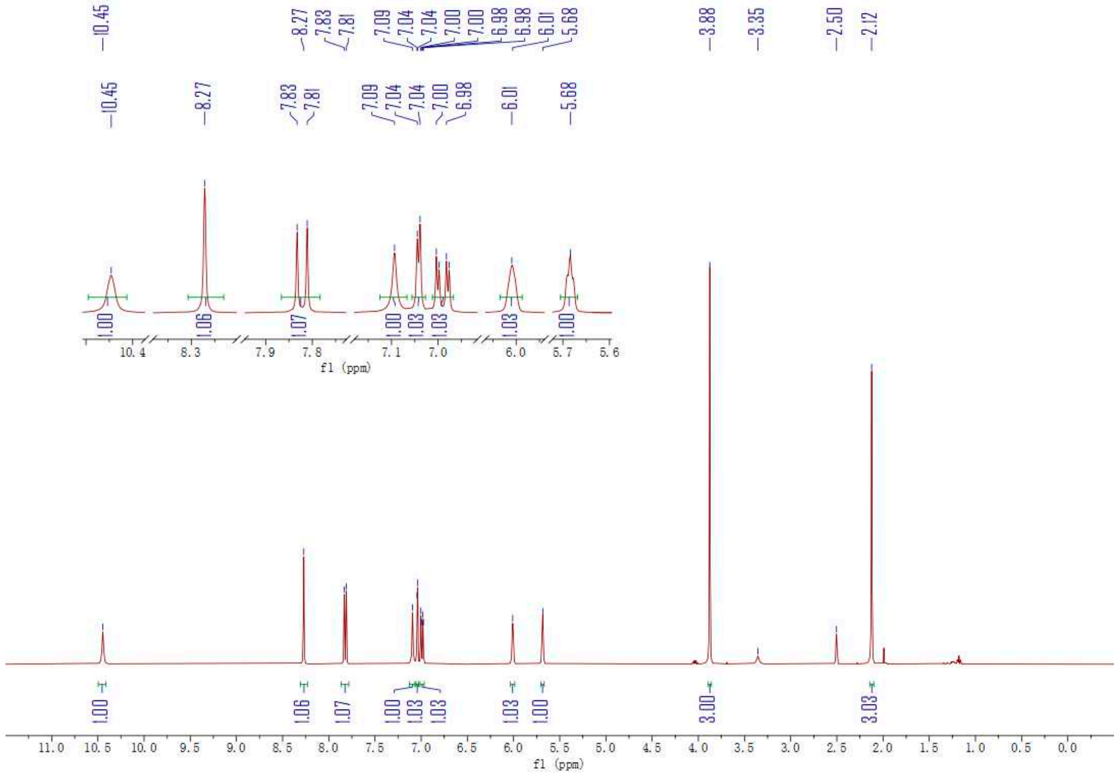

Figure S15. ^{13}C NMR spectrum of the compound(**3ea**)

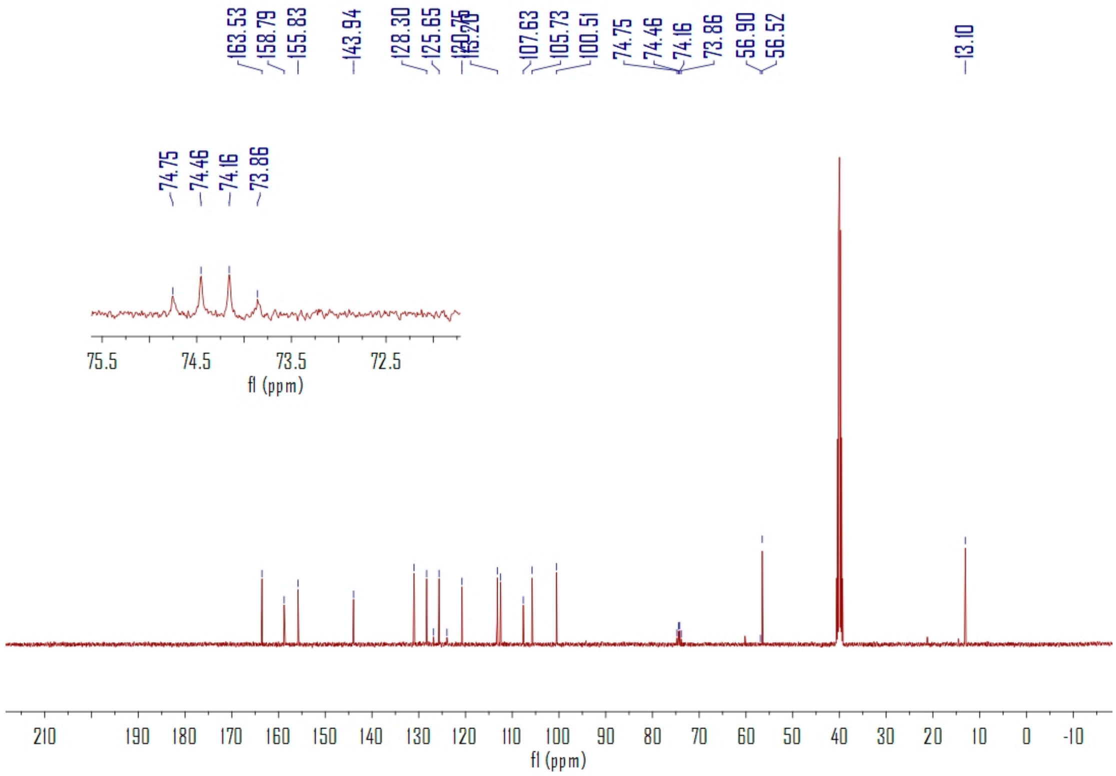

Figure S16. ^{19}F NMR spectrum of the compound (**3ea**)

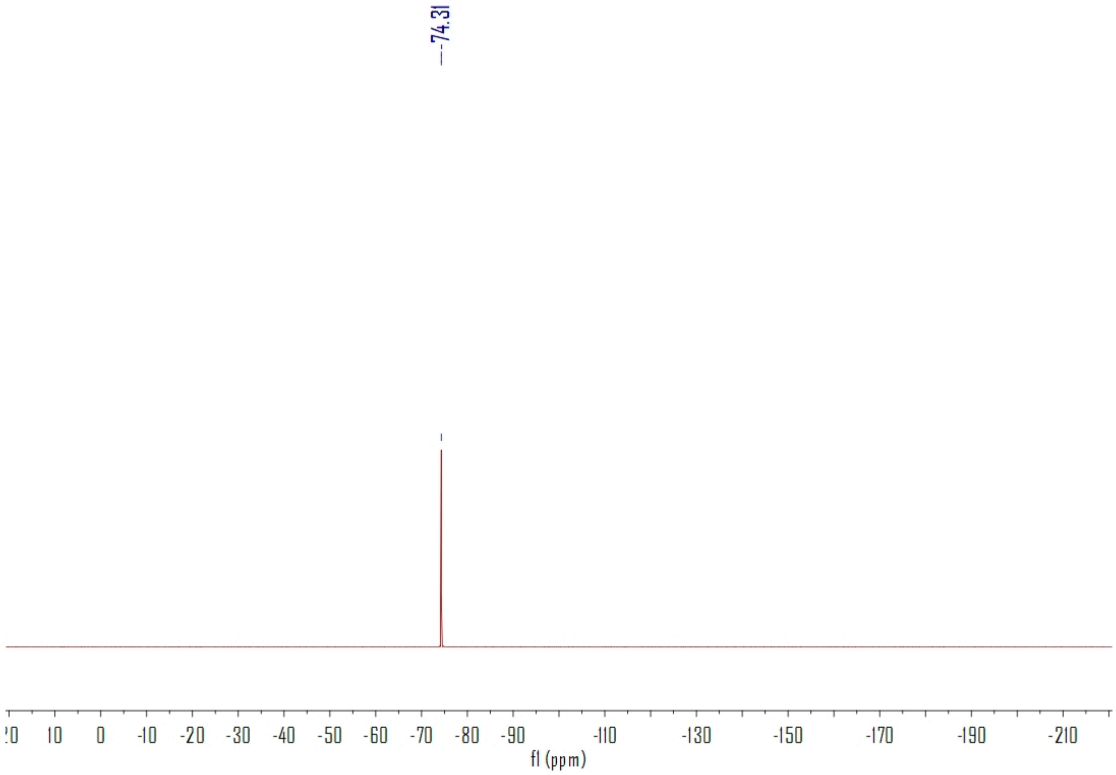

Figure S17. ^{1}H NMR spectrum of the compound (**3fa**)

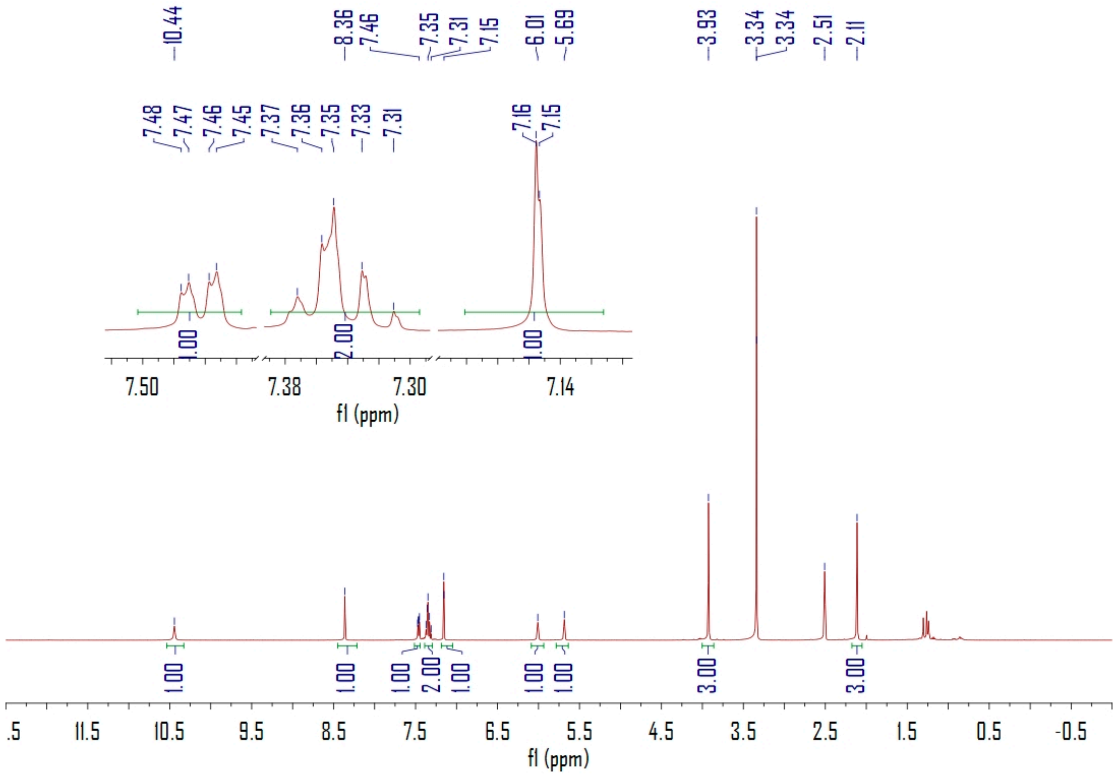

Figure S18. ^{13}C NMR spectrum of the compound (**3fa**)

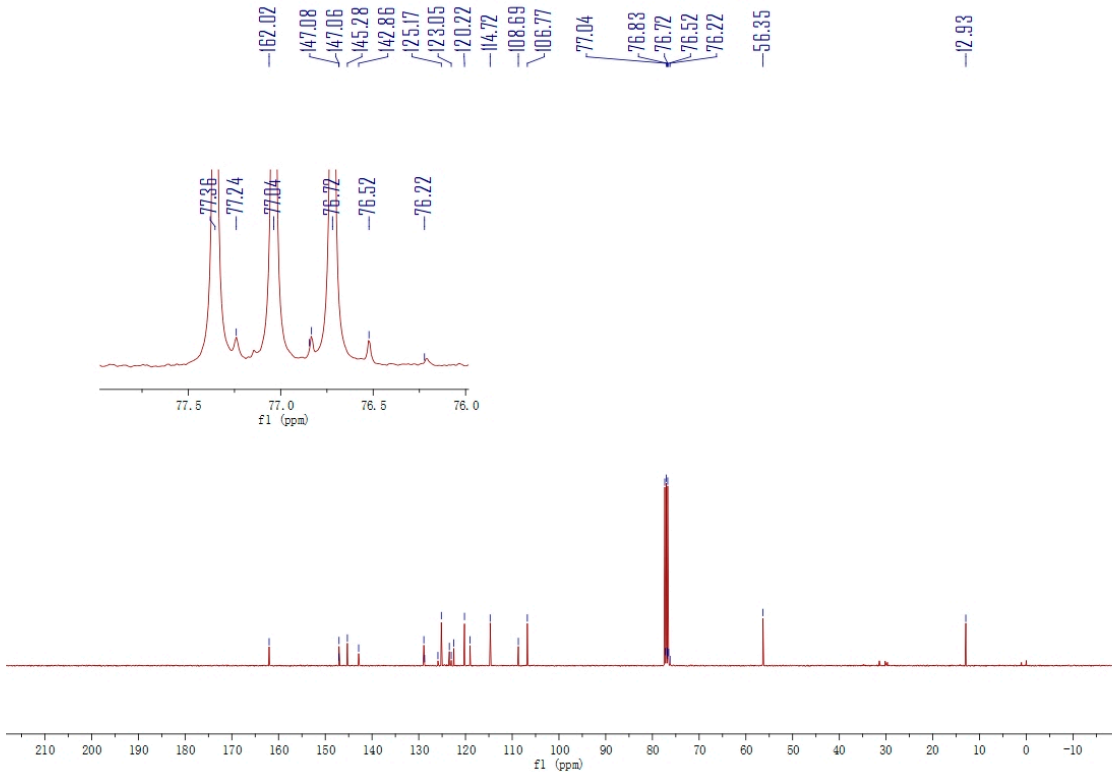

Figure S19. ^{19}F NMR spectrum of the compound (**3fa**)

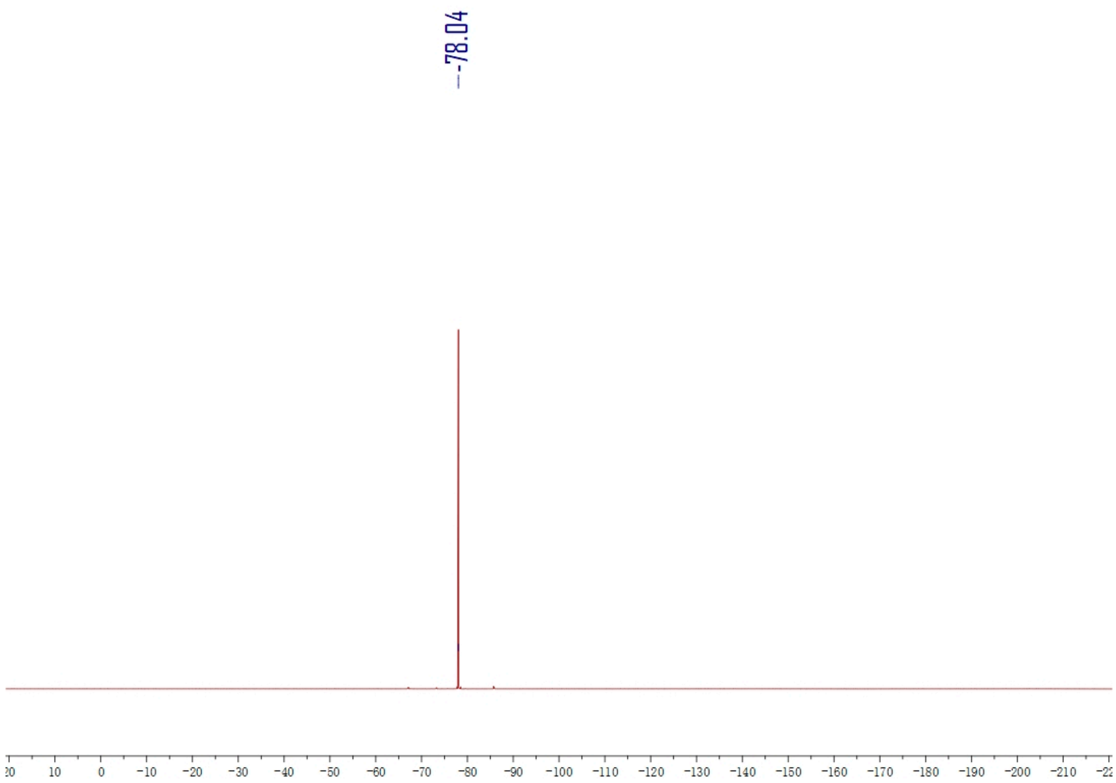

Figure S20. ^{1}H NMR spectrum of the compound (**3ga**)

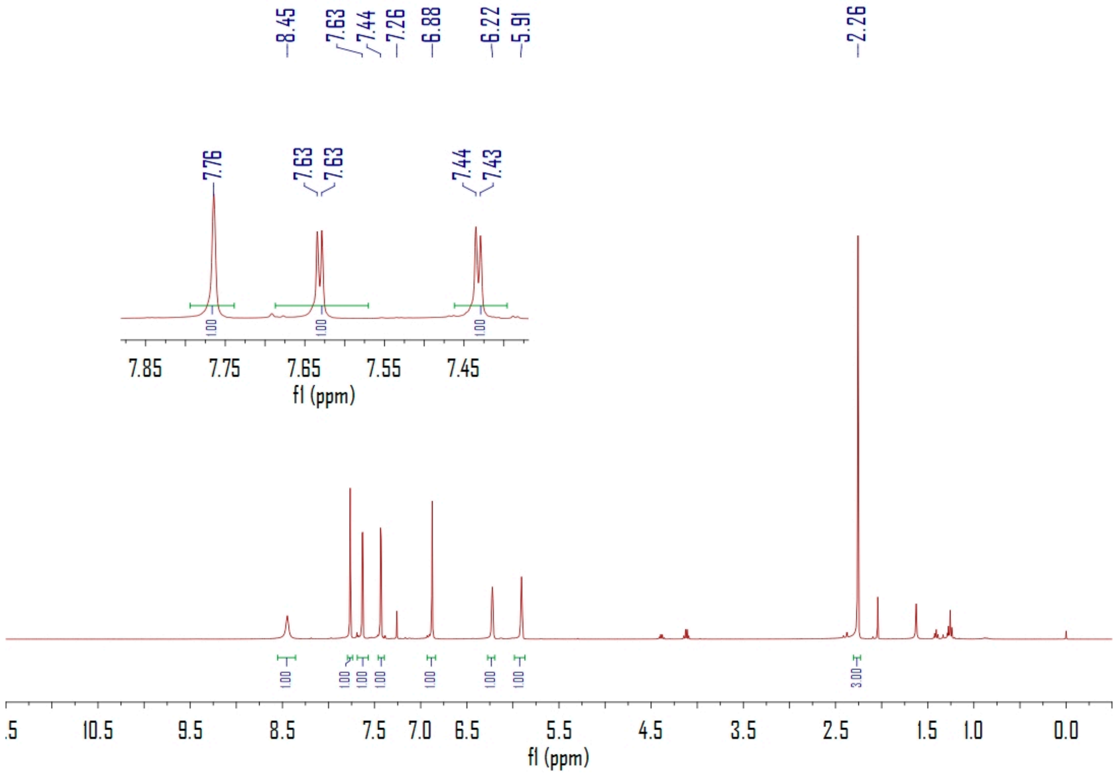

Figure S21. ^{13}C NMR spectrum of the compound (**3ga**)

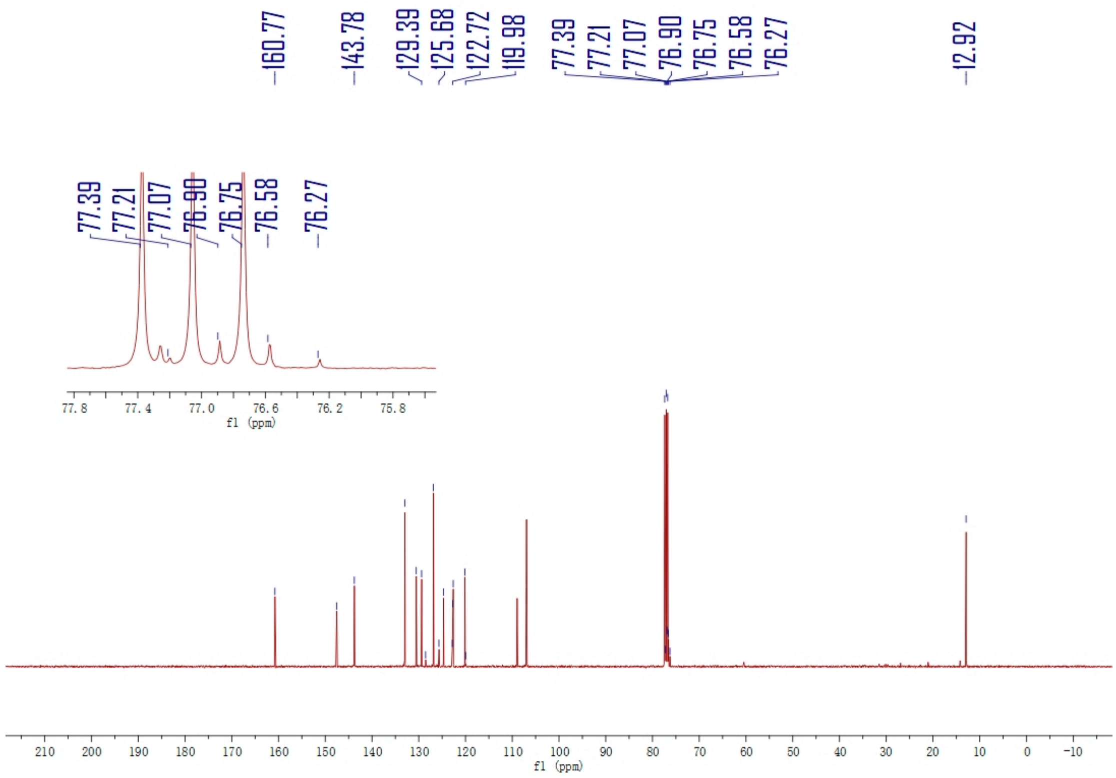

Figure S22. ^{19}F NMR spectrum of the compound (**3ga**)

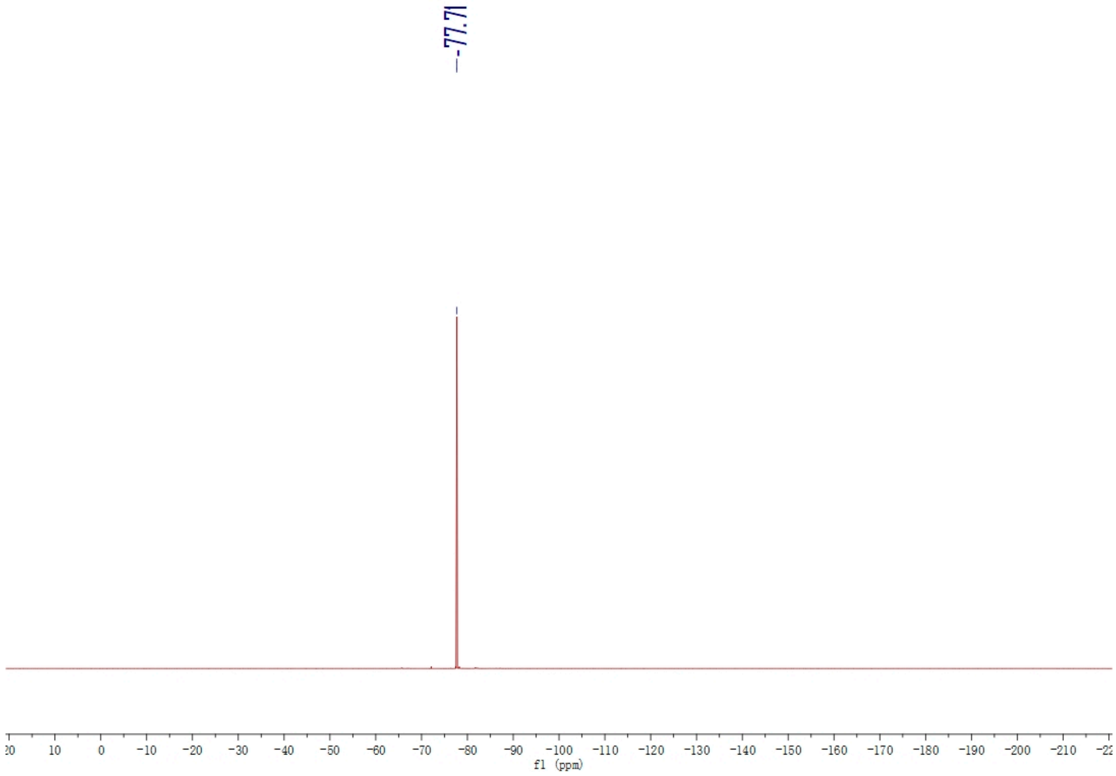

Figure S23. ^{1}H NMR spectrum of the compound (**3ha**)

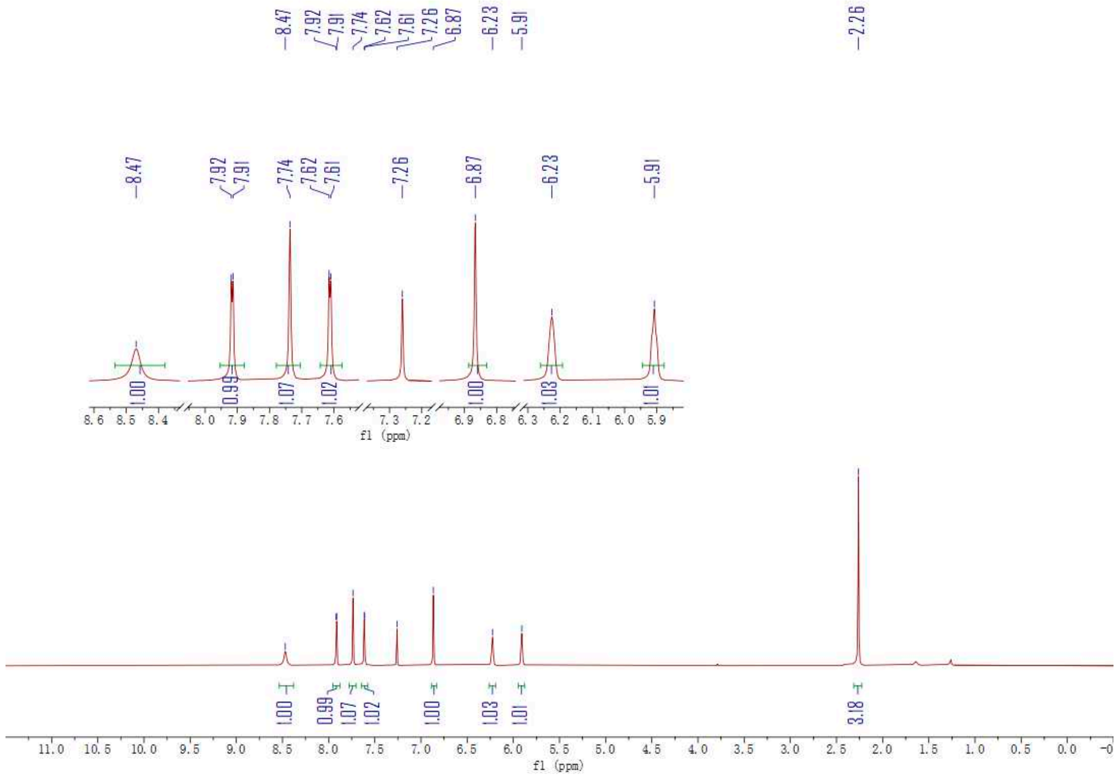

Figure S24. ^{13}C NMR spectrum of the compound (**3ha**)

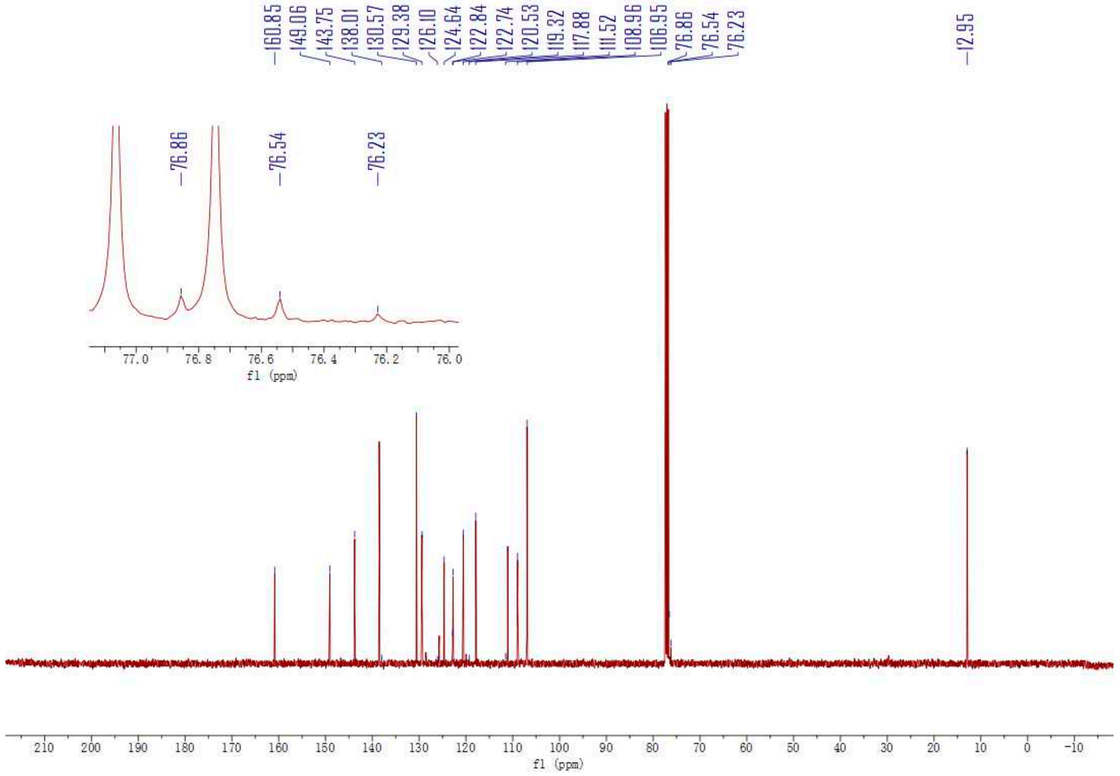

Figure S25. ^{19}F NMR spectrum of the compound (**3ha**)

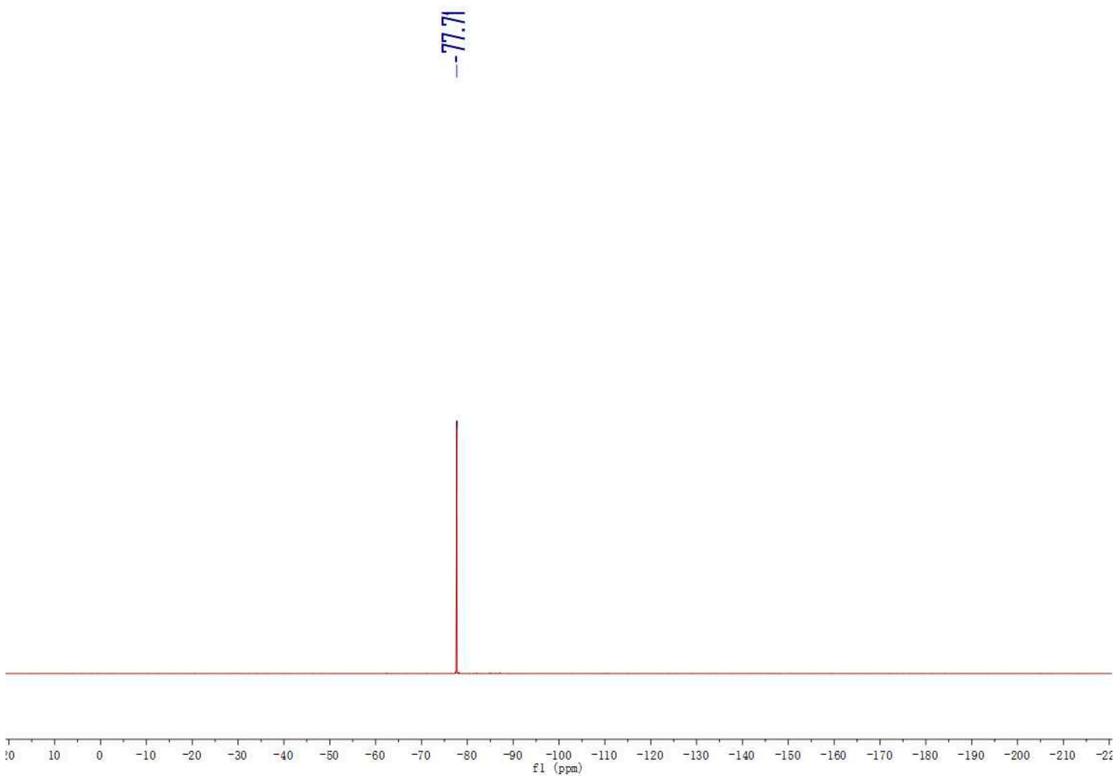

Figure S26. ^{1}H NMR spectrum of the compound (**3ia**)

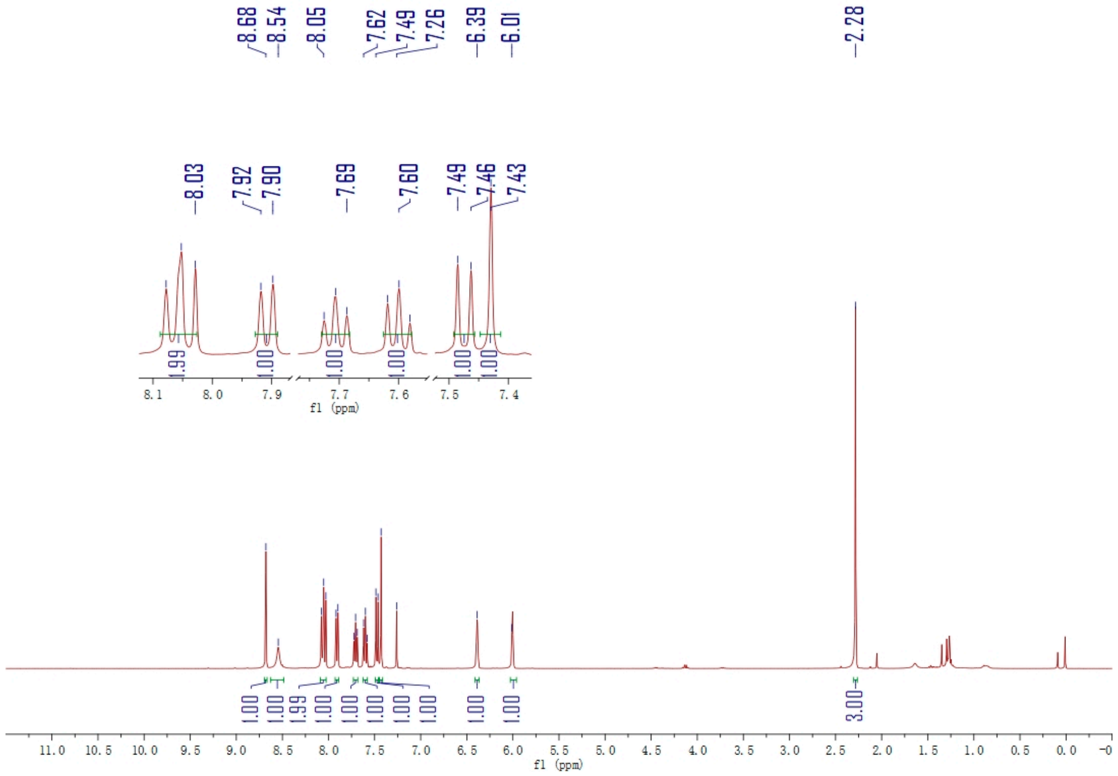

Figure S27. ^{13}C NMR spectrum of the compound (**3ia**)

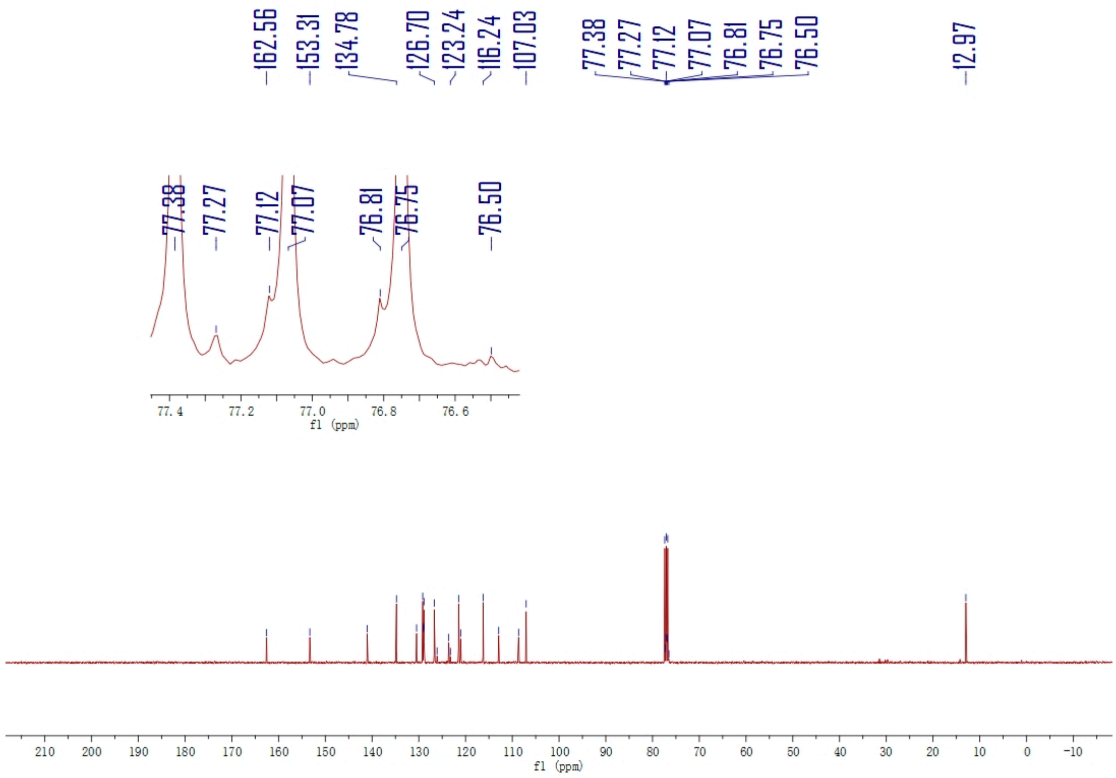

Figure S28. ^{19}F NMR spectrum of the compound (**3ia**)

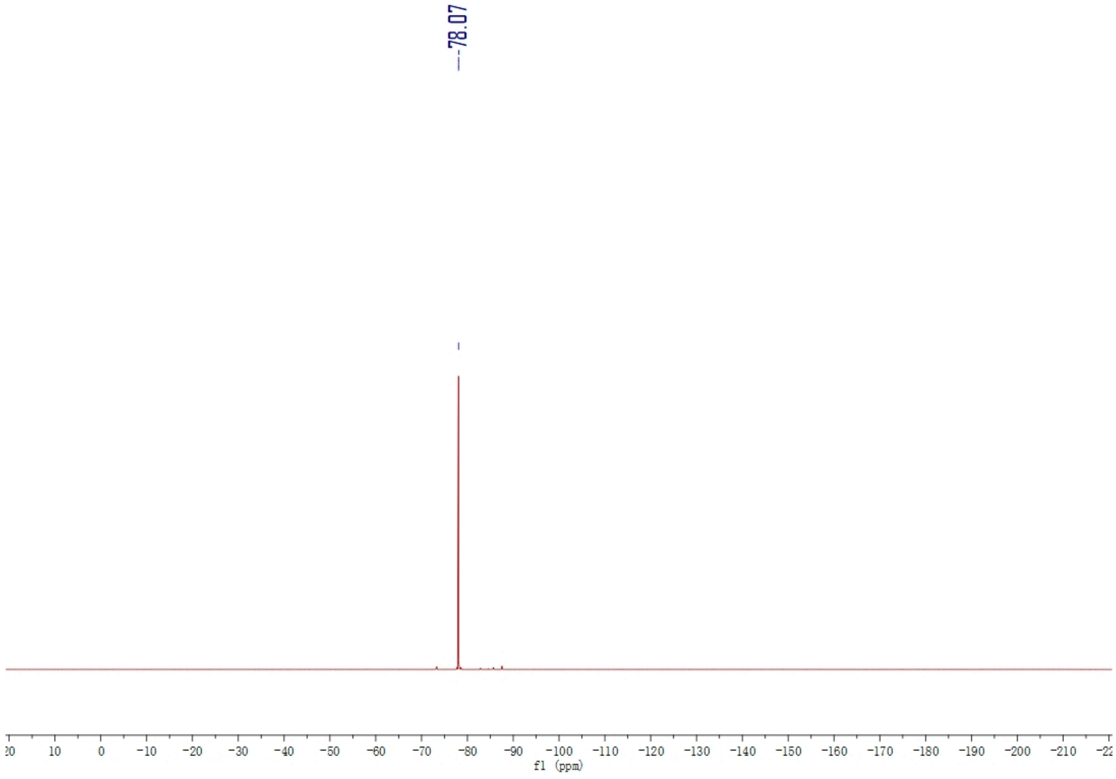

Figure S29. ^{1}H NMR spectrum of the compound (**3ab**)

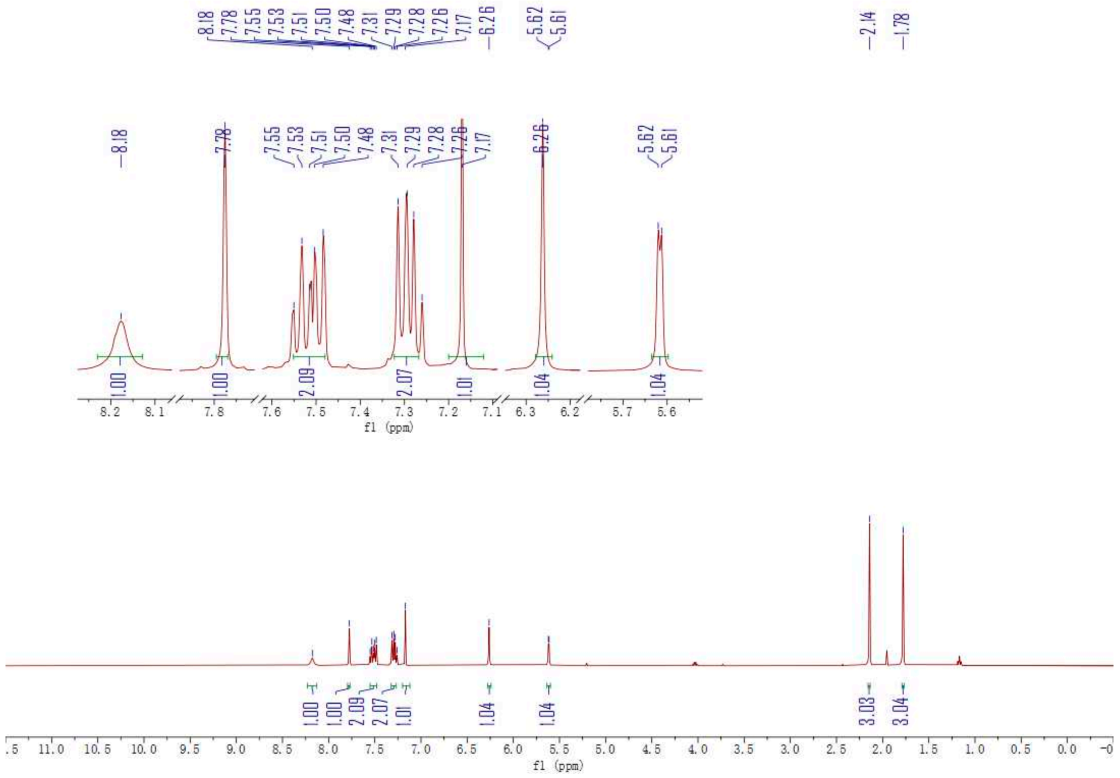

Figure S30. ^{13}C NMR spectrum of the compound (**3ab**)

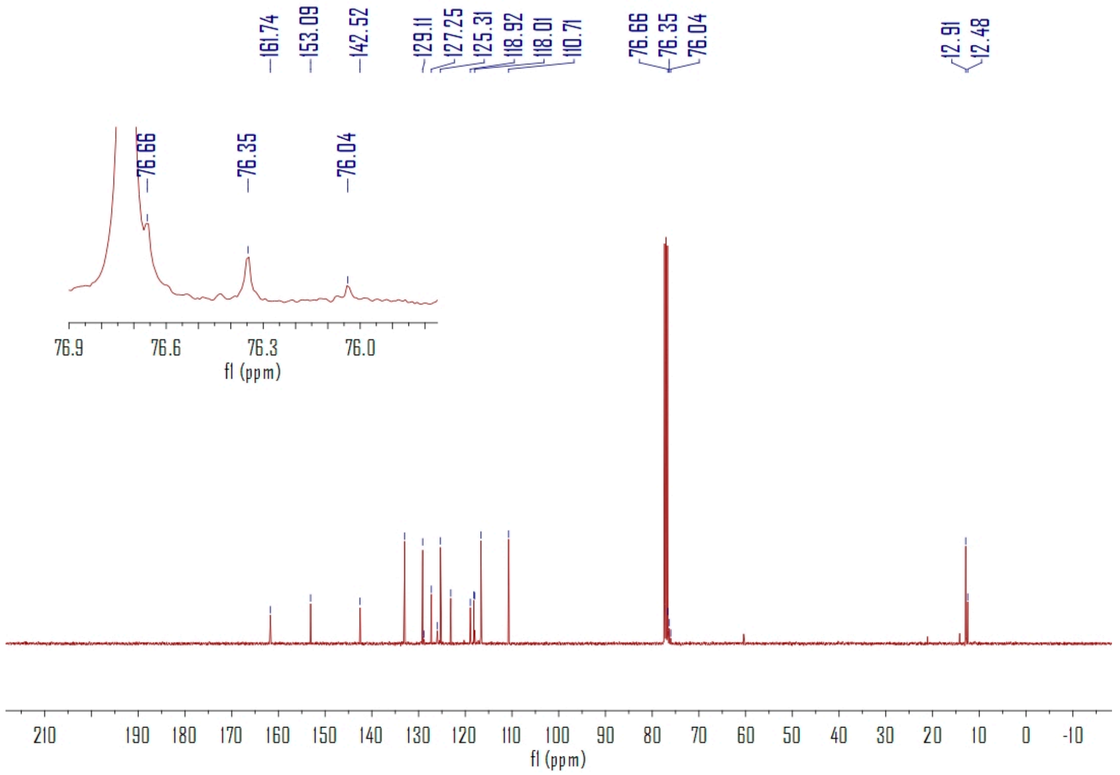

Figure S31. ^{19}F NMR spectrum of the compound (**3ab**)

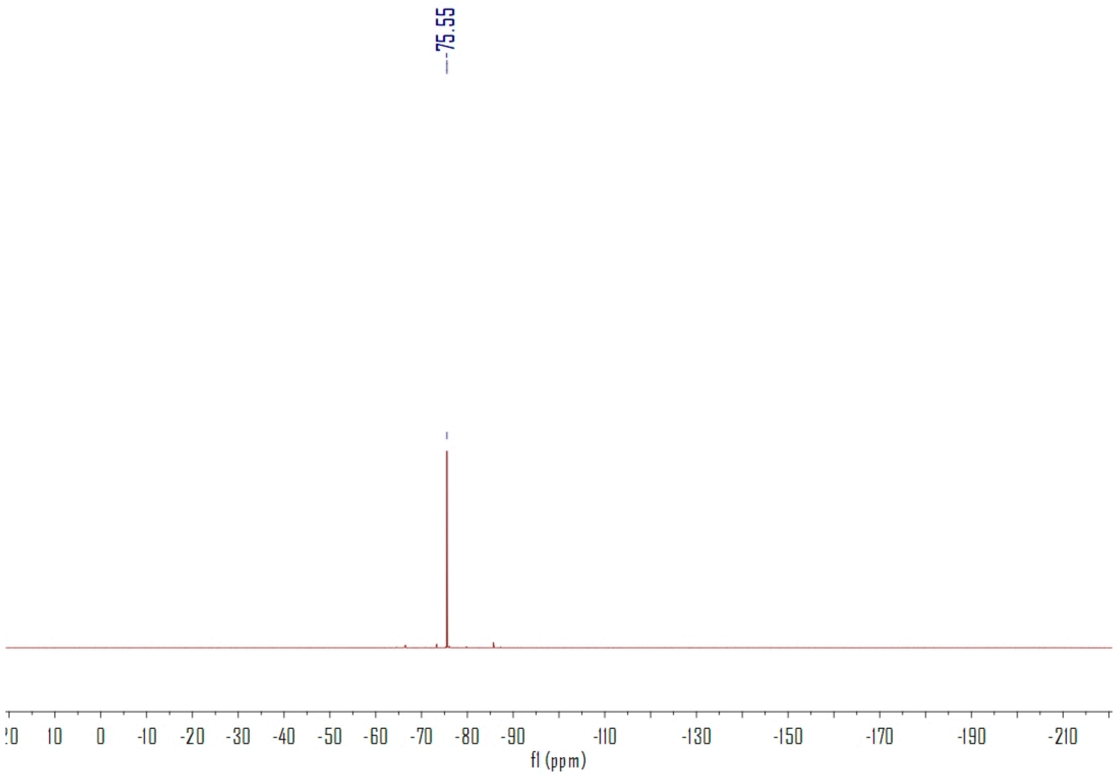

Figure S32. ^{1}H NMR spectrum of the compound (**3fb**)

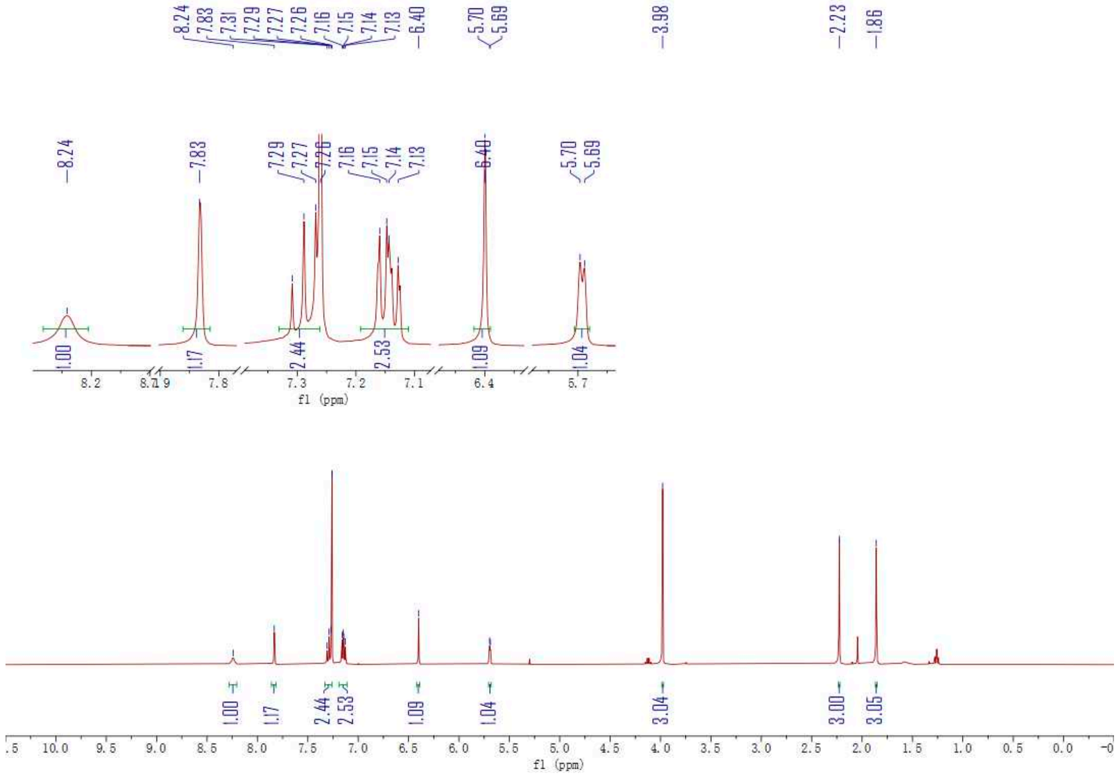

Figure S33. ^{13}C NMR spectrum of the compound (**3fb**)

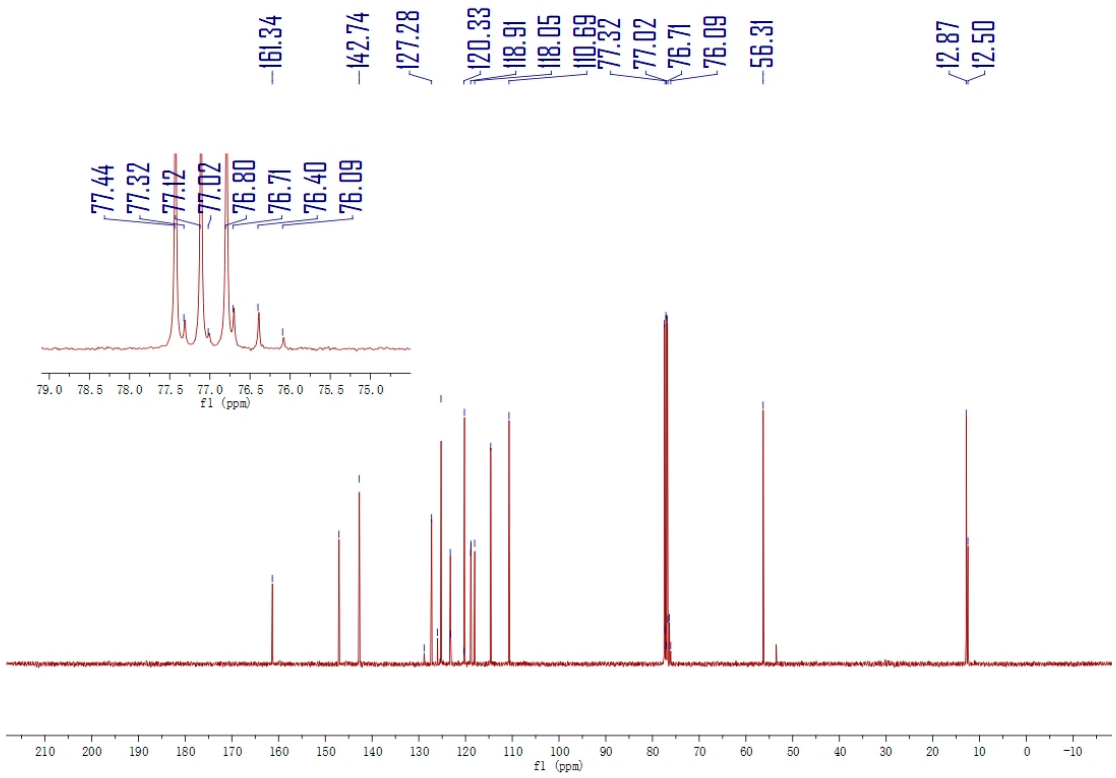

Figure S34. ^{19}F NMR spectrum of the compound (**3fb**)

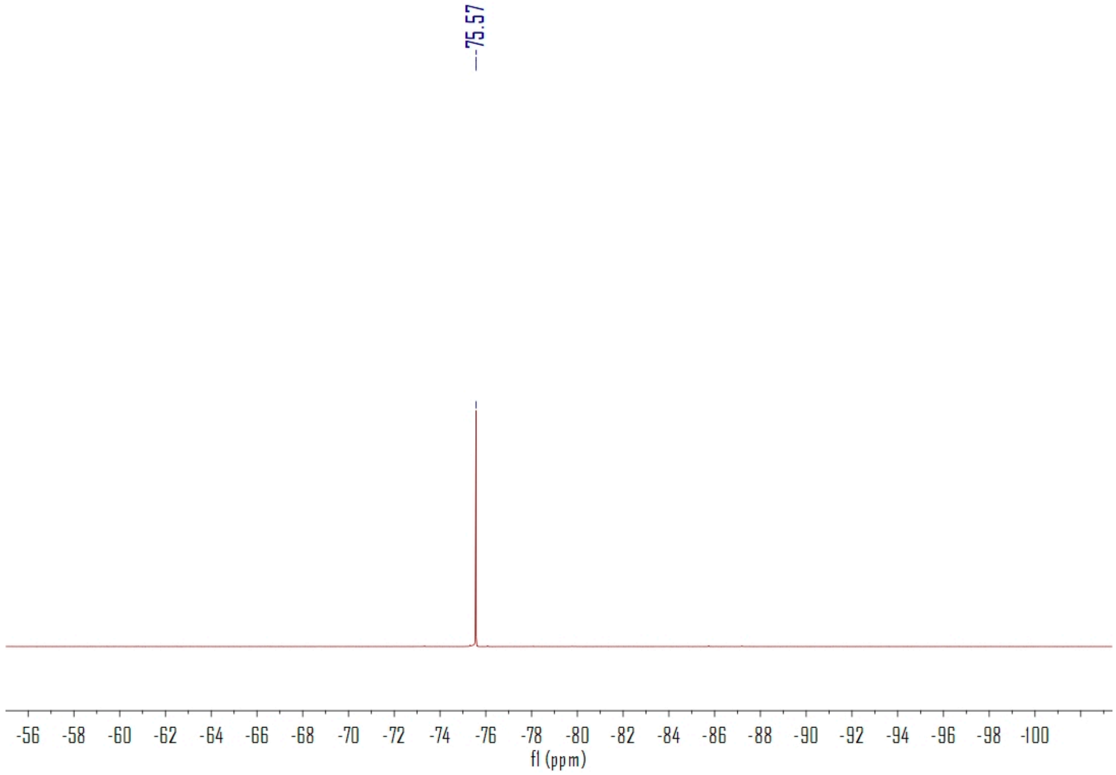

Figure S35. ^{1}H NMR spectrum of the compound (**3ac**)

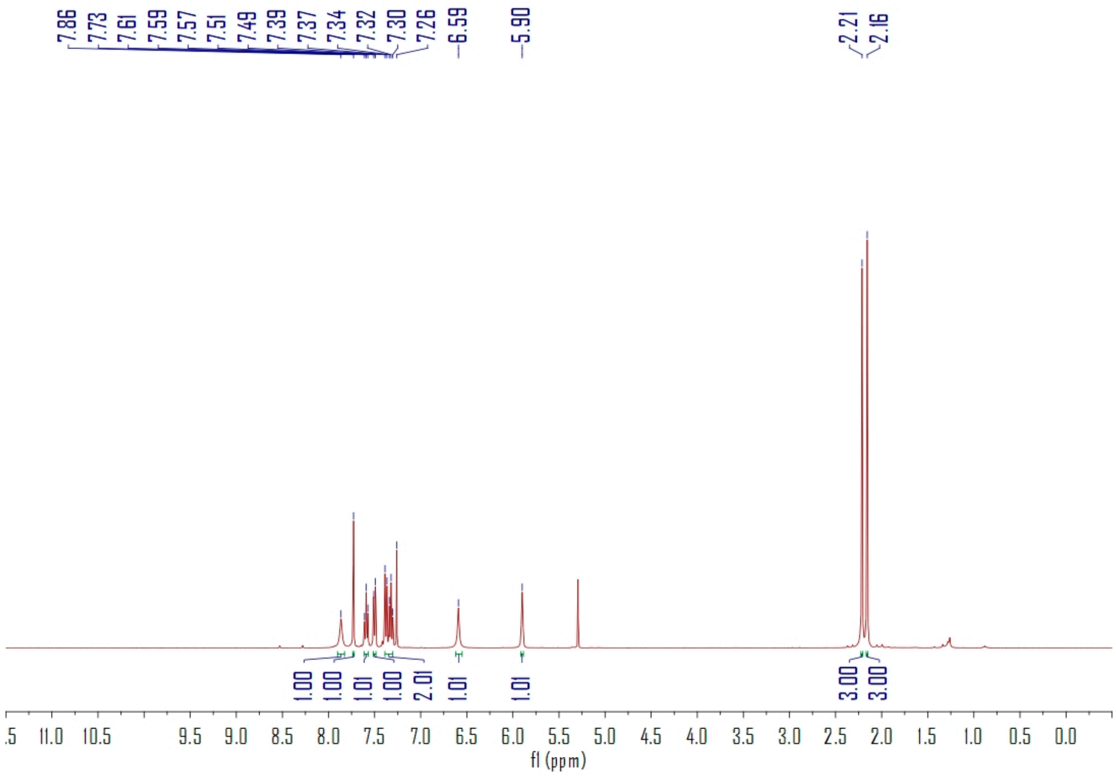

Figure S36. ^{13}C NMR spectrum of the compound (**3ac**)

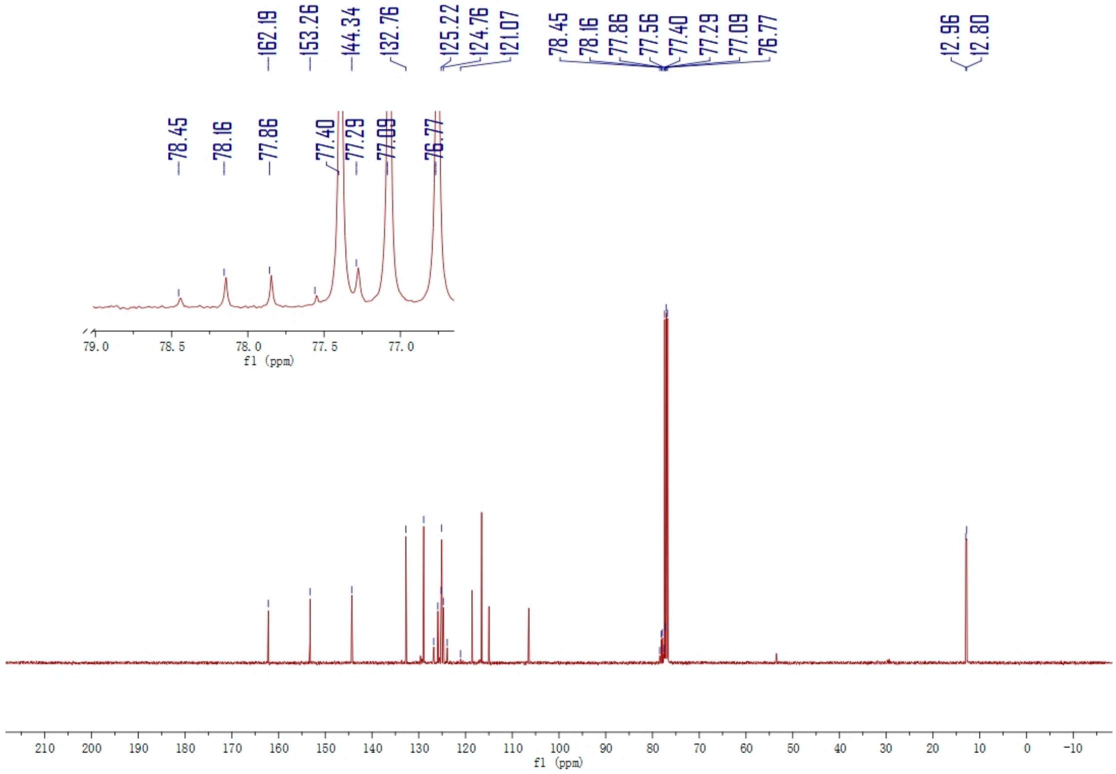

Figure S37. ^{19}F NMR spectrum of the compound (**3ac**)

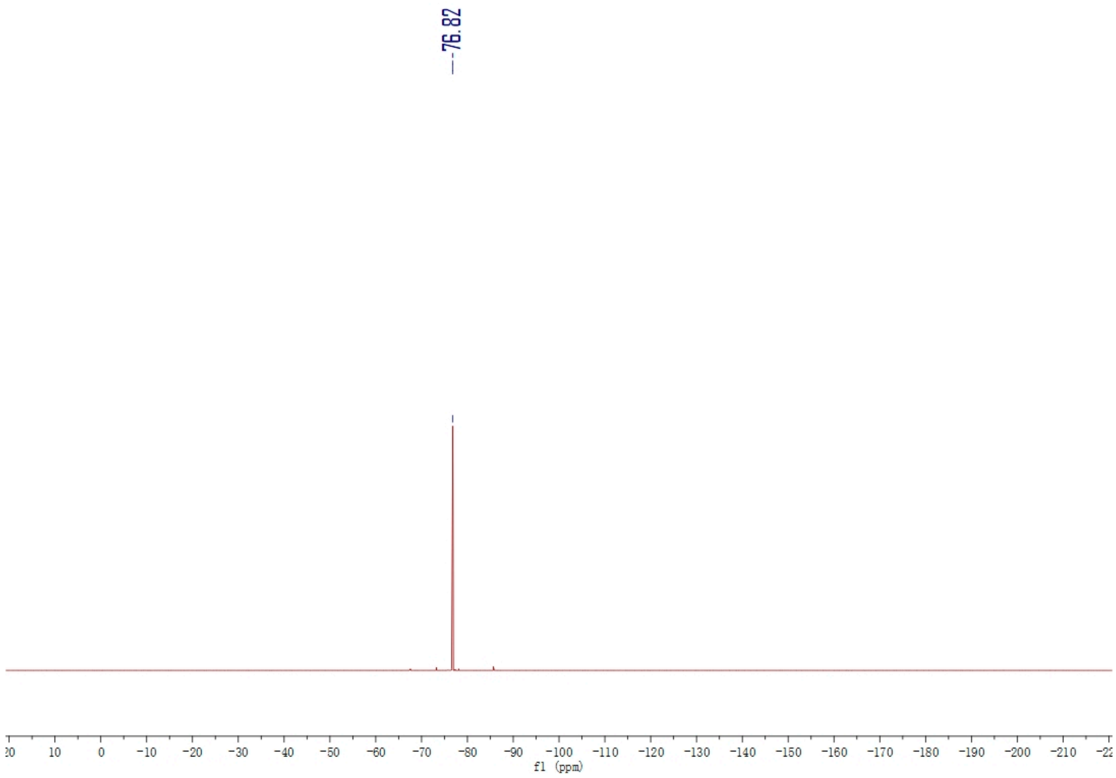

Figure S38. ^{1}H NMR spectrum of the compound (**3bc**)

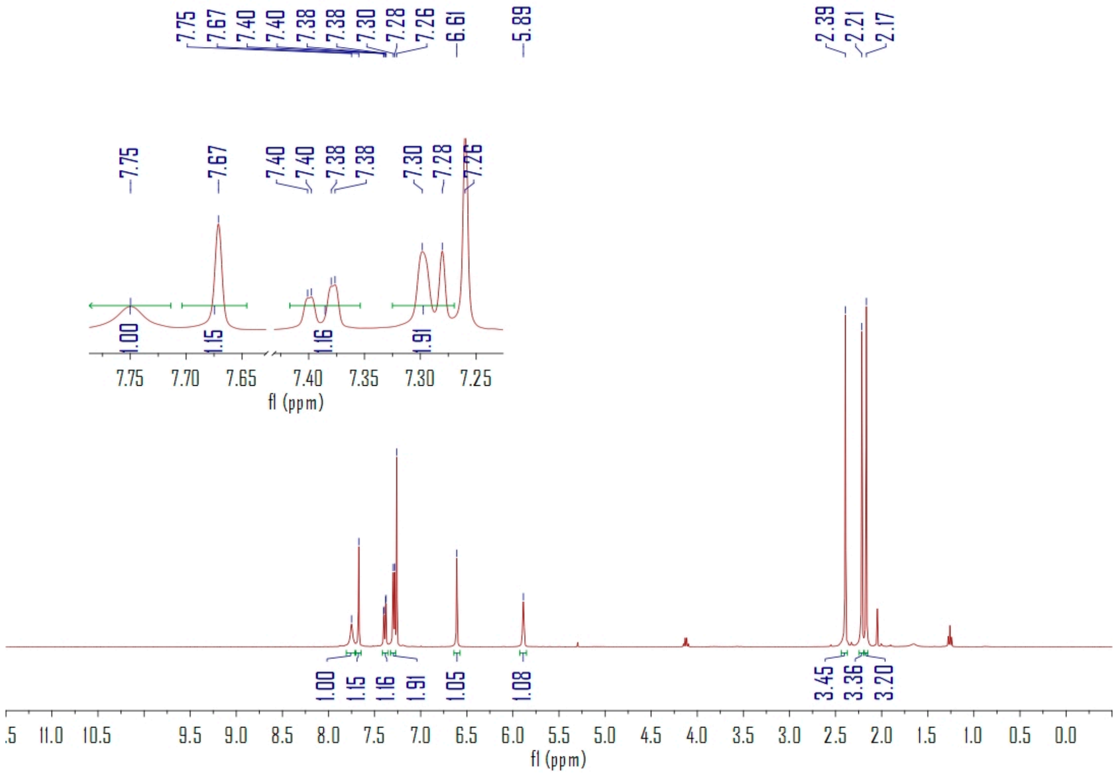

Figure S39. ^{13}C NMR spectrum of the compound (**3bc**)

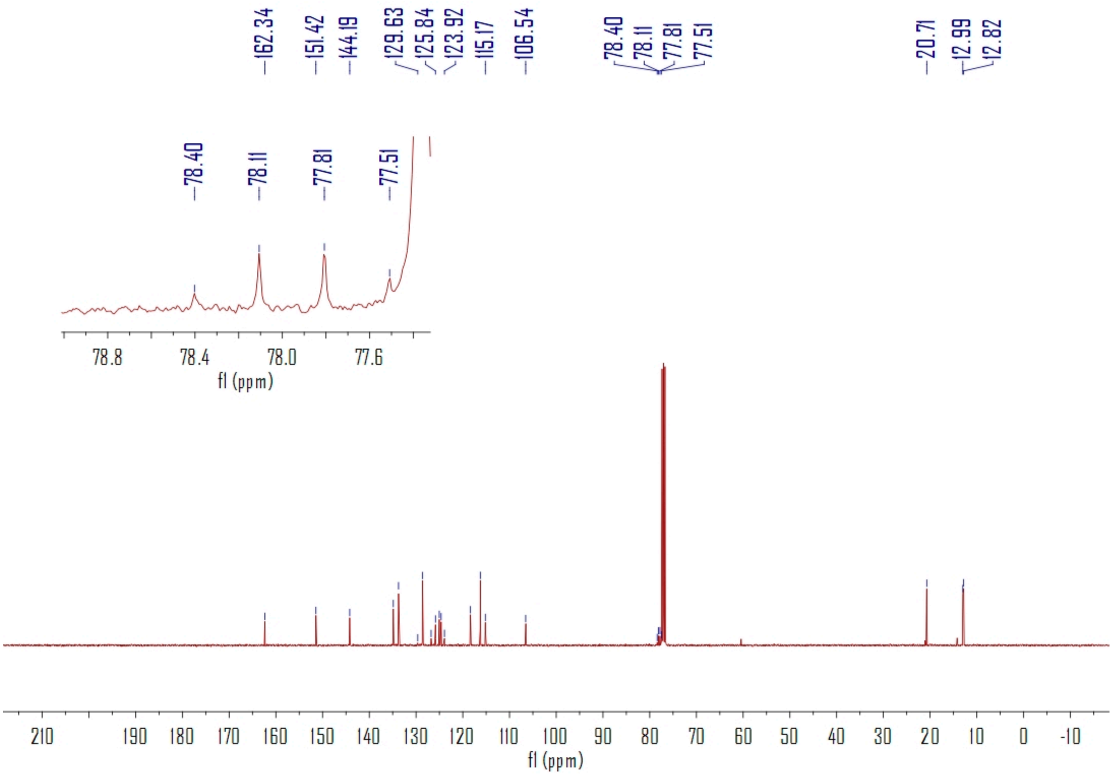

Figure S40. ^{19}F NMR spectrum of the compound (**3bc**)

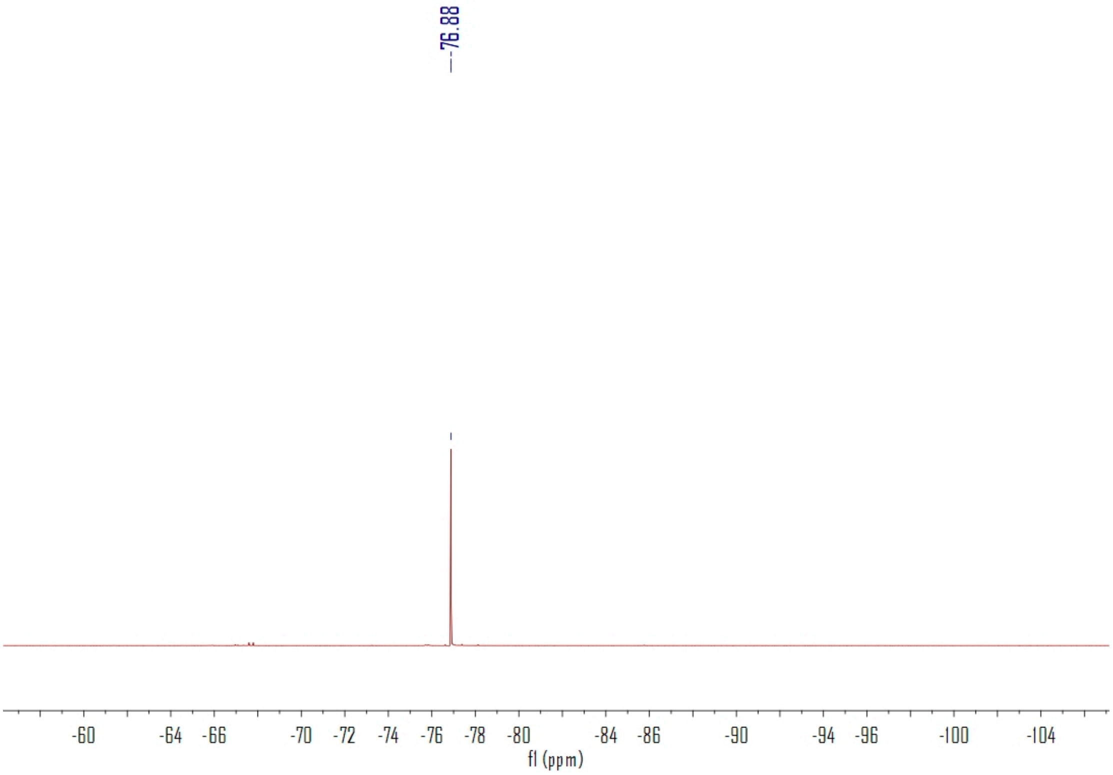

Figure S41. ^{1}H NMR spectrum of the compound (**3cc**)

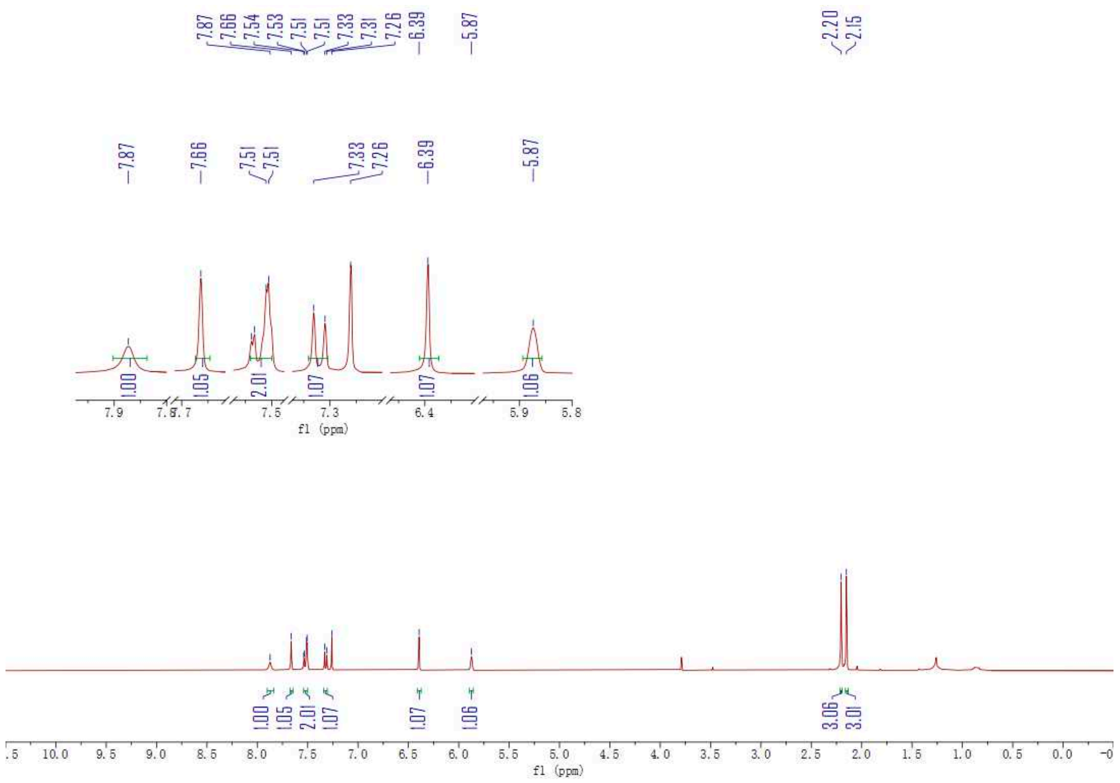

Figure S42. ^{13}C NMR spectrum of the compound (**3cc**)

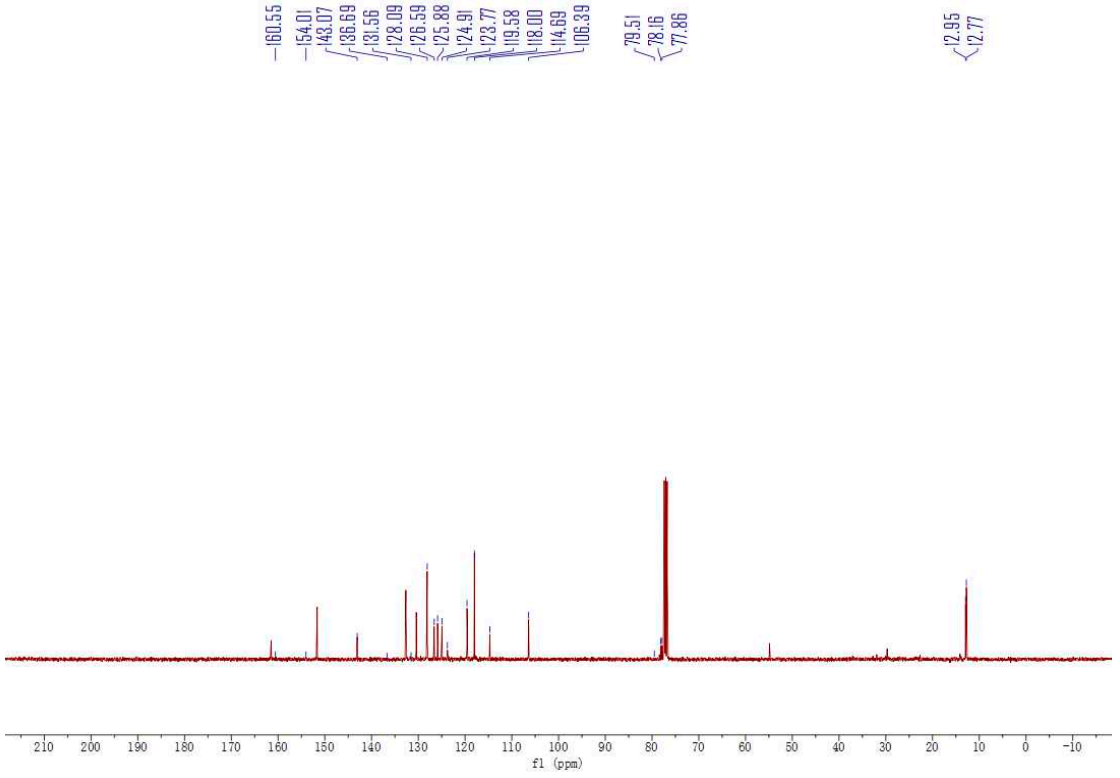

Figure S43. ^{19}F NMR spectrum of the compound (**3cc**)

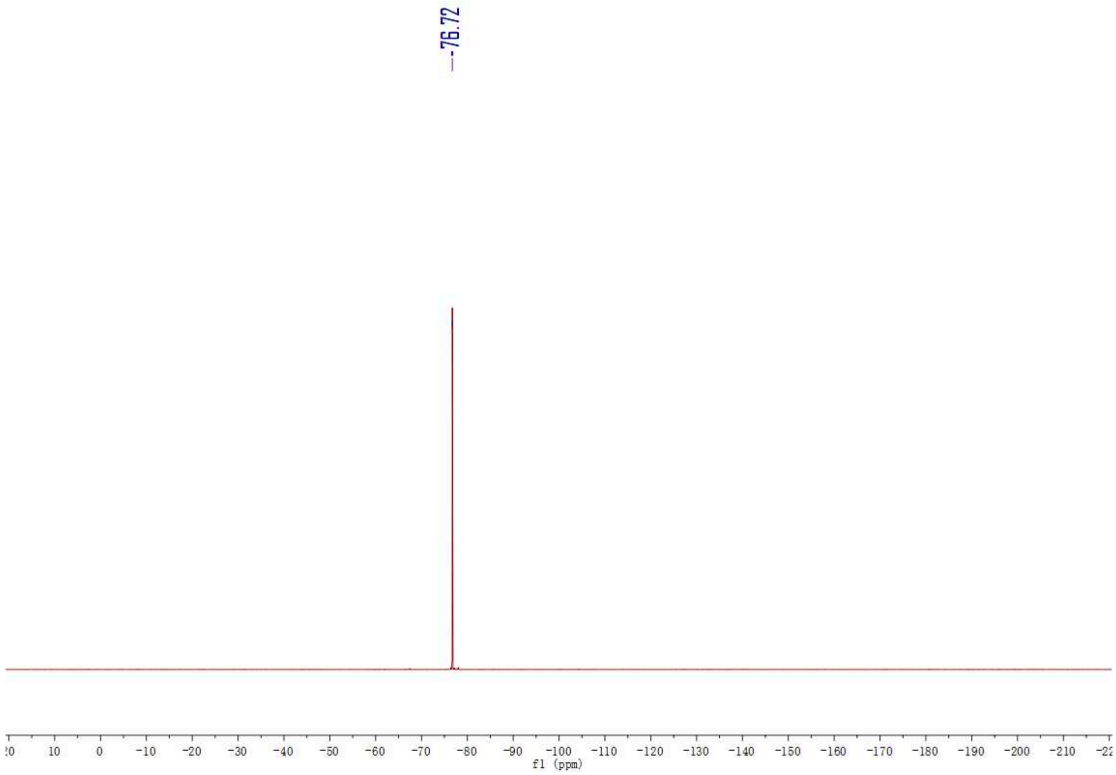

Figure S44. ^{1}H NMR spectrum of the compound (**3dc**)

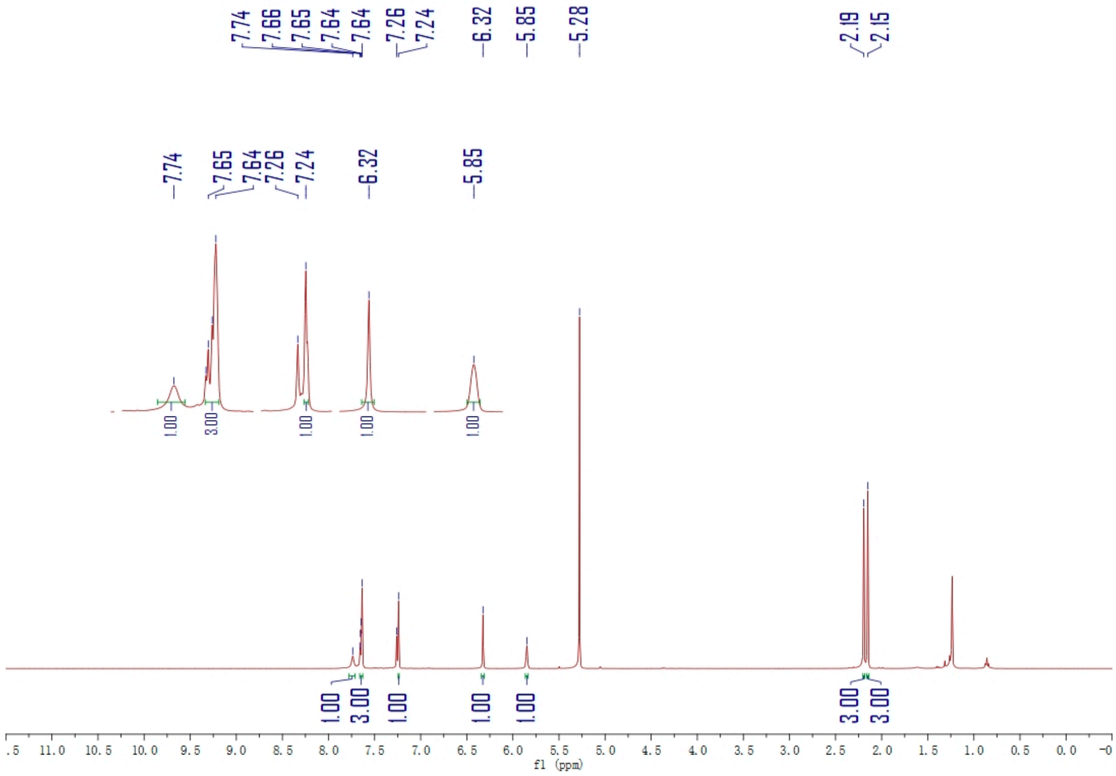

Figure S45. ^{13}C NMR spectrum of the compound (**3dc**)

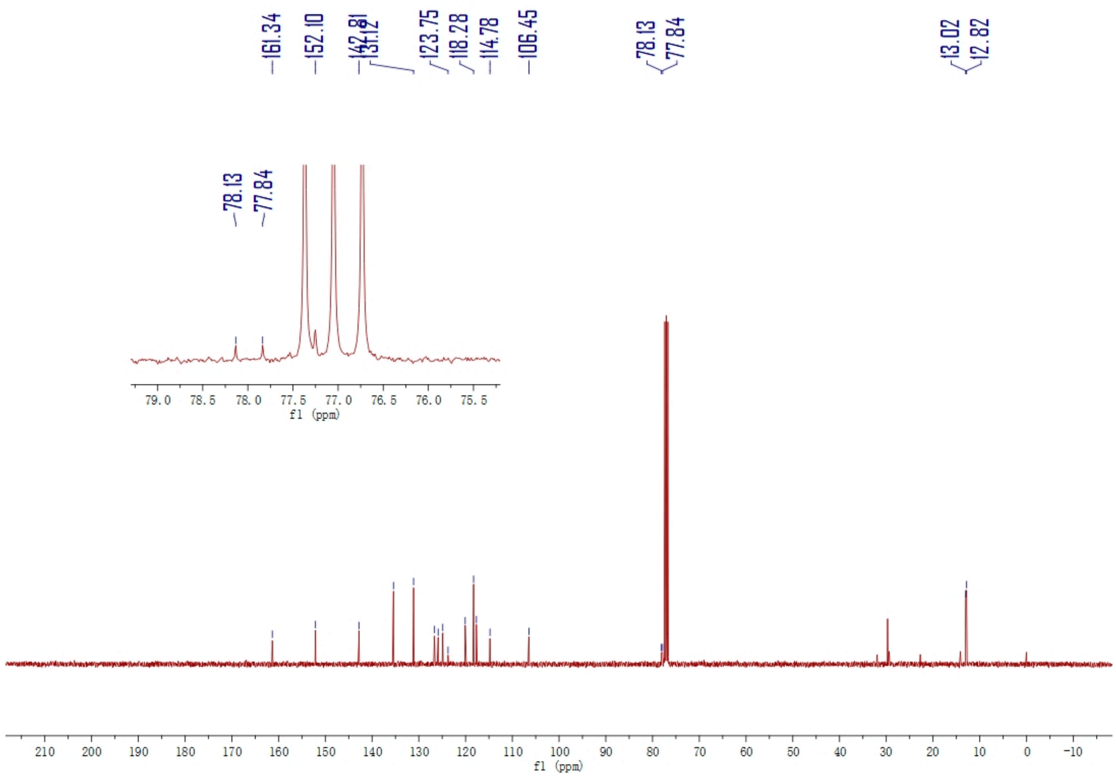

Figure S46. ^{13}C NMR spectrum of the compound (**3dc**)

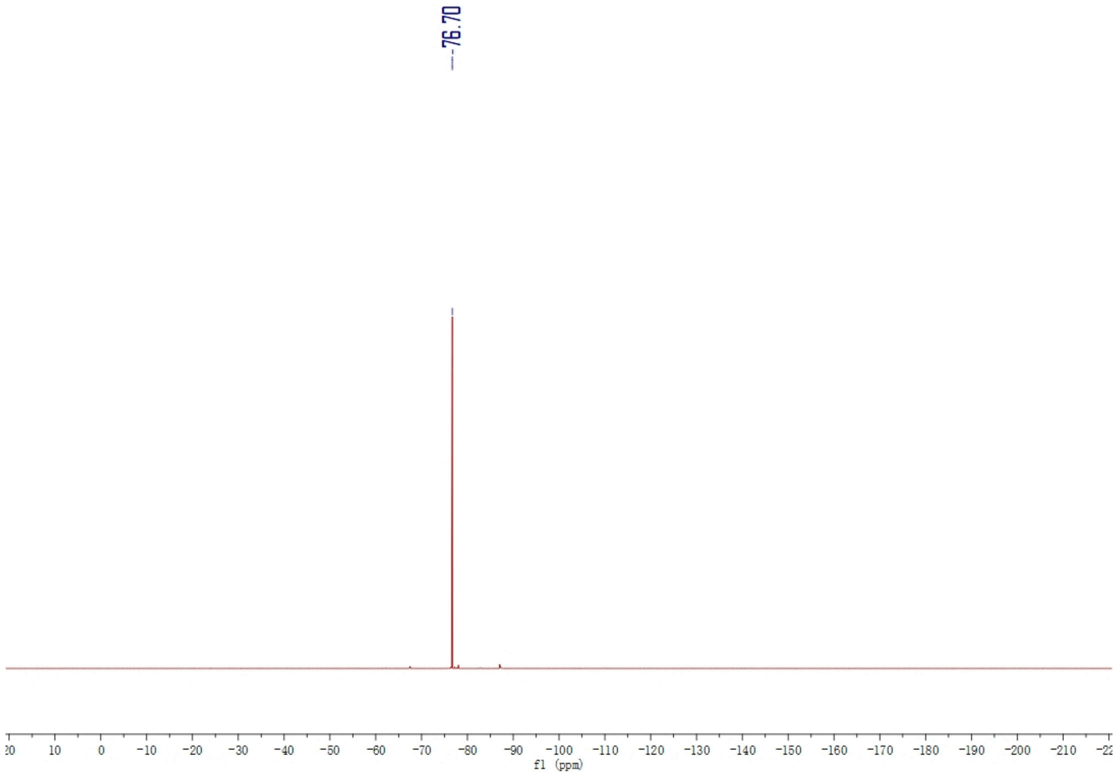

Figure S47. ^{1}H NMR spectrum of the compound (**3ec**)

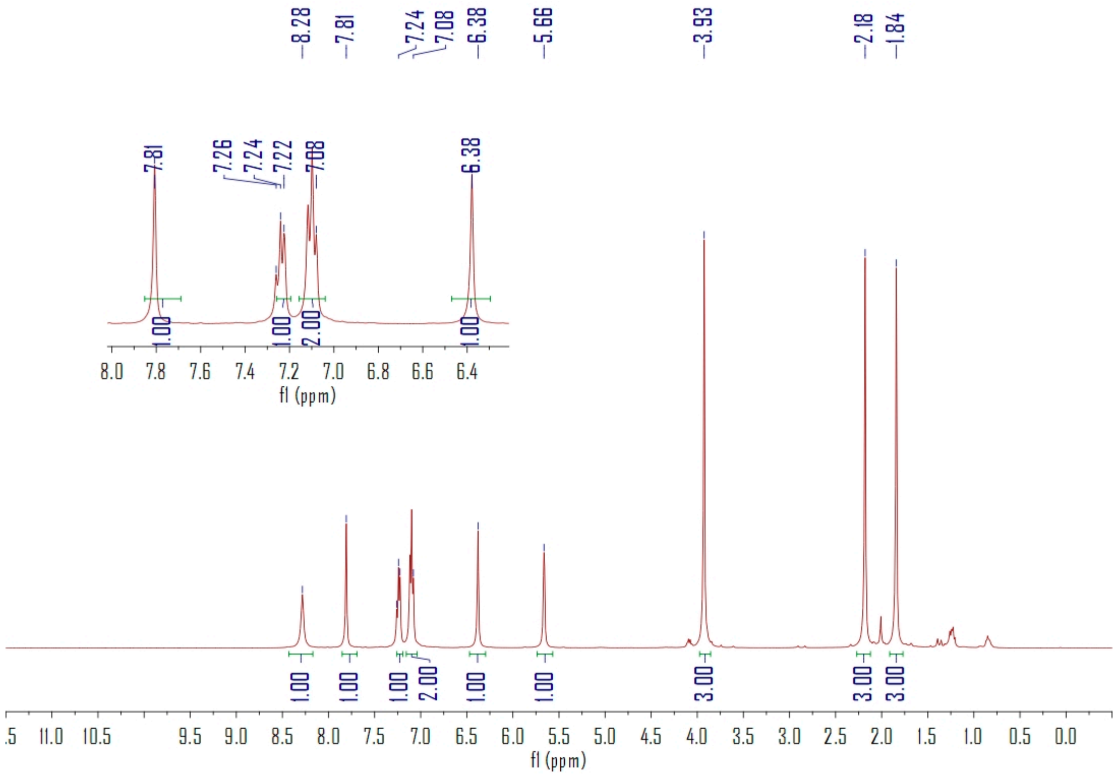

Figure S48. ^{13}C NMR spectrum of the compound (**3ec**)

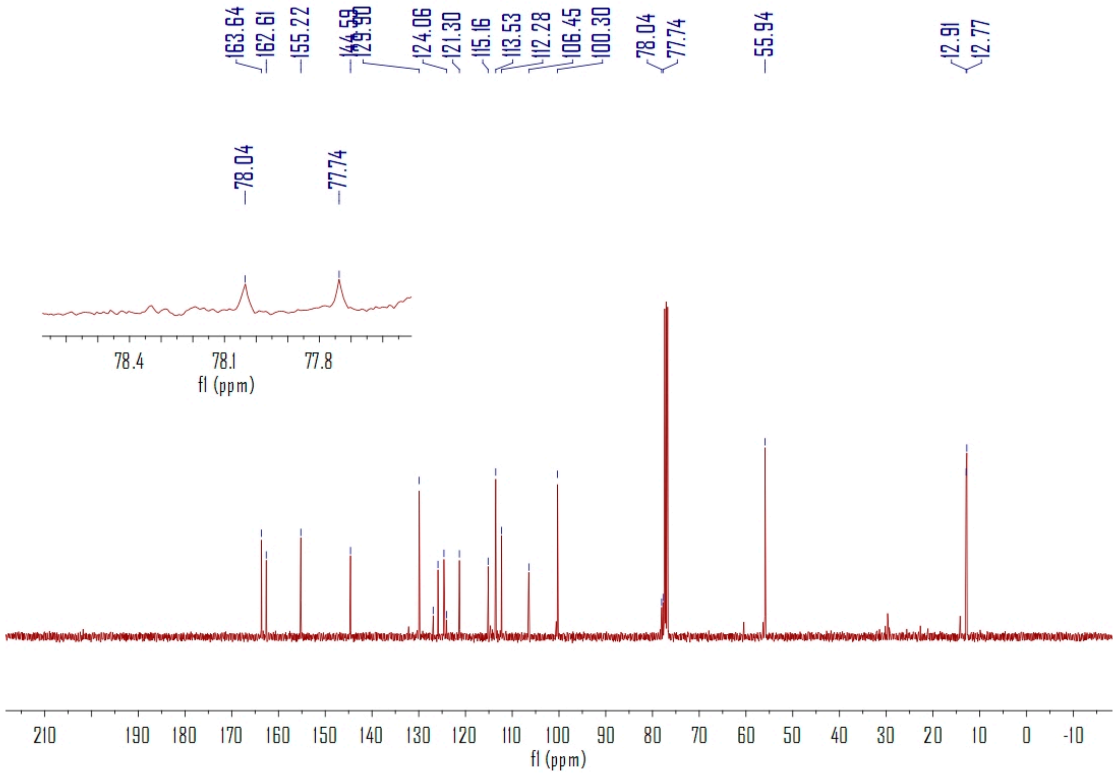

Figure S49. ^{19}F NMR spectrum of the compound (**3ec**)

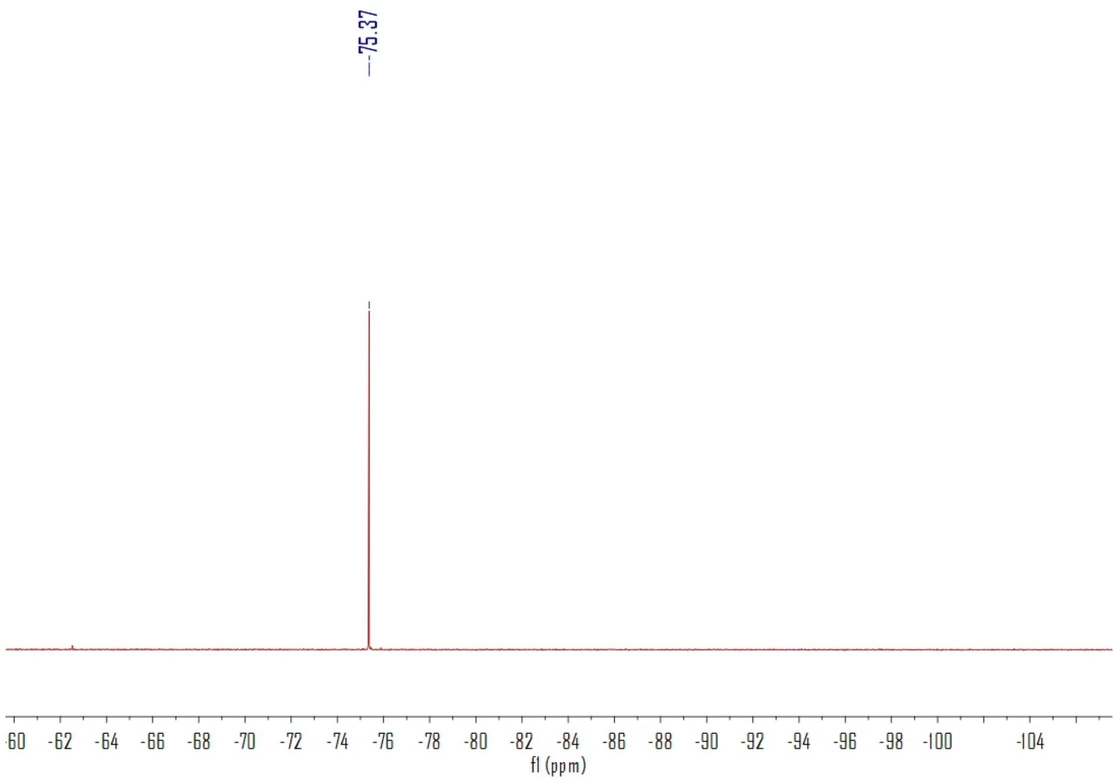

Figure S50. ^{1}H NMR spectrum of the compound (**3fc**)

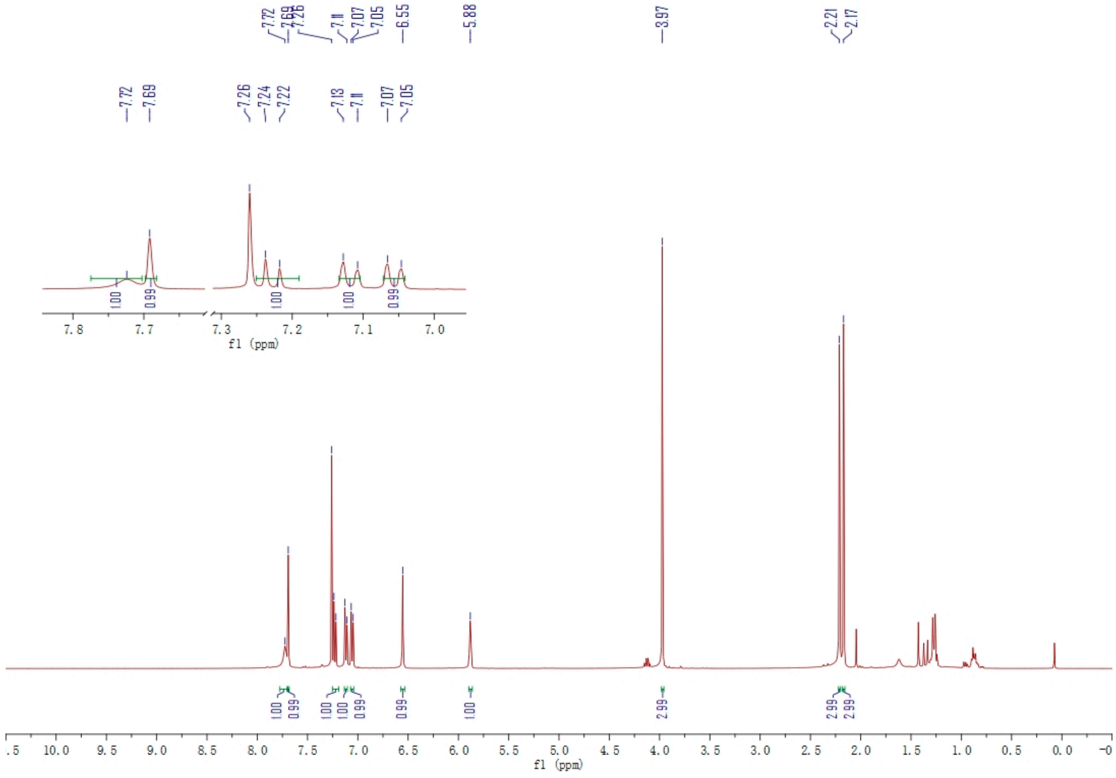

Figure S51. ^{13}C NMR spectrum of the compound (**3fc**)

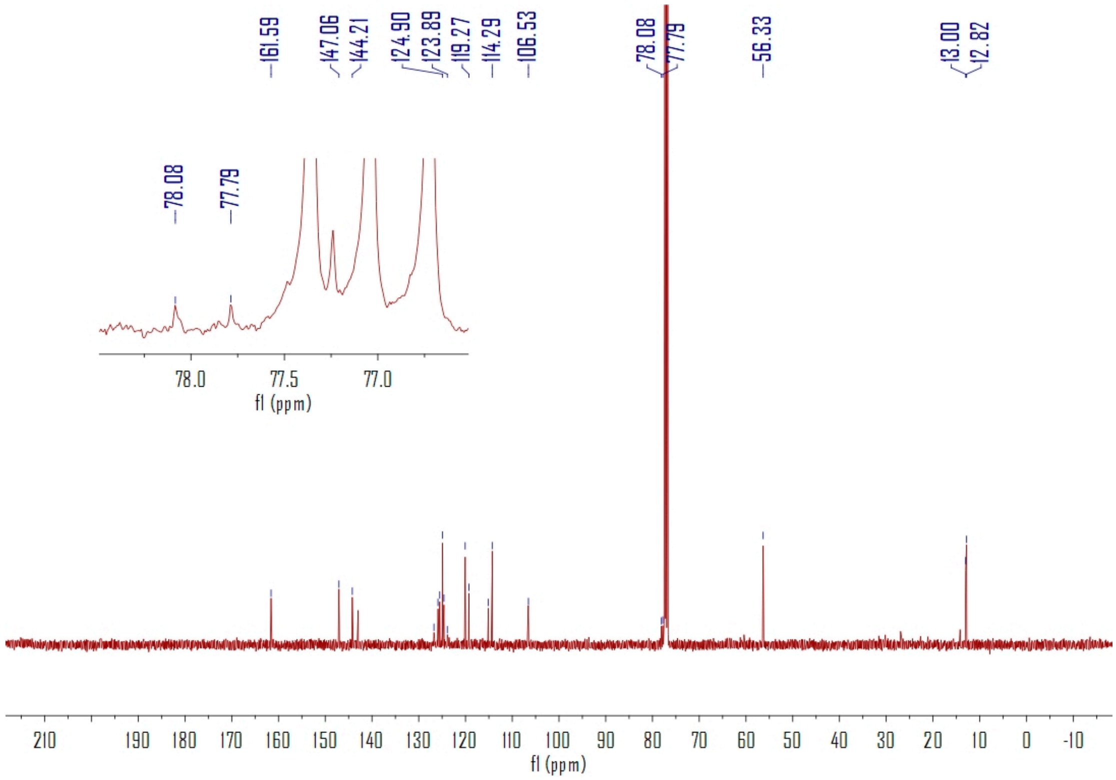

Figure S52. ^{19}F NMR spectrum of the compound (**3fc**)

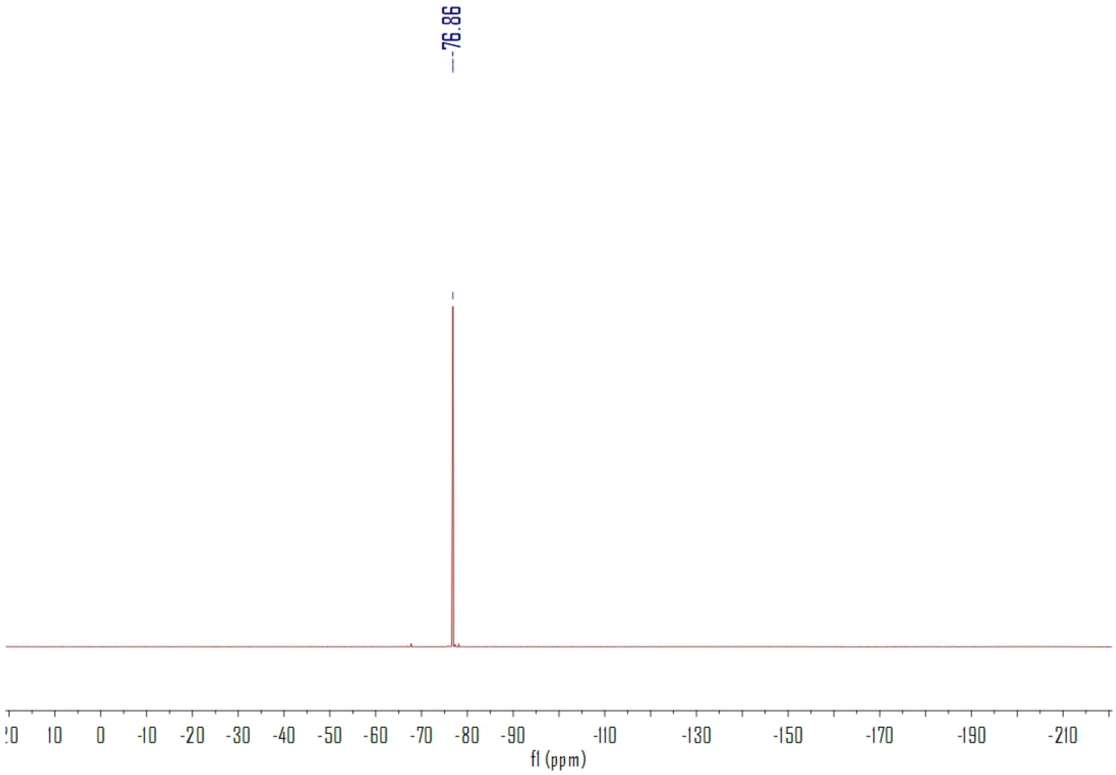

Figure S53. ^{1}H NMR spectrum of the compound (**3gc**)

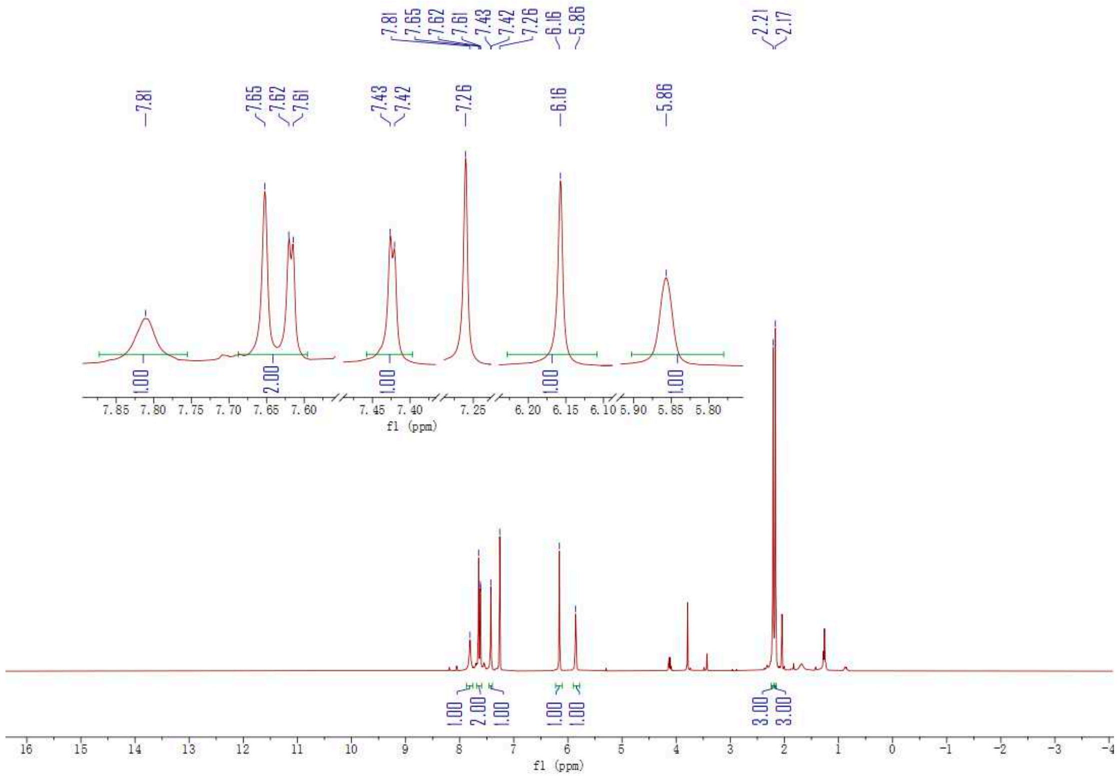

Figure S54. ^{13}C NMR spectrum of the compound (**3gc**)

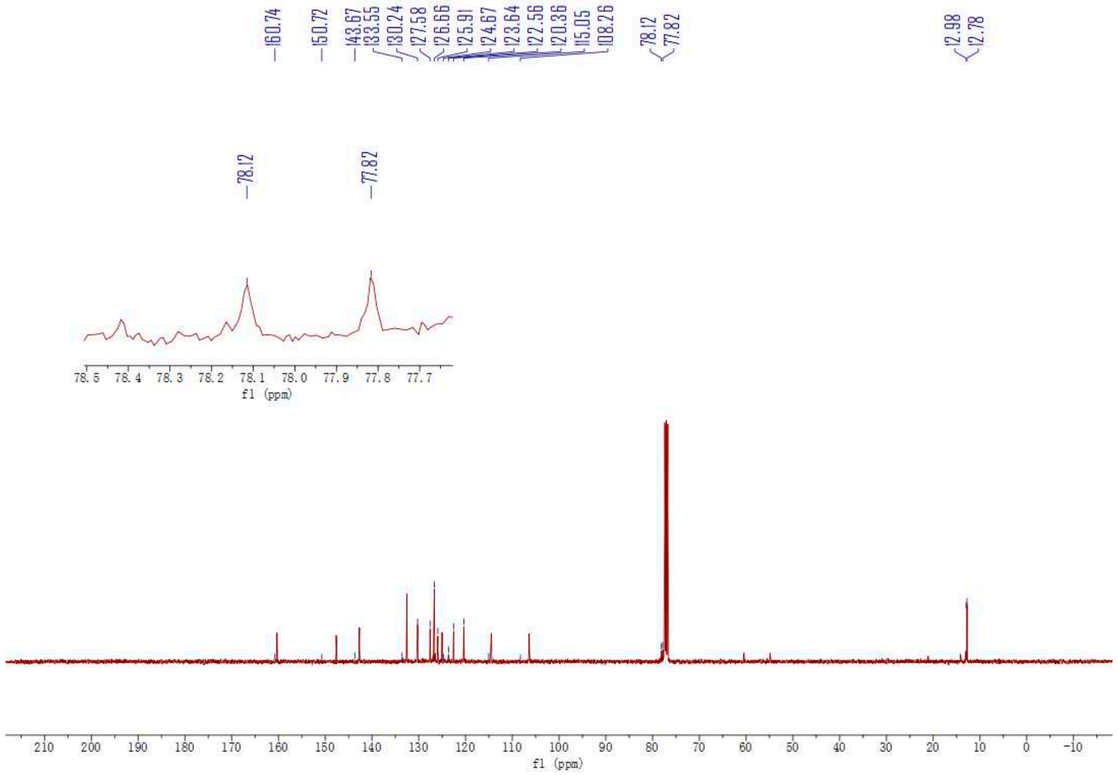

Figure S55. ^{13}C NMR spectrum of the compound (**3gc**)

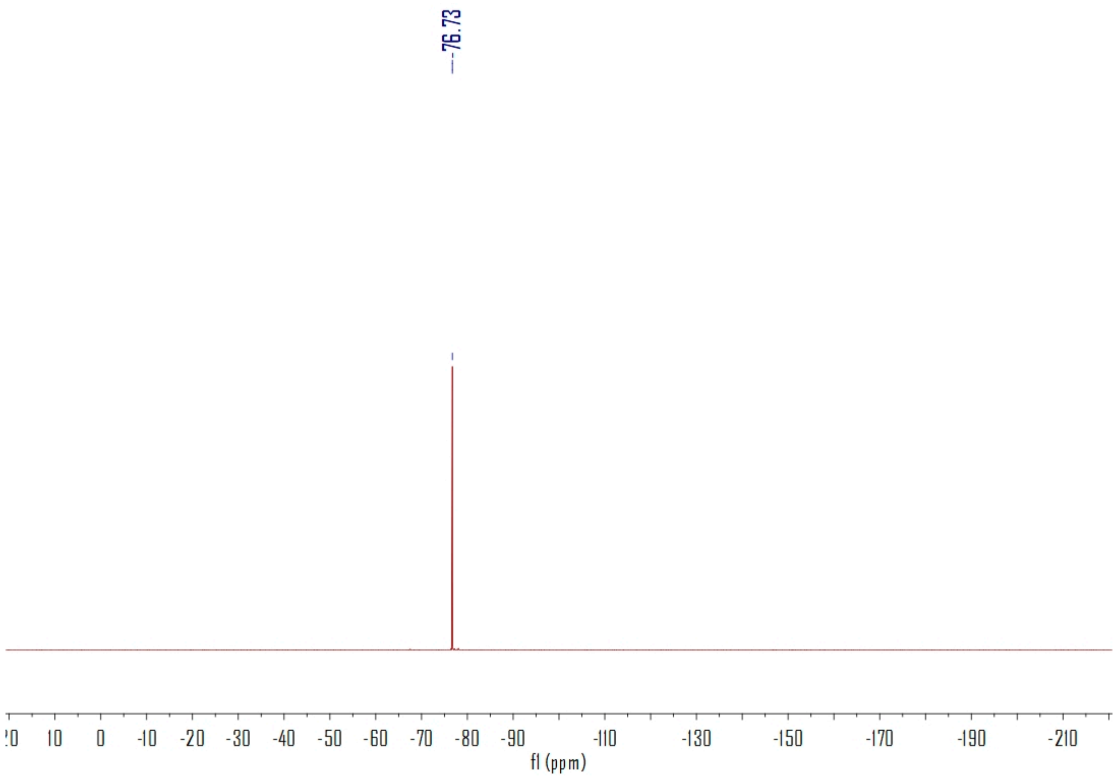

Figure S56. ^{1}H NMR spectrum of the compound (**3hc**)

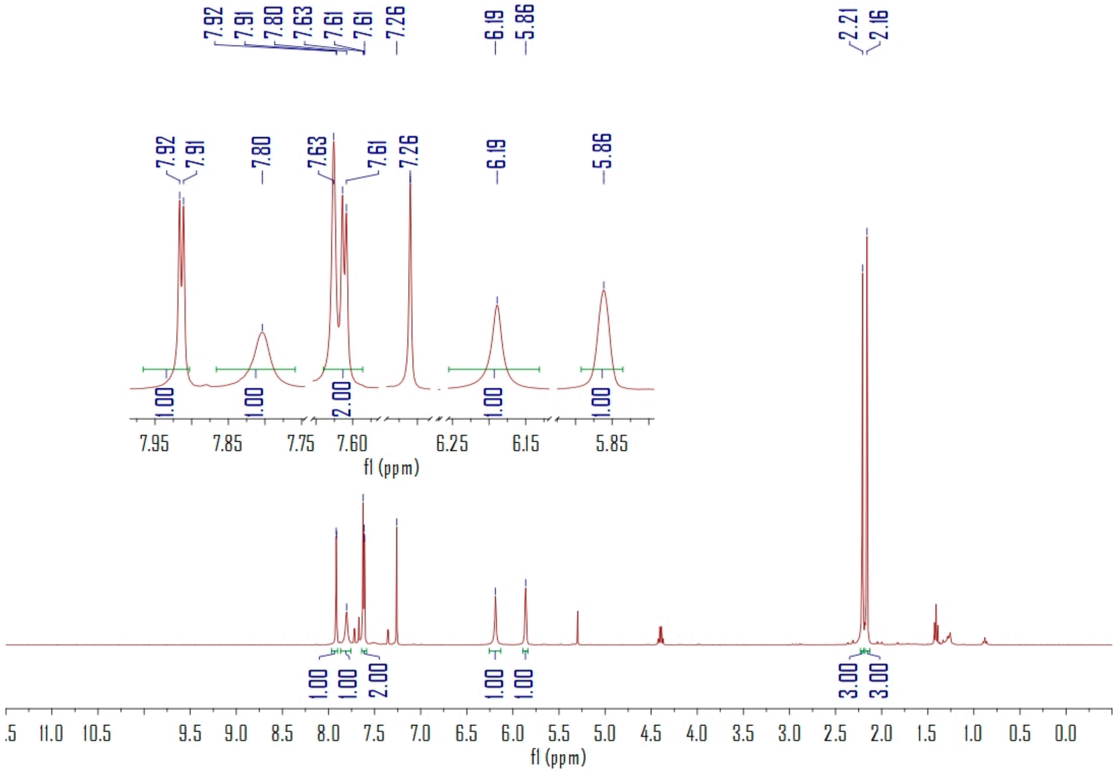

Figure S57. ^{13}C NMR spectrum of the compound (**3hc**)

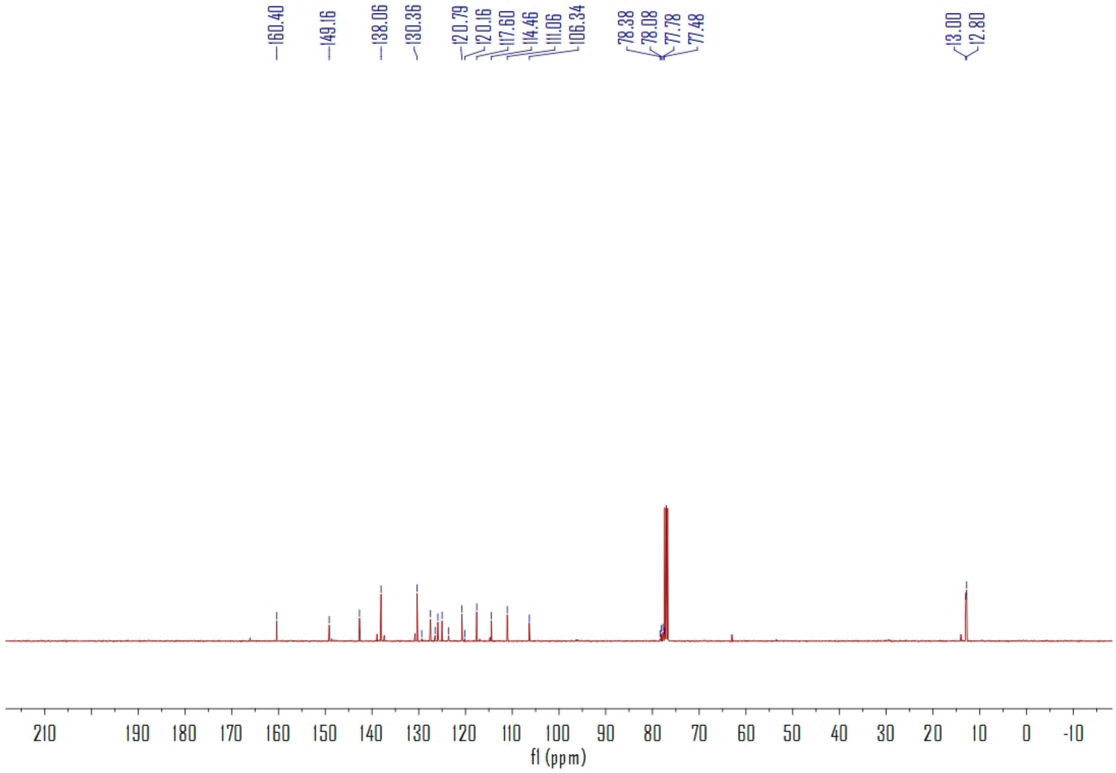

Figure S58. ^{19}F NMR spectrum of the compound (**3hc**)

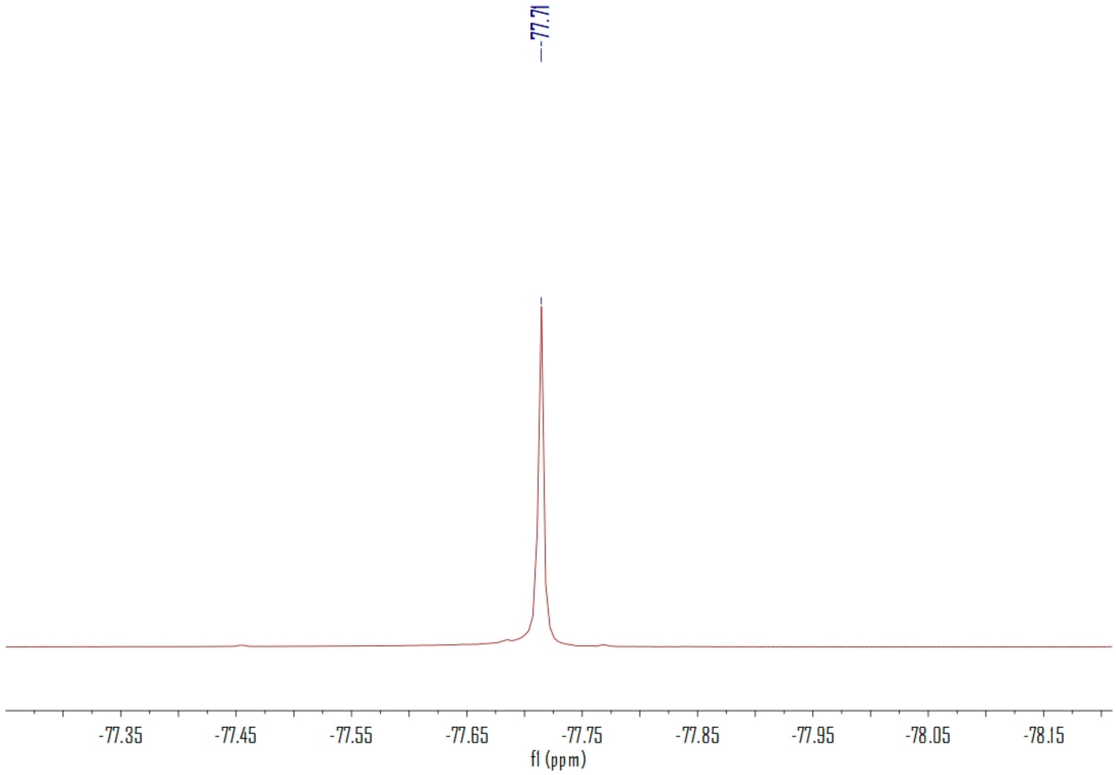

Figure S59. ^{1}H NMR spectrum of the compound (**3ic**)

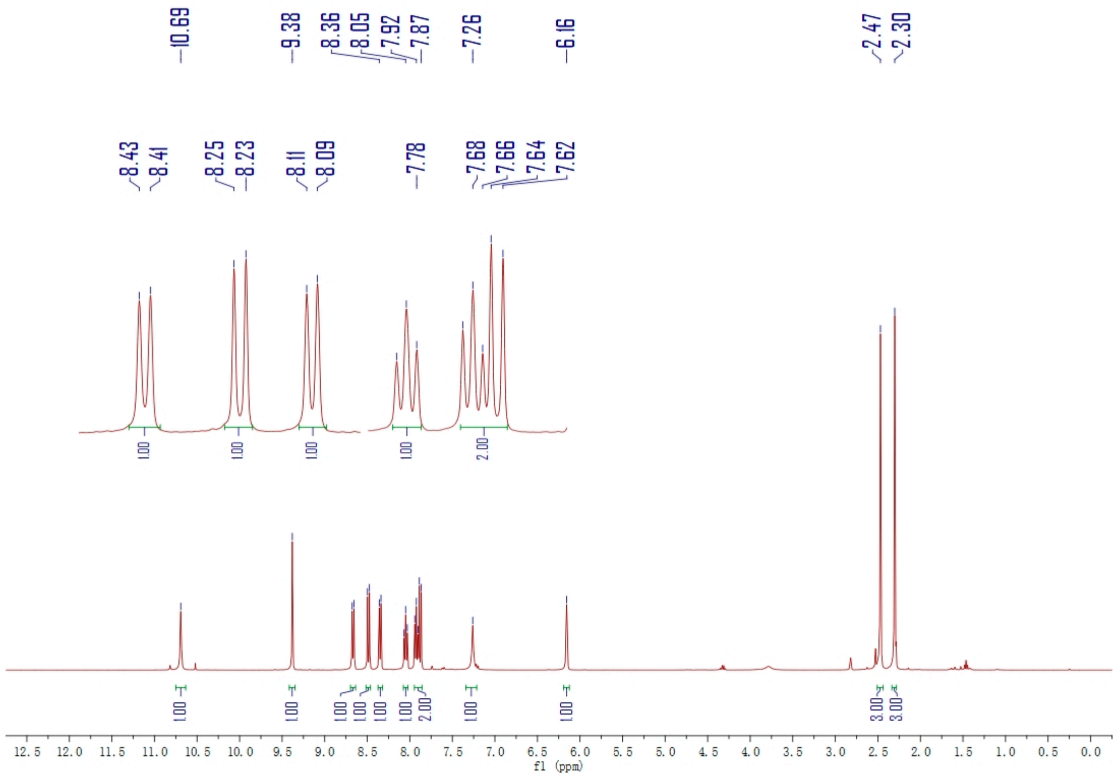

Figure S60. ^{13}C NMR spectrum of the compound (**3ic**)

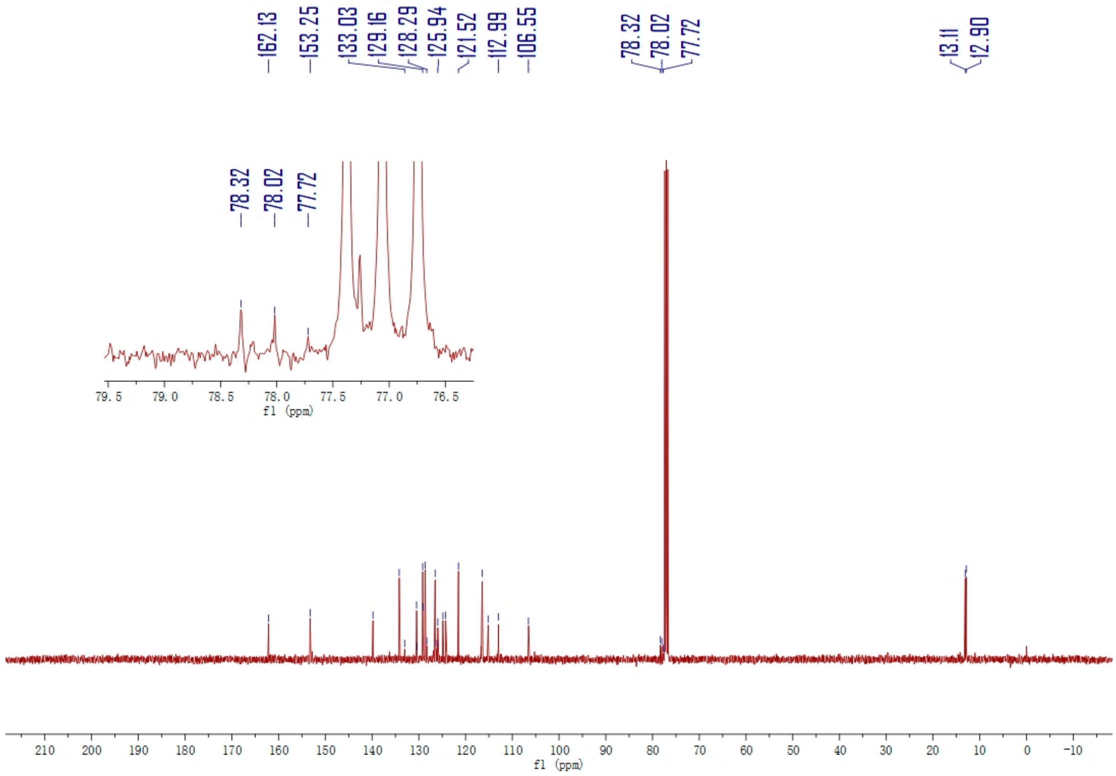

Figure S61. ^{19}F NMR spectrum of the compound (**3ic**)

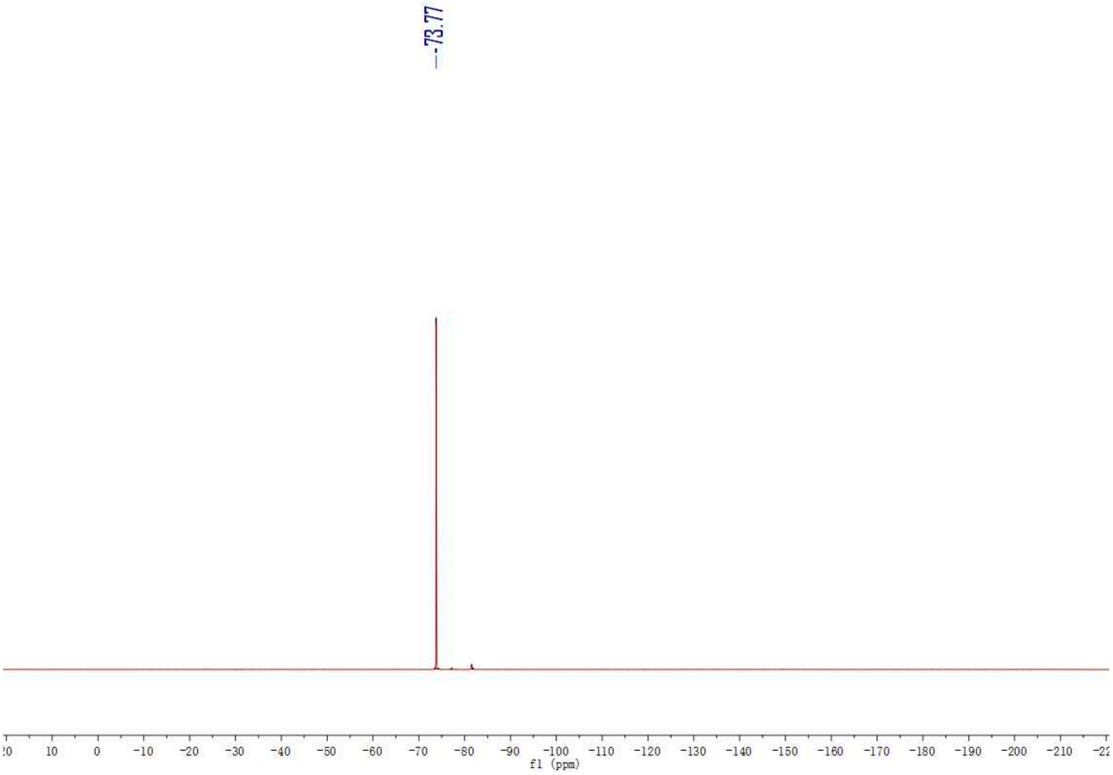

Figure S62. ^{1}H NMR spectrum of the compound (**3ad**)

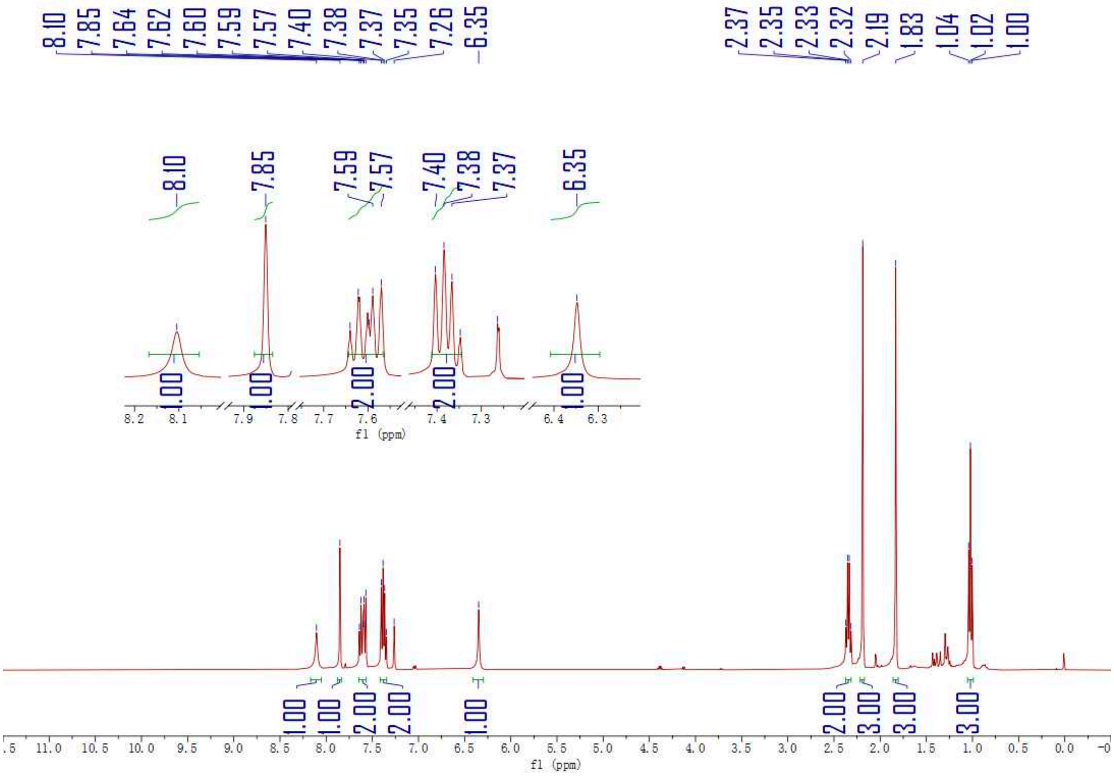

Figure S63. ^{13}C NMR spectrum of the compound (**3ad**)

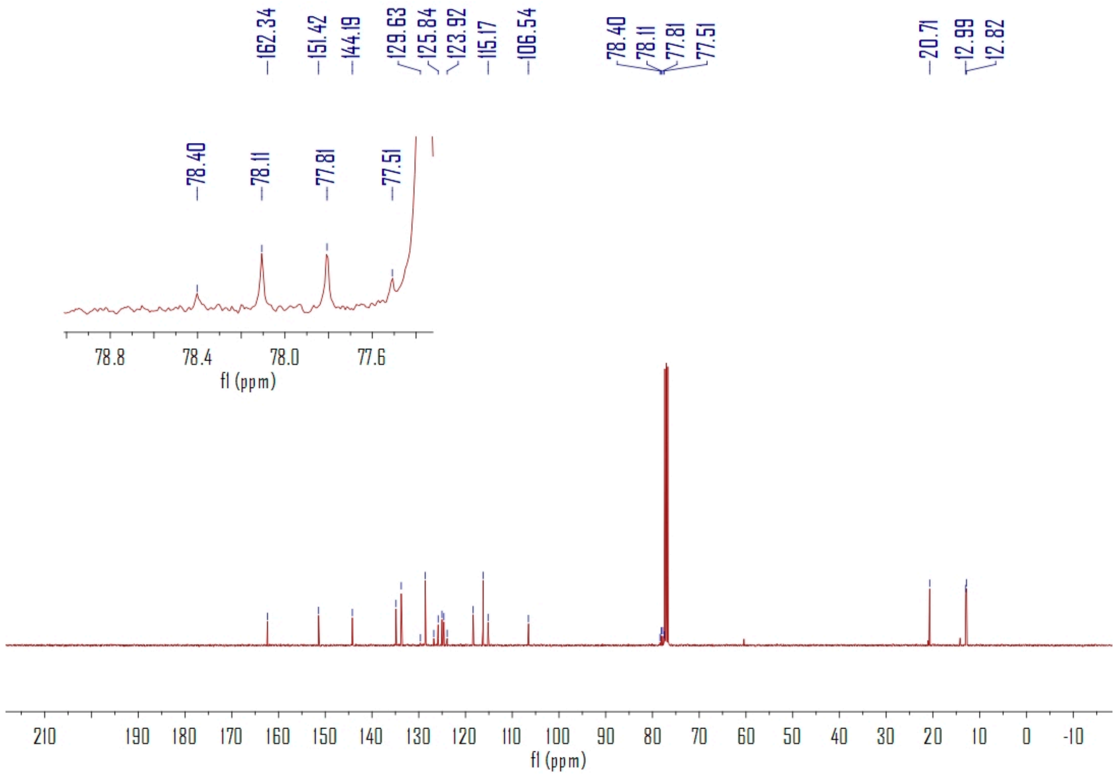

Figure S64. ^{19}F NMR spectrum of the compound (**3ad**)

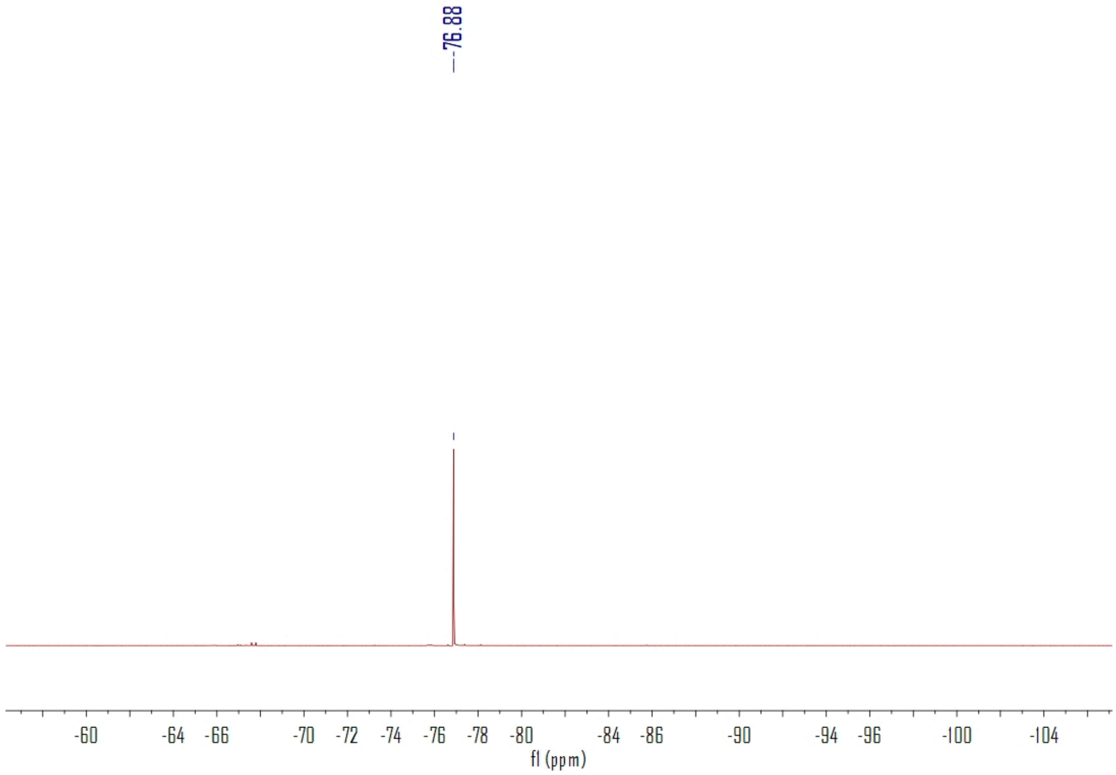

Figure S65. ^{1}H NMR spectrum of the compound (**3bd**)

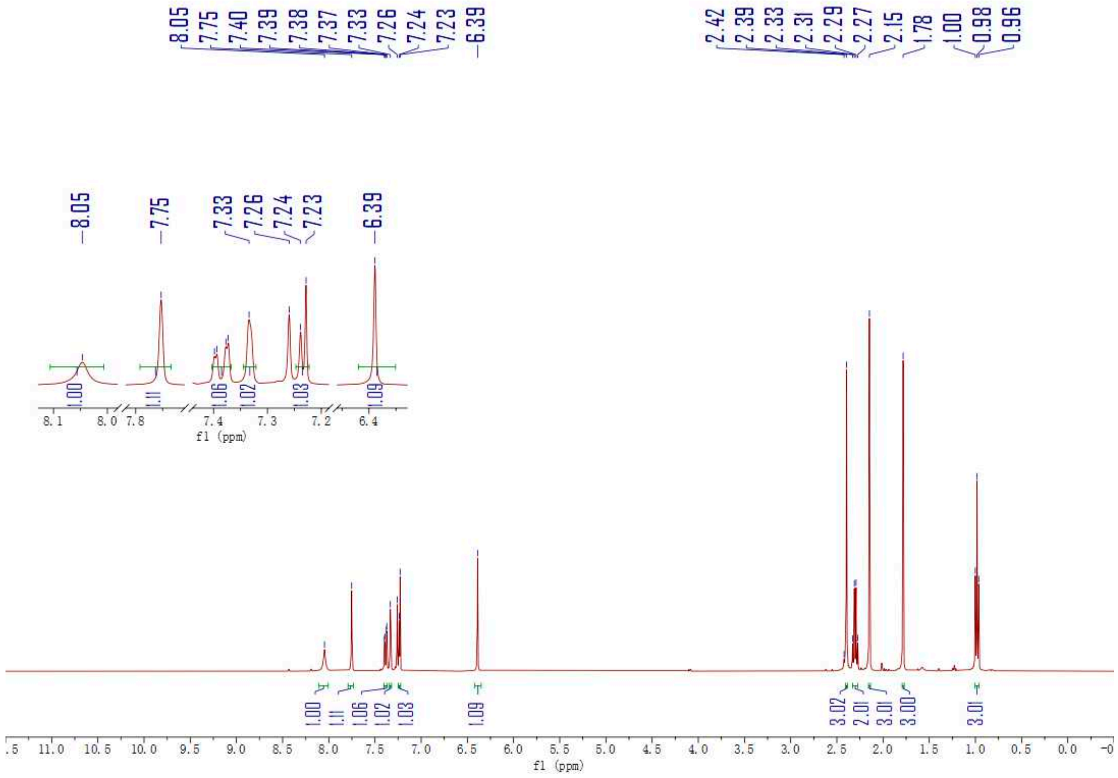

Figure S66. ^{13}C NMR spectrum of the compound (**3bd**)

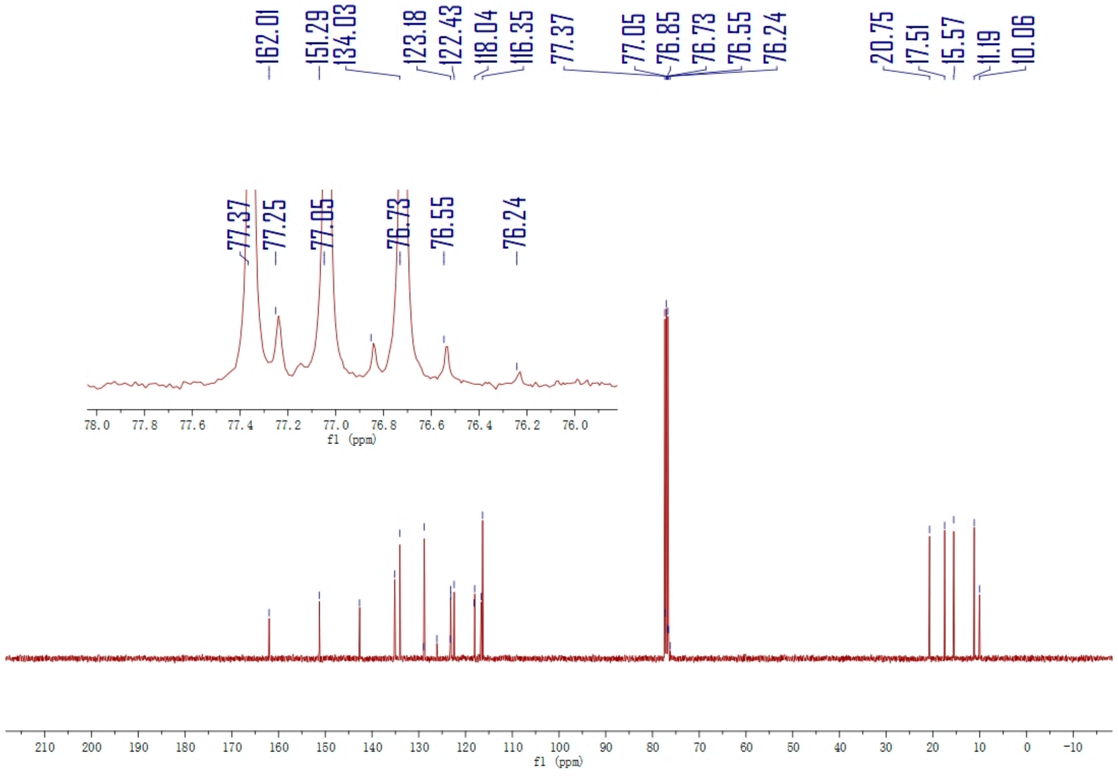

Figure S67. ^{19}F NMR spectrum of the compound (**3bd**)

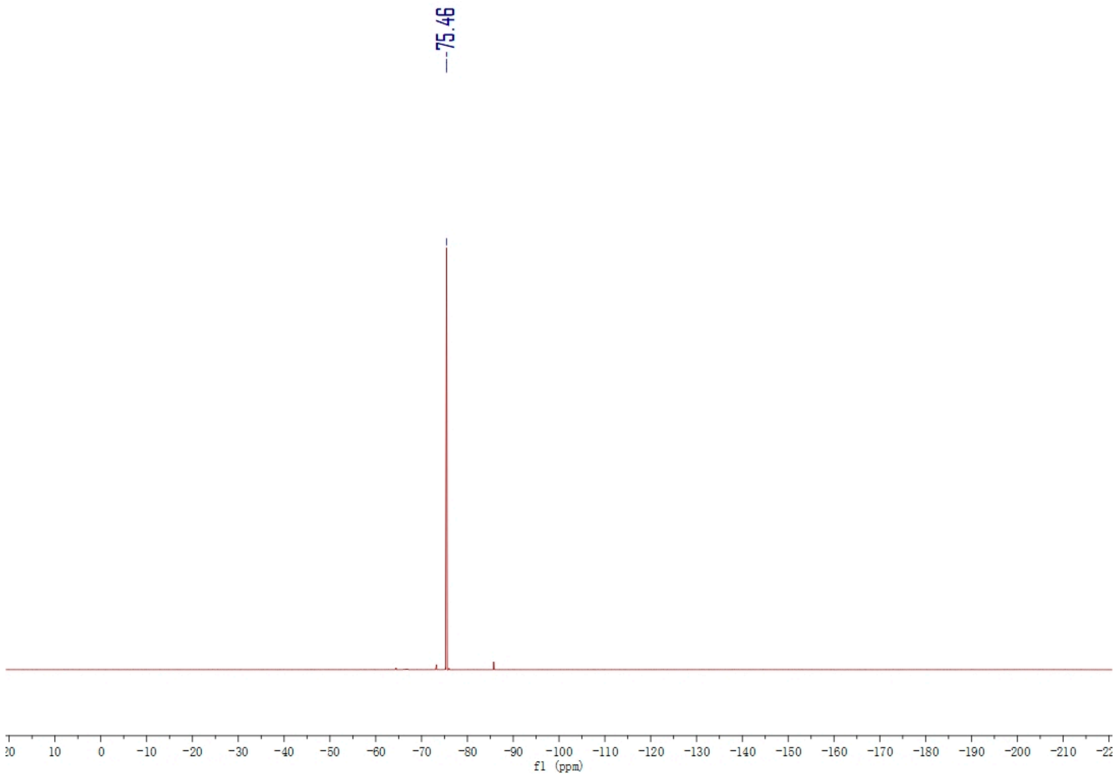

Figure S68. ^{1}H NMR spectrum of the compound (**3cd**)

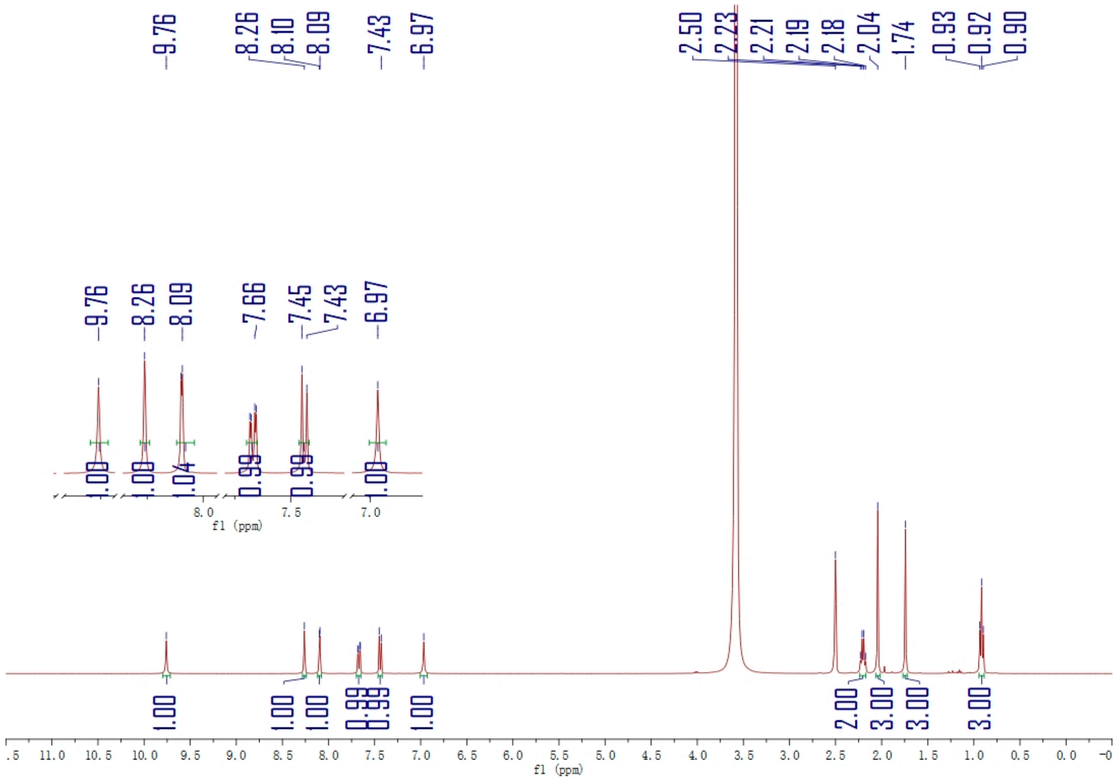

Figure S69. ^{13}C NMR spectrum of the compound (**3cd**)

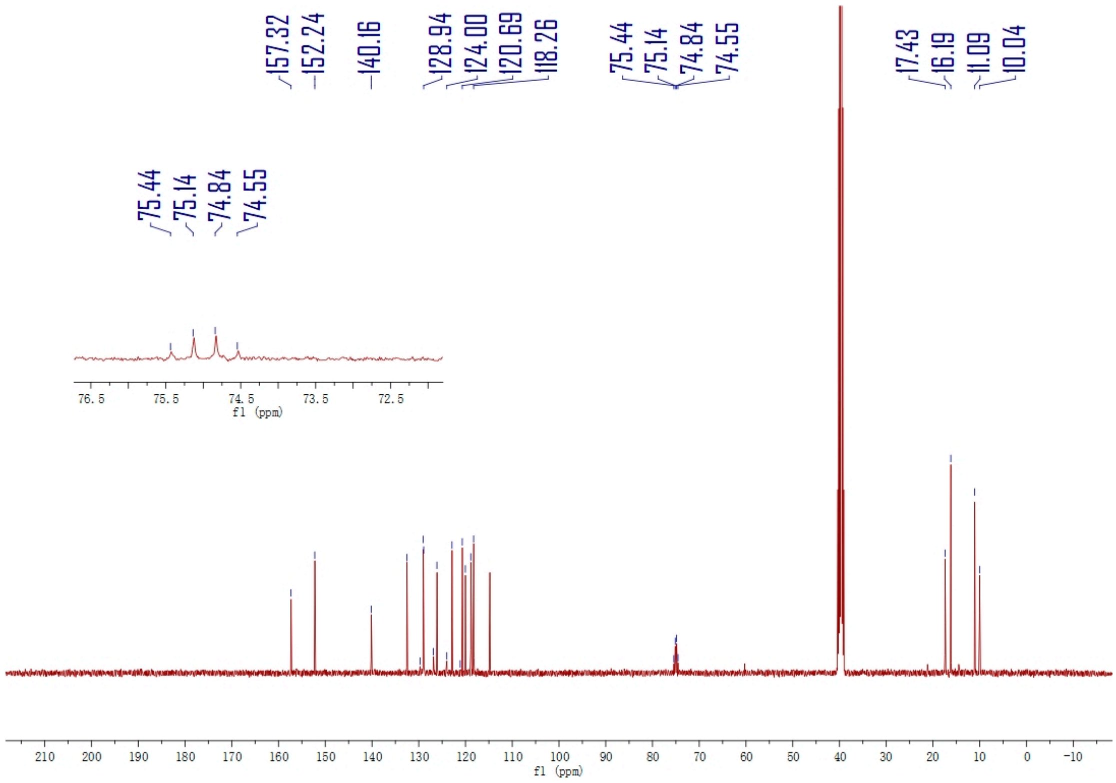

Figure S70. ^{19}F NMR spectrum of the compound (**3cd**)

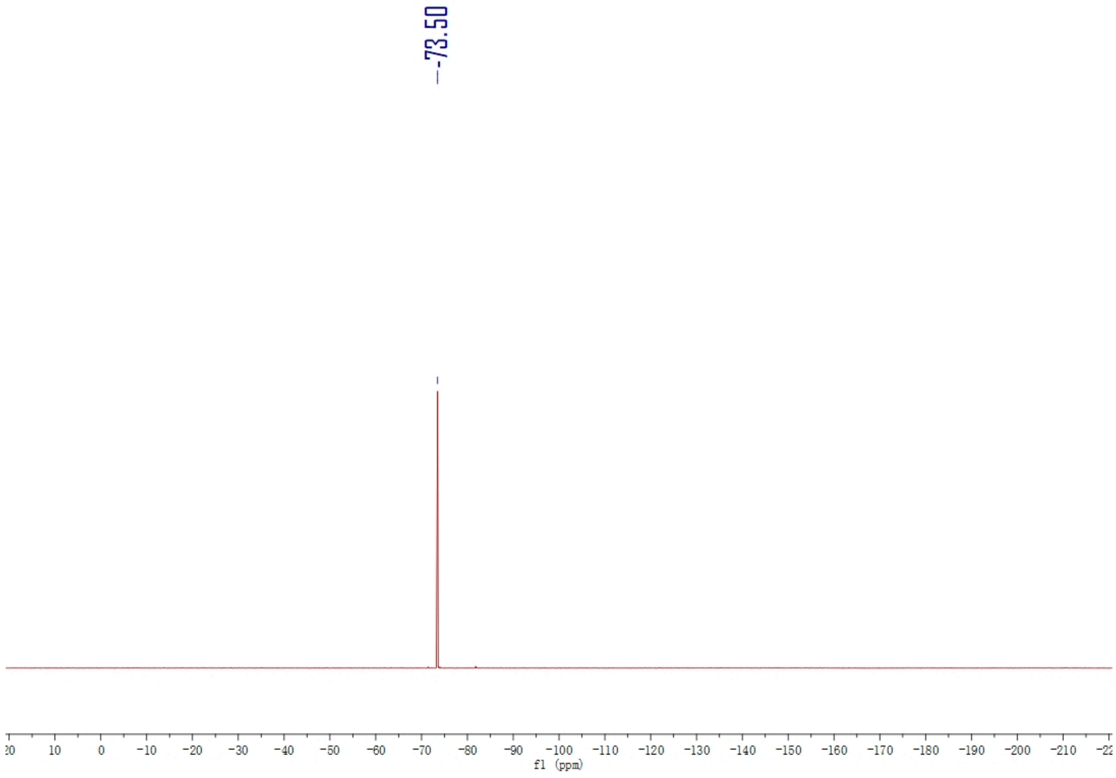

Figure S71. ^{1}H NMR spectrum of the compound (**3dd**)

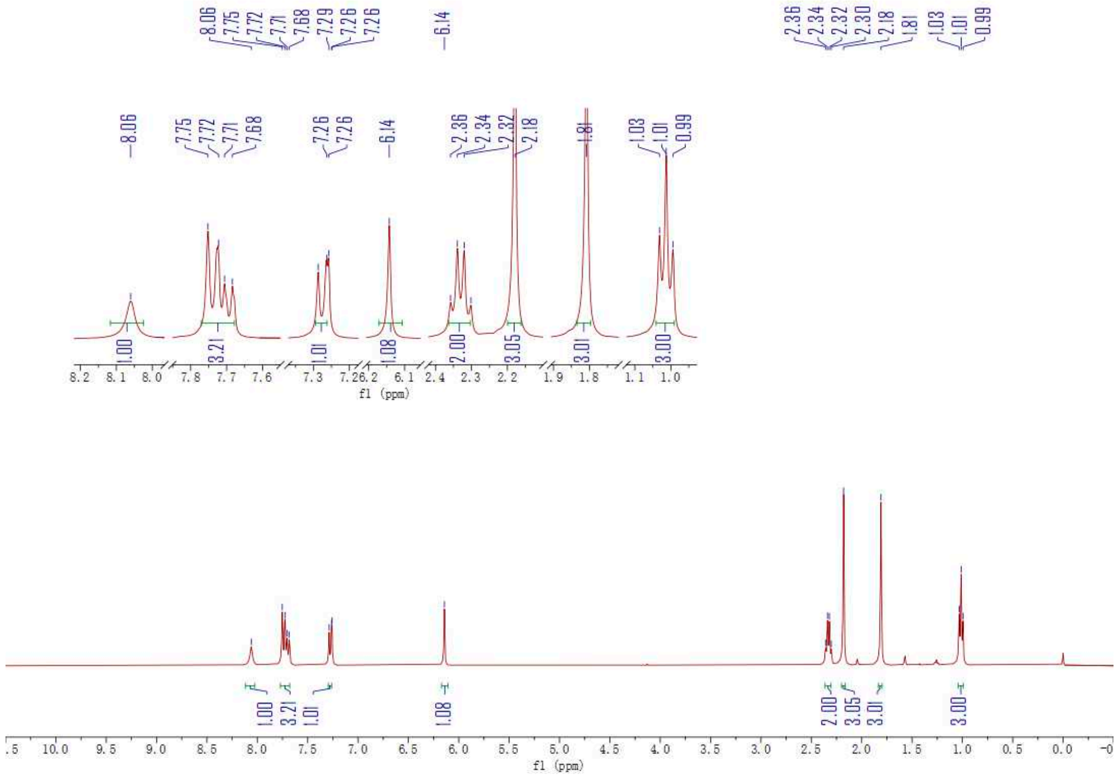

Figure S72. ^{13}C NMR spectrum of the compound (**3dd**)

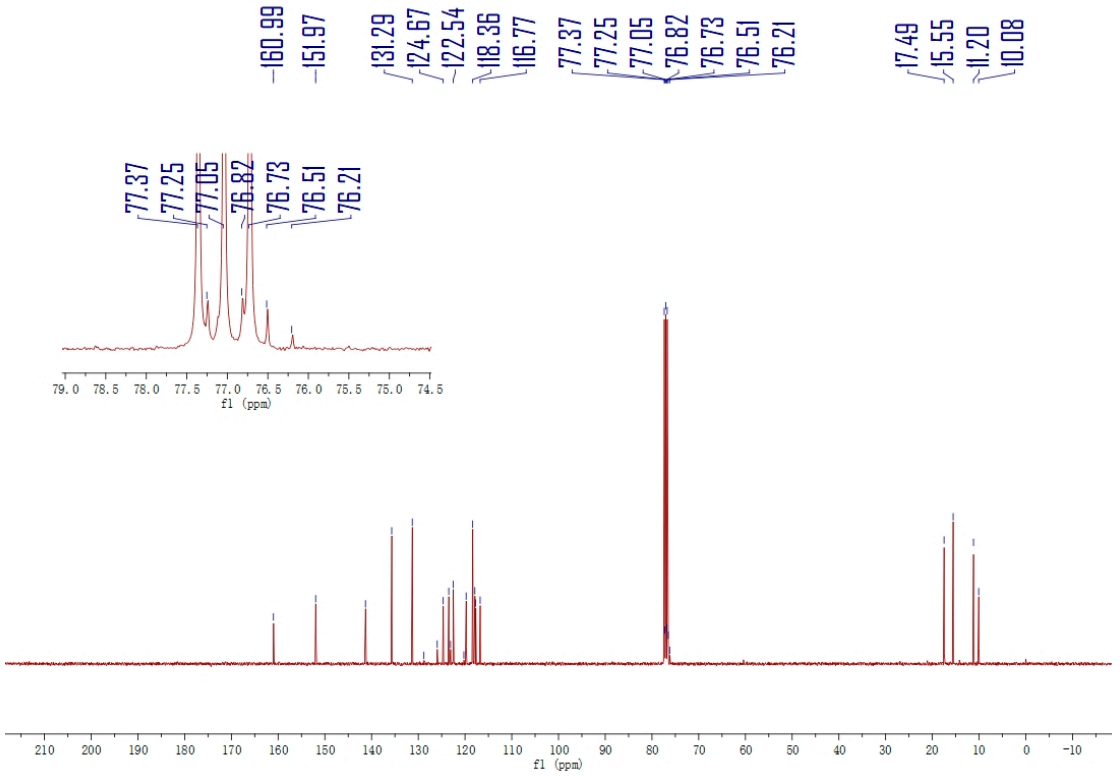

Figure S73. ^{19}F NMR spectrum of the compound (**3dd**)

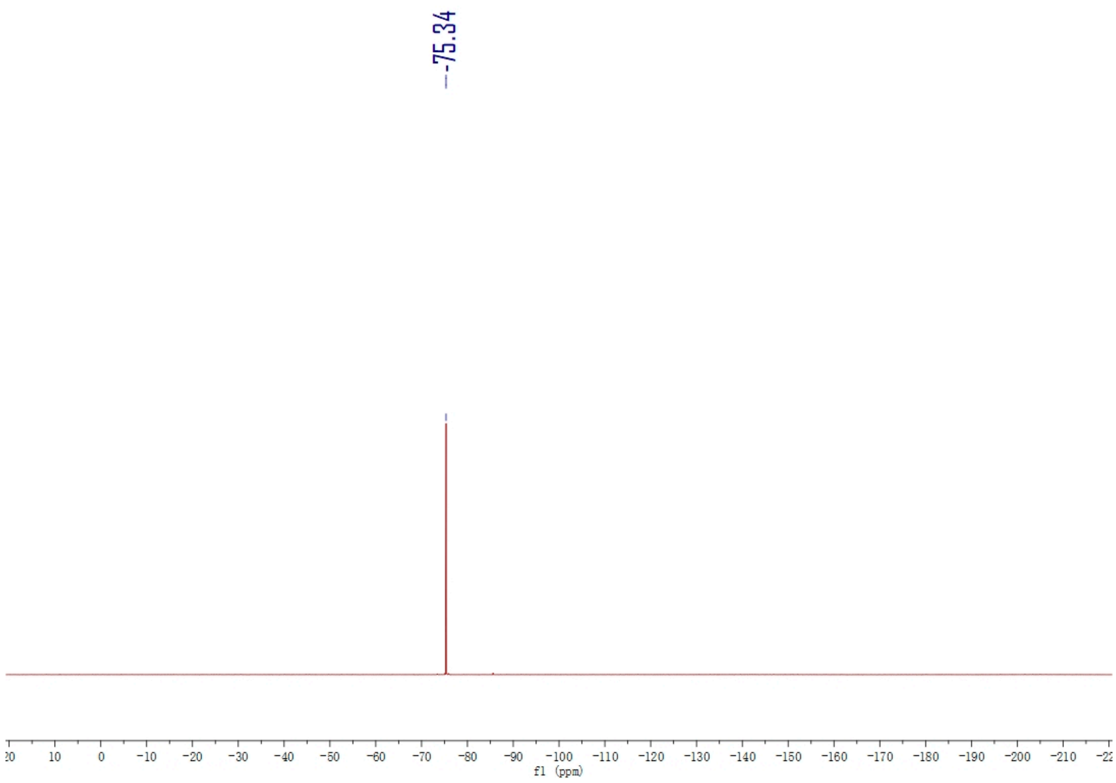

Figure S74. ^{1}H NMR spectrum of the compound (**3ed**)

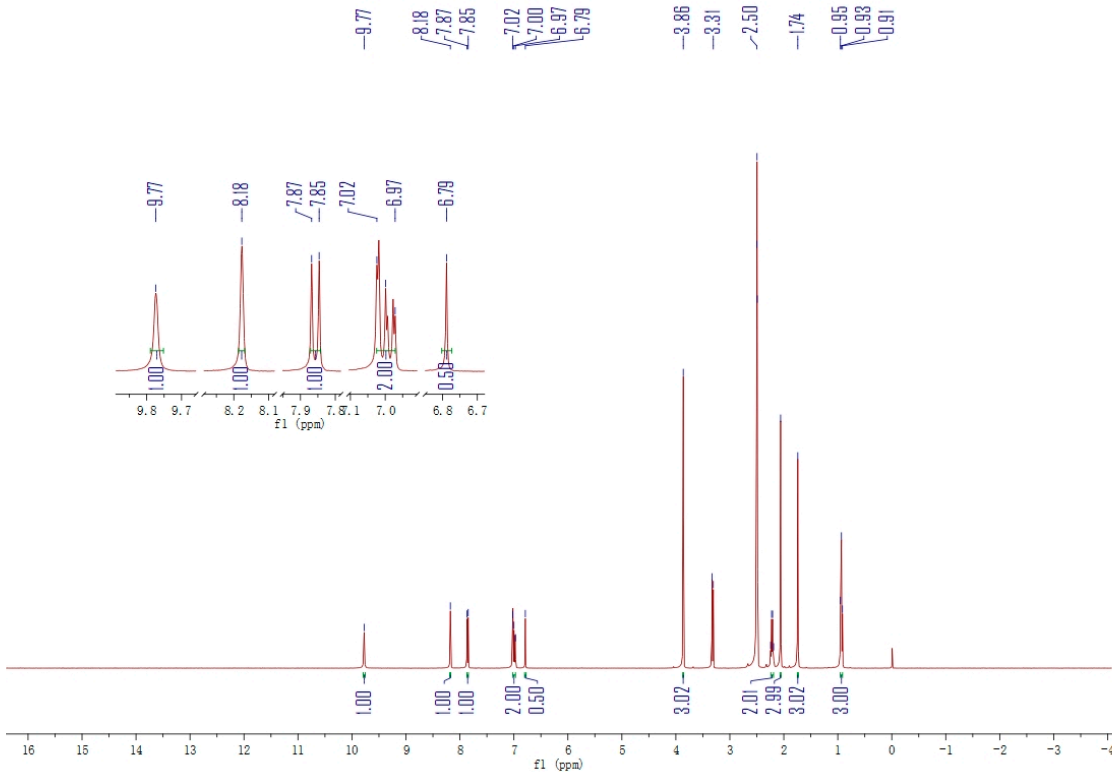

Figure S75. ^{13}C NMR spectrum of the compound (**3ed**)

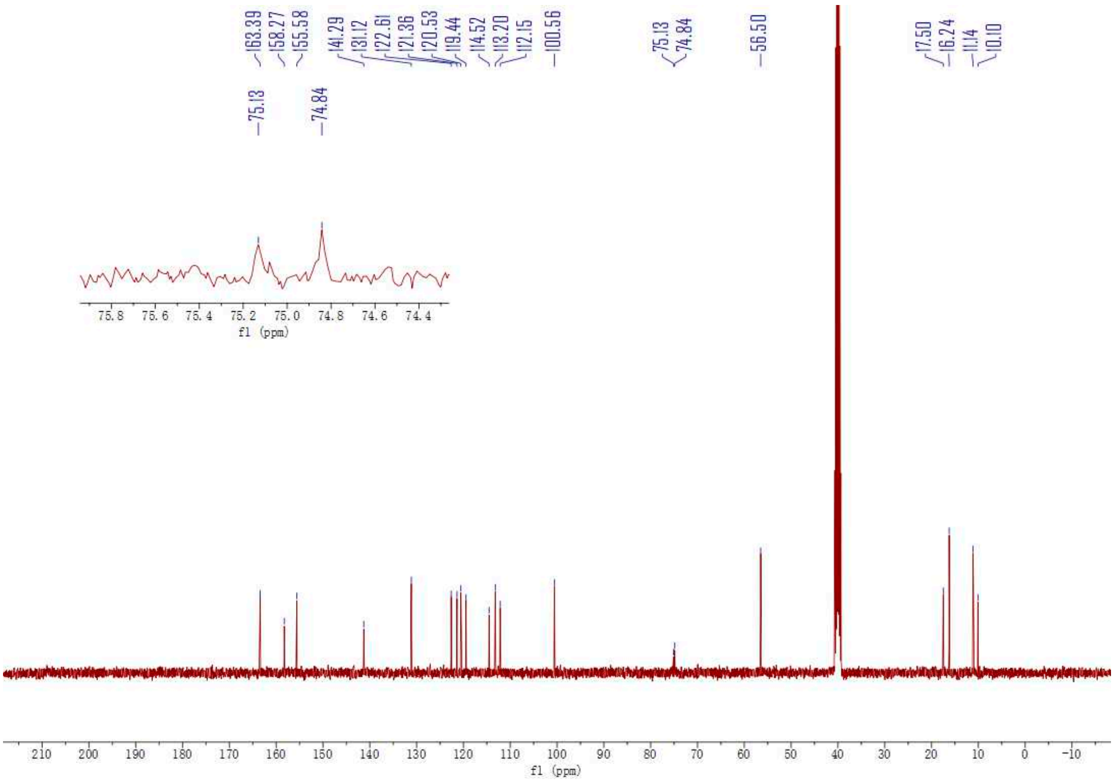

Figure S76. ^{19}F NMR spectrum of the compound (**3ed**)

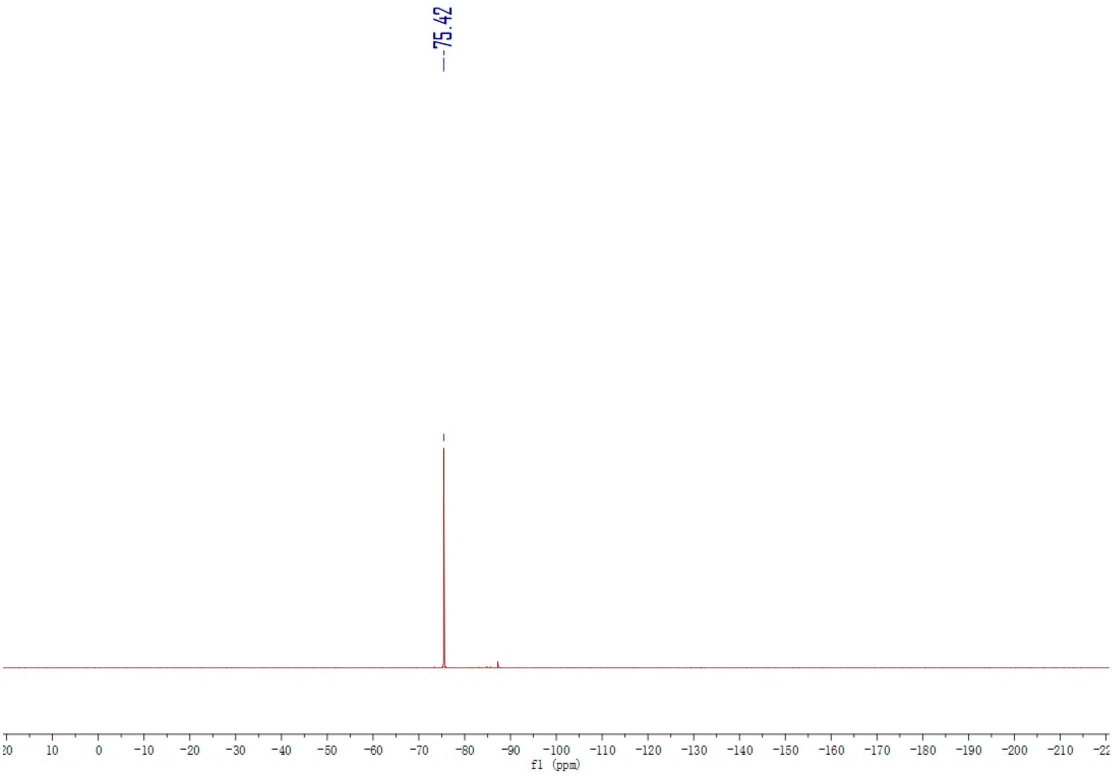

Figure S77. ^{1}H NMR spectrum of the compound (**3gd**)

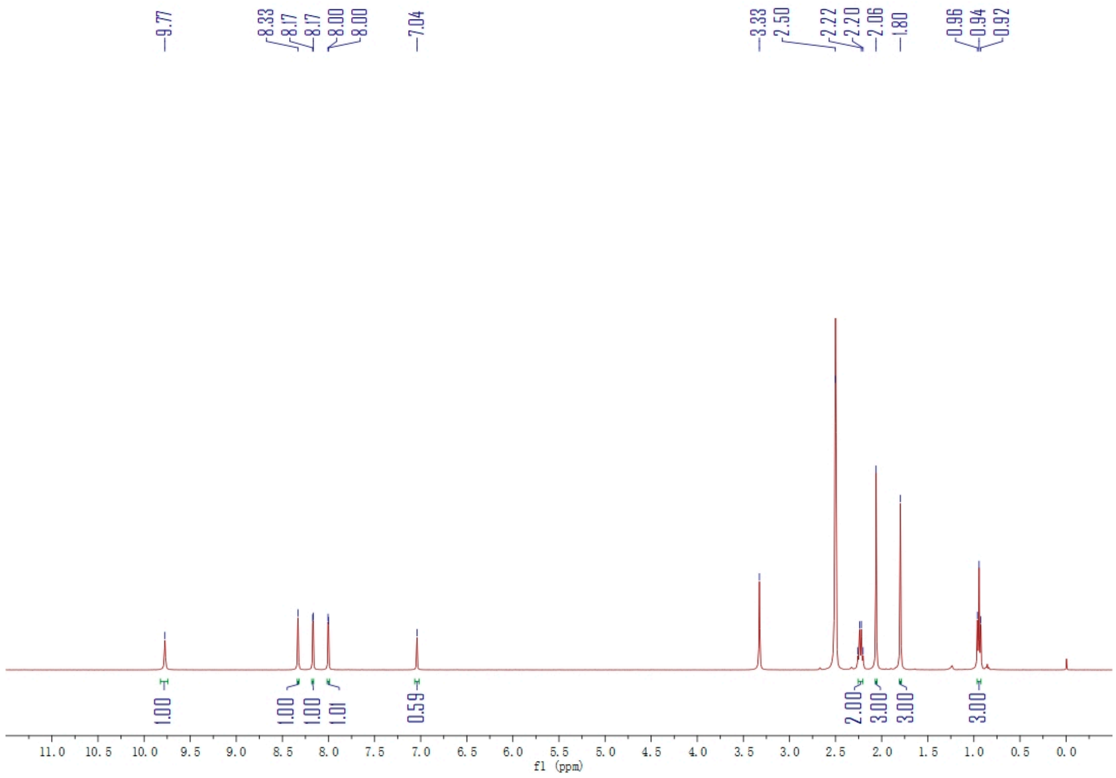

Figure S78. ^{13}C NMR spectrum of the compound (**3gd**)

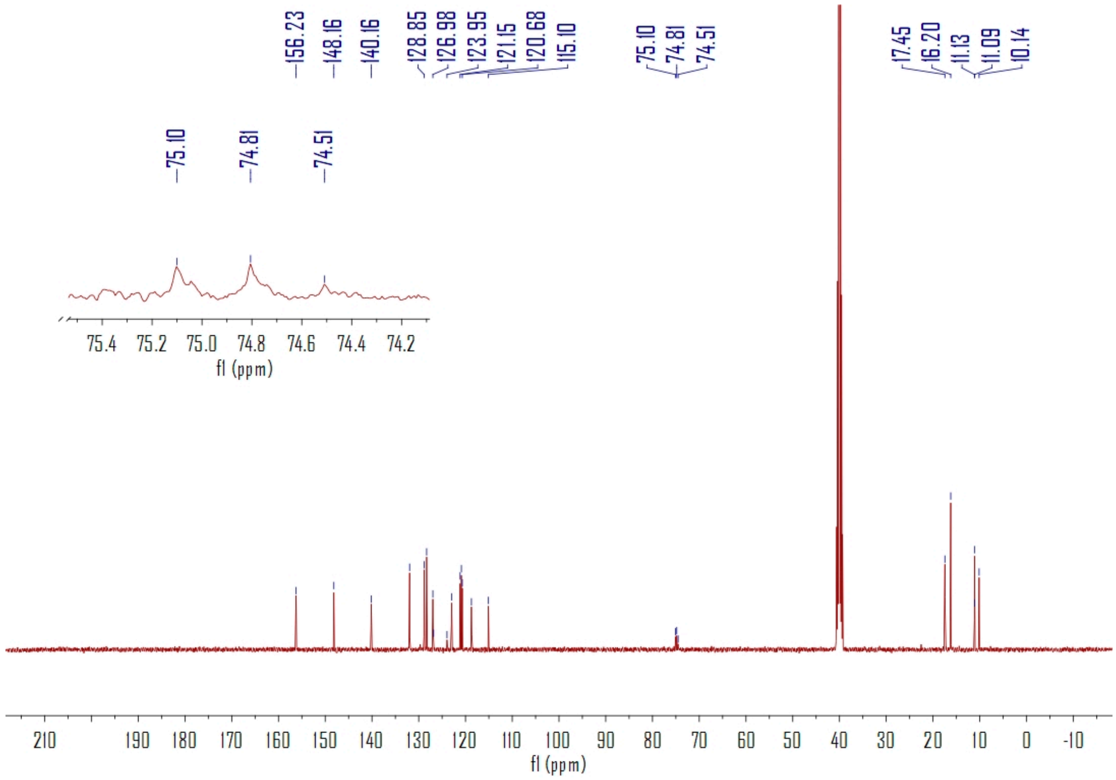

Figure S79. ^{19}F NMR spectrum of the compound (**3gd**)

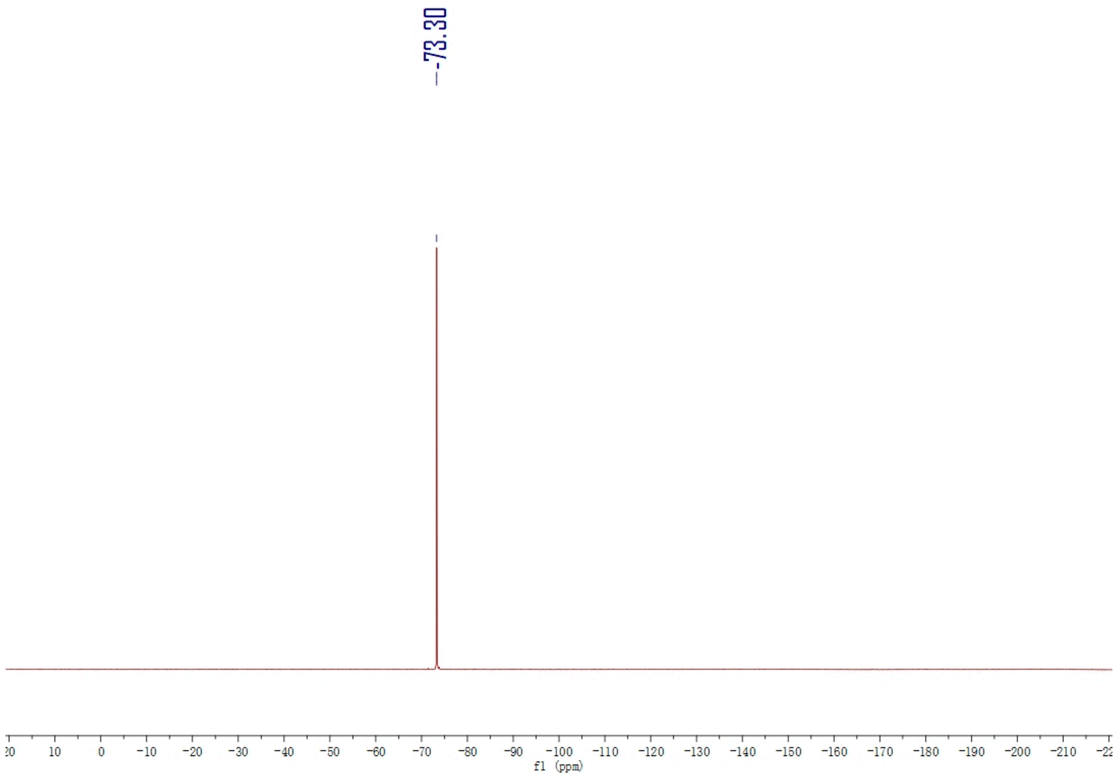

Figure S80. ^{1}H NMR spectrum of the compound (**3hd**)

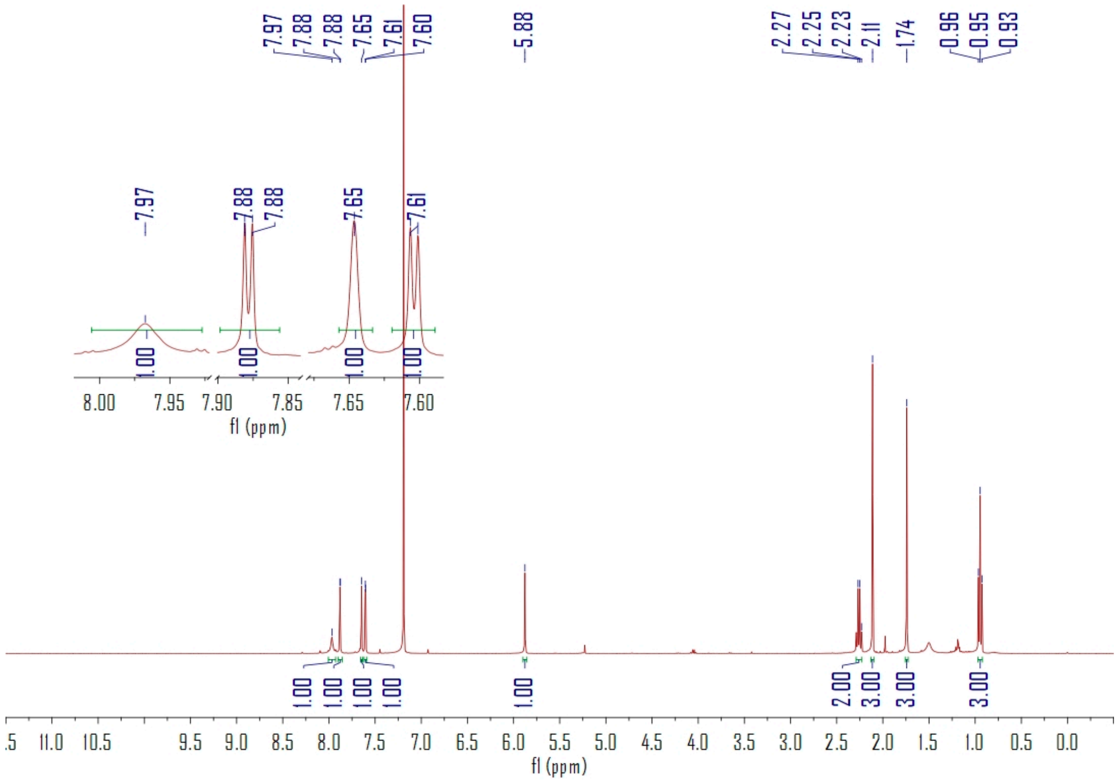

Figure S81. ^{13}C NMR spectrum of the compound (**3hd**)

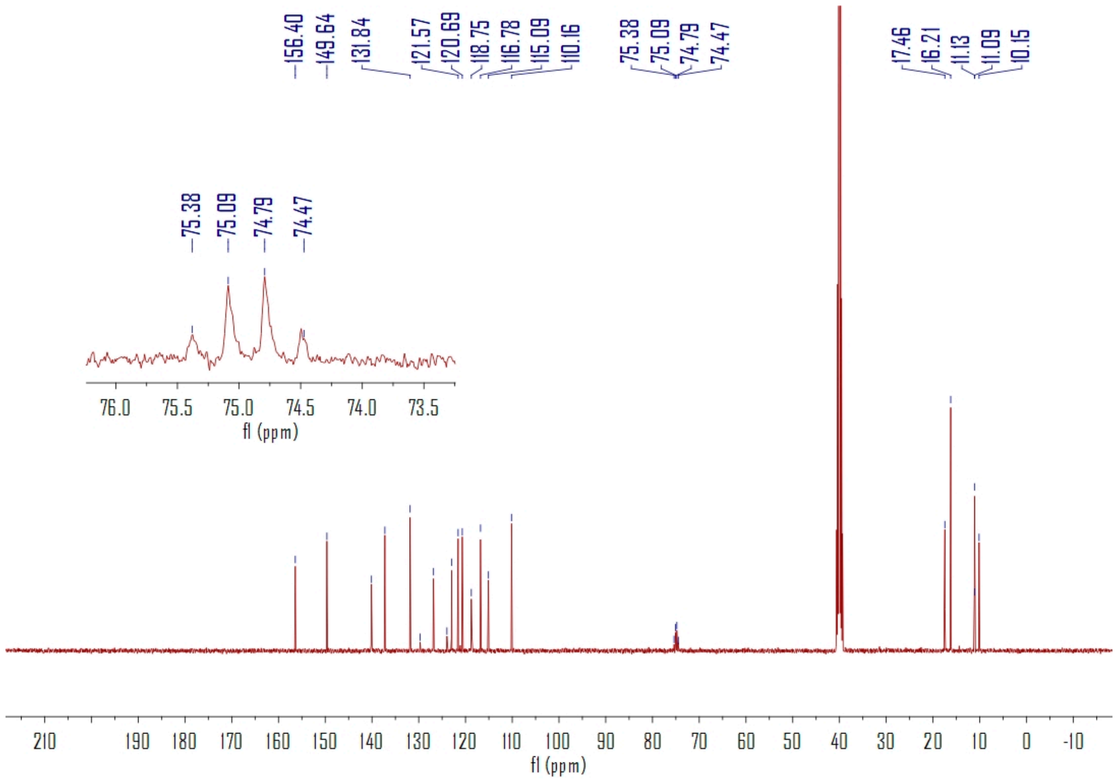

Figure S82. ^{19}F NMR spectrum of the compound (**3hd**)

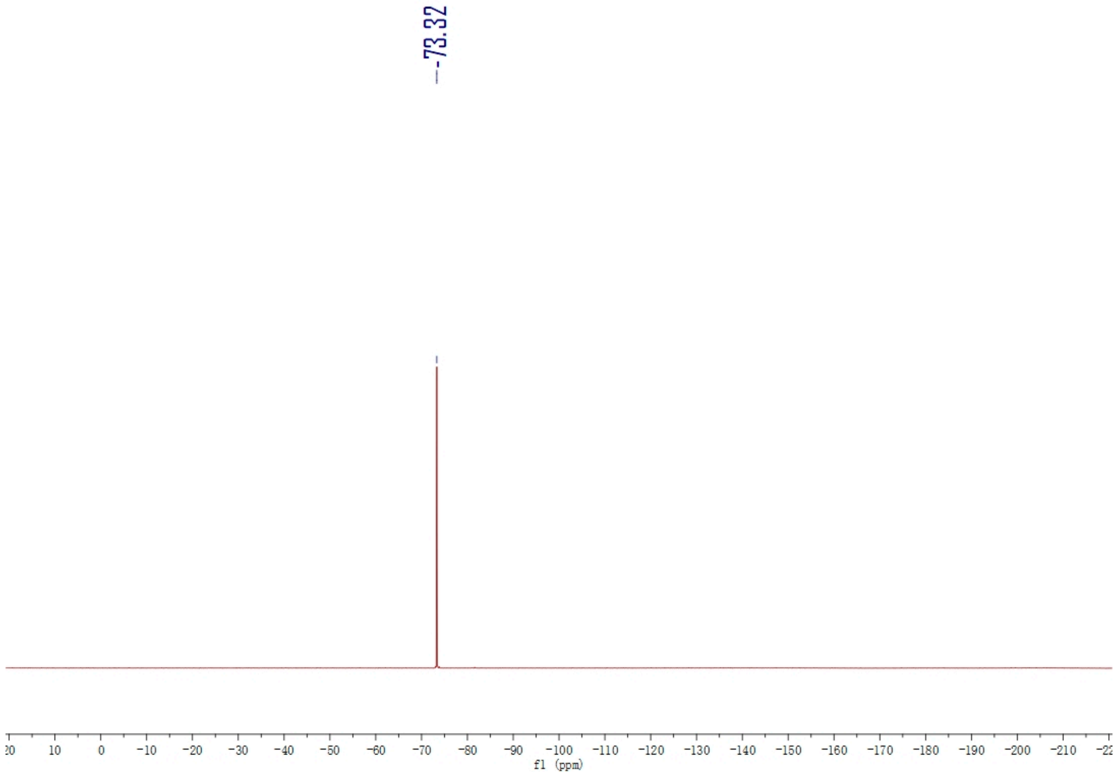

Figure S83. ^{1}H NMR spectrum of the compound (**3ae**)

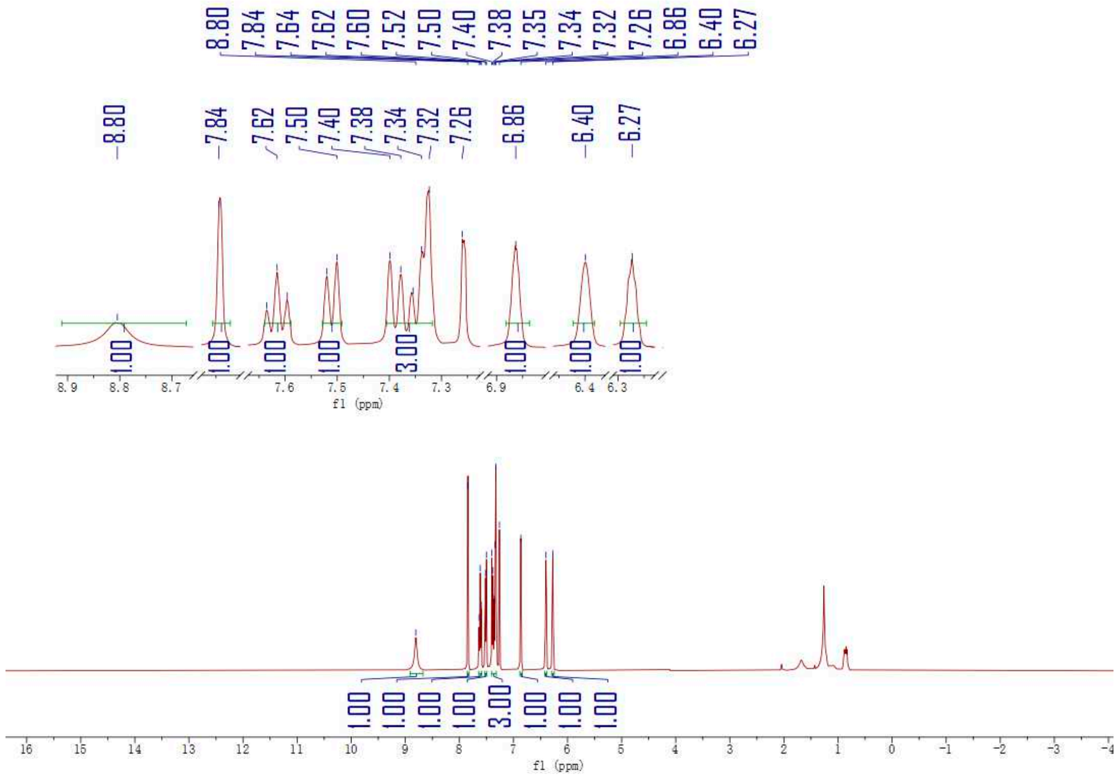

Figure S84. ^{13}C NMR spectrum of the compound (**3ae**)

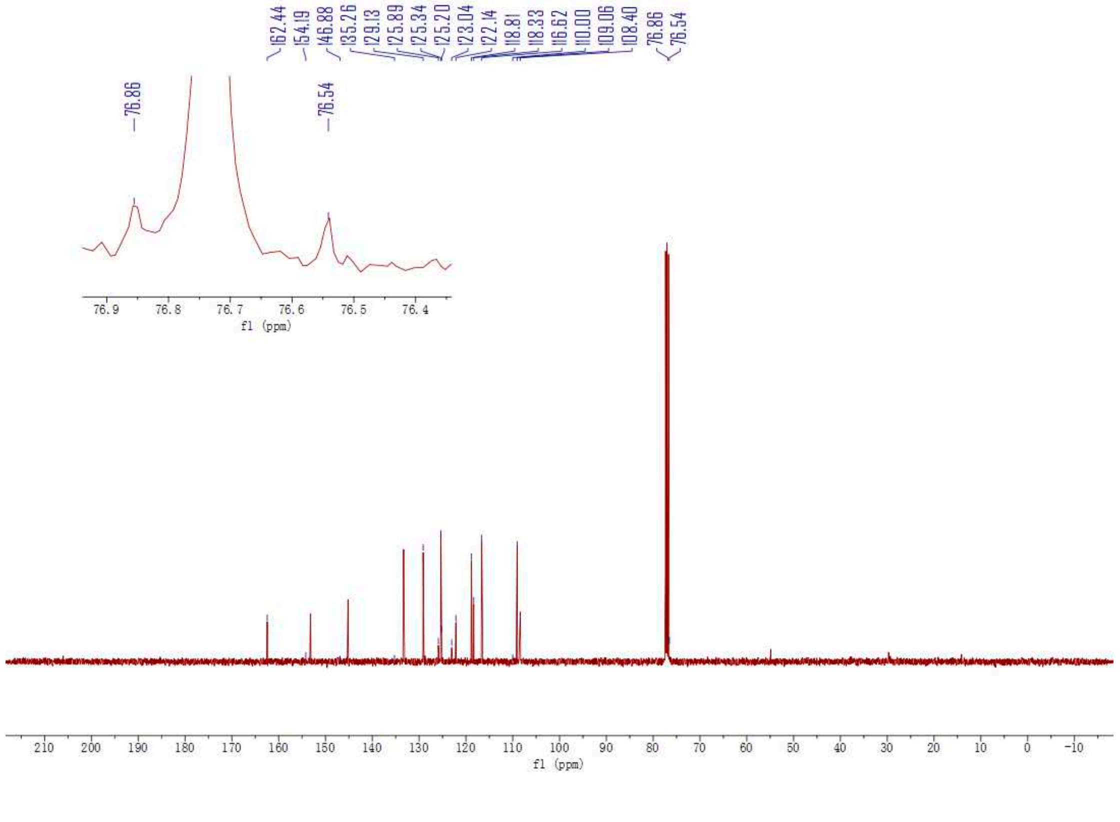

Figure S85. ^{19}F NMR spectrum of the compound (**3ae**)

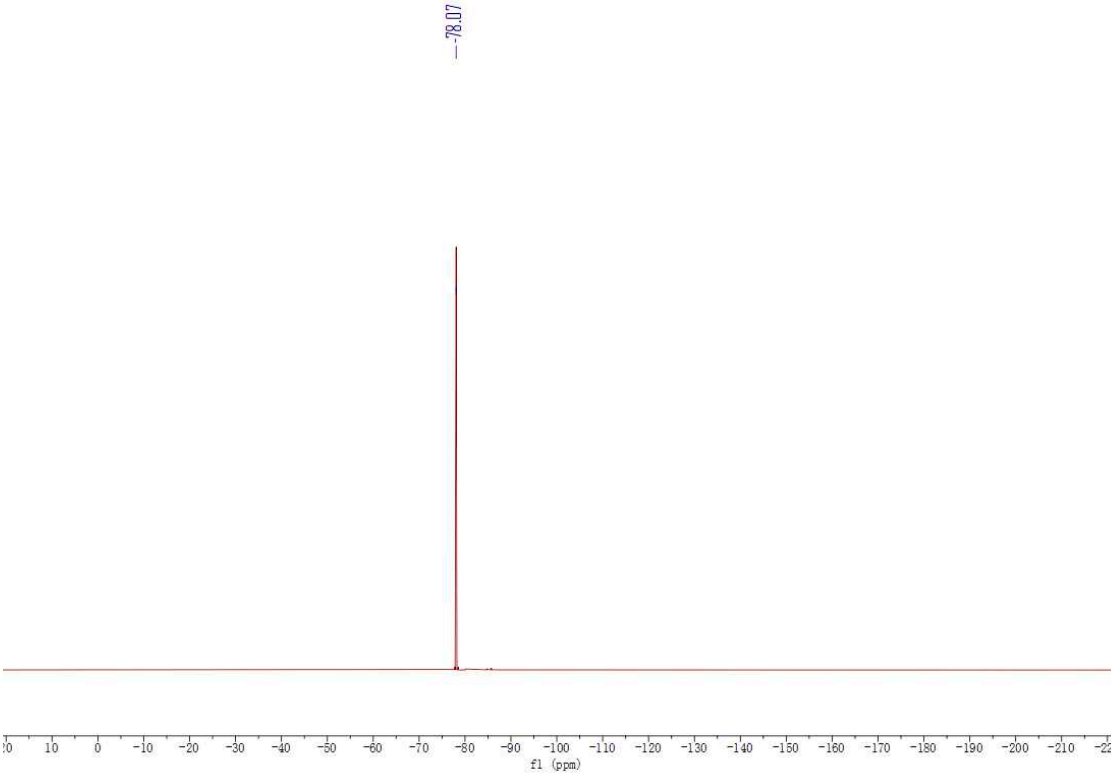

Figure S86. ^{1}H NMR spectrum of the compound (**3af**)

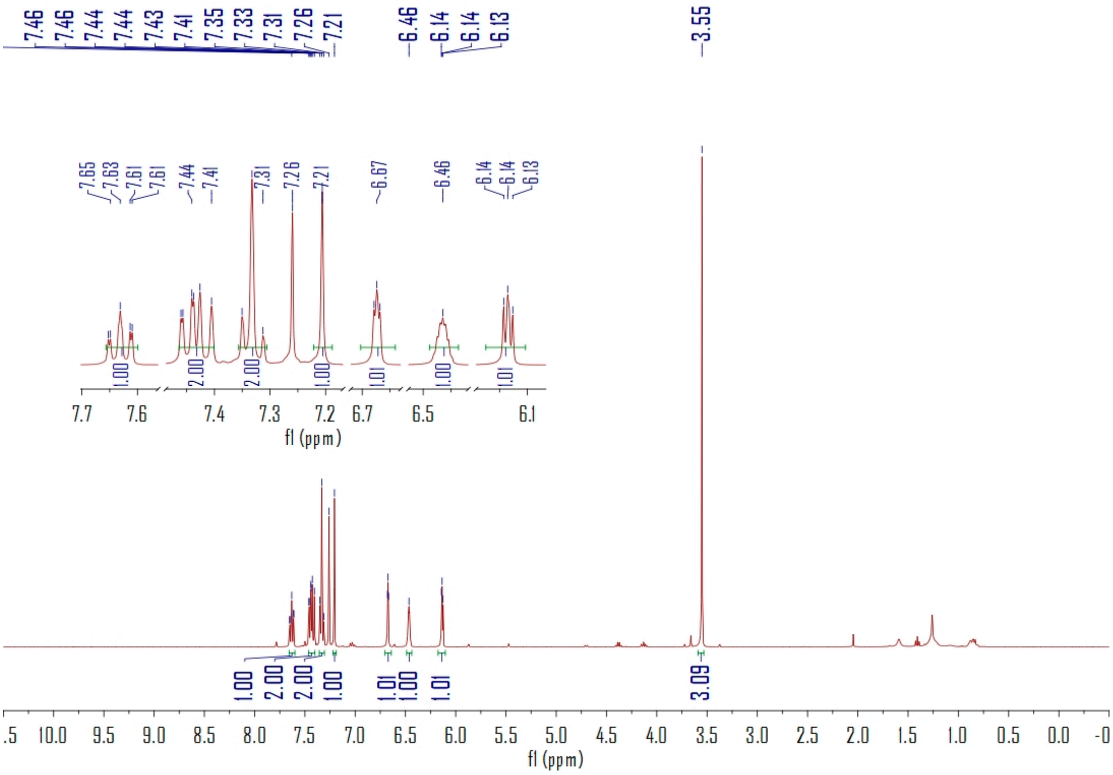

Figure S87. ^{13}C NMR spectrum of the compound (**3af**)

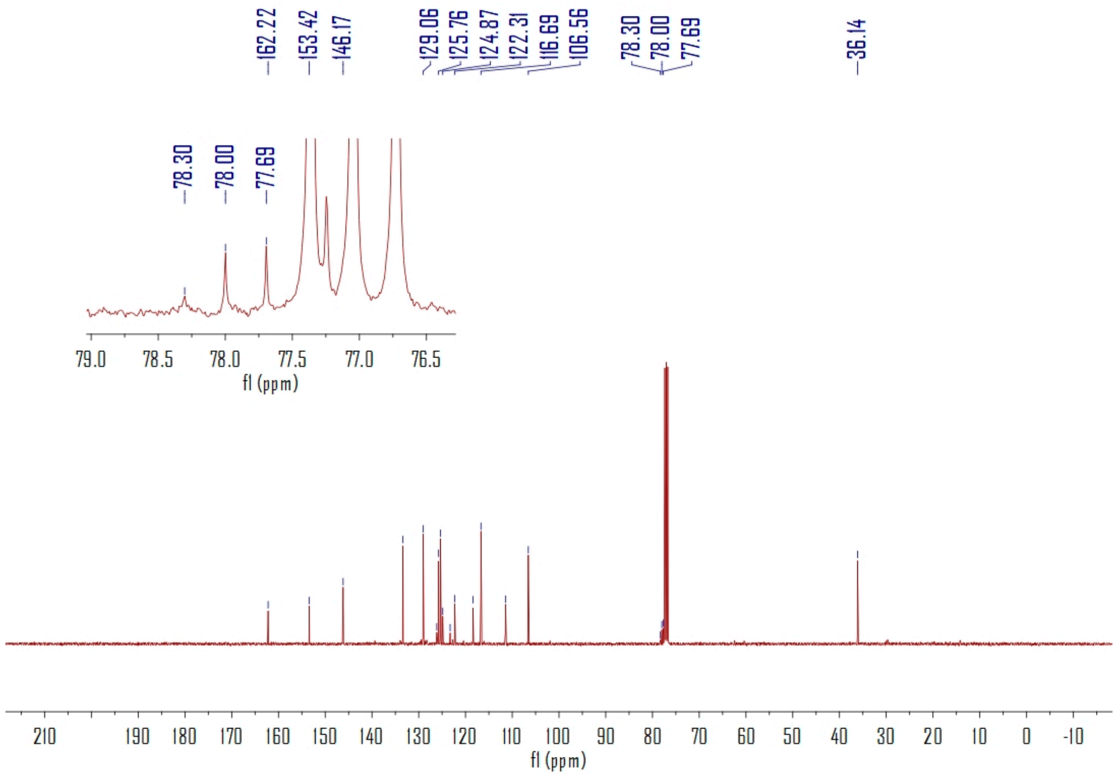

Figure S88. ^{19}F NMR spectrum of the compound (**3af**)

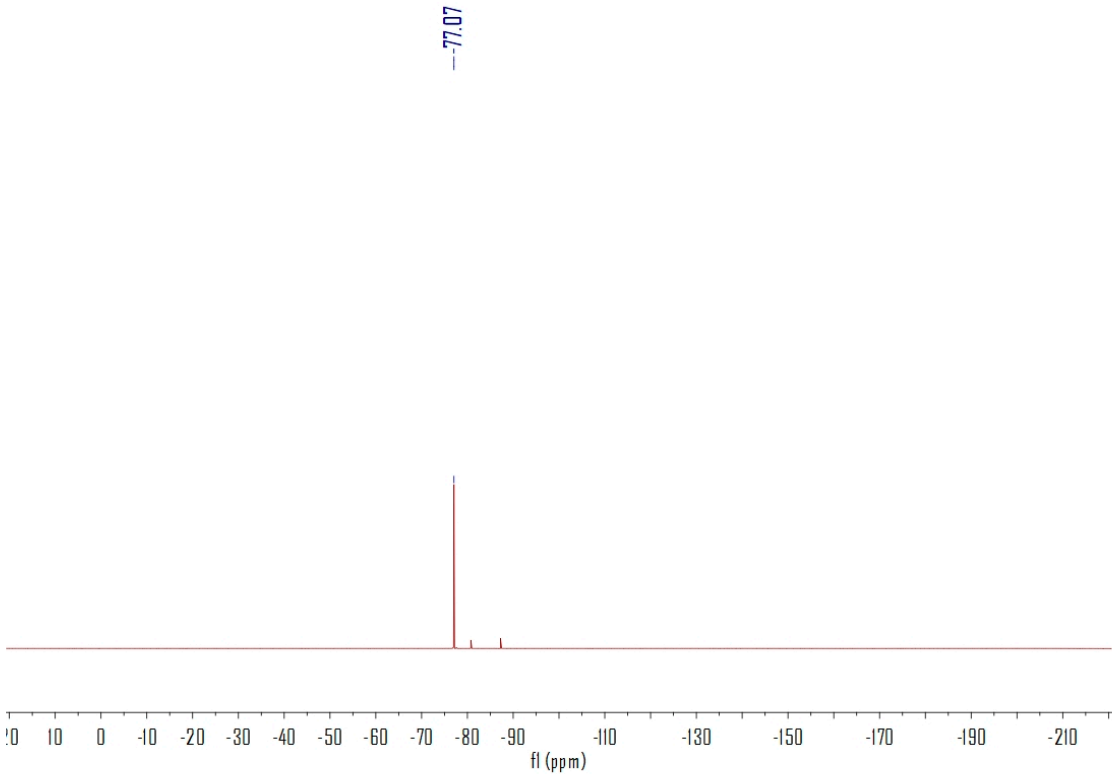

HRMS spectra

Figure S89. HRMS spectrum of the compound (**3aa**)

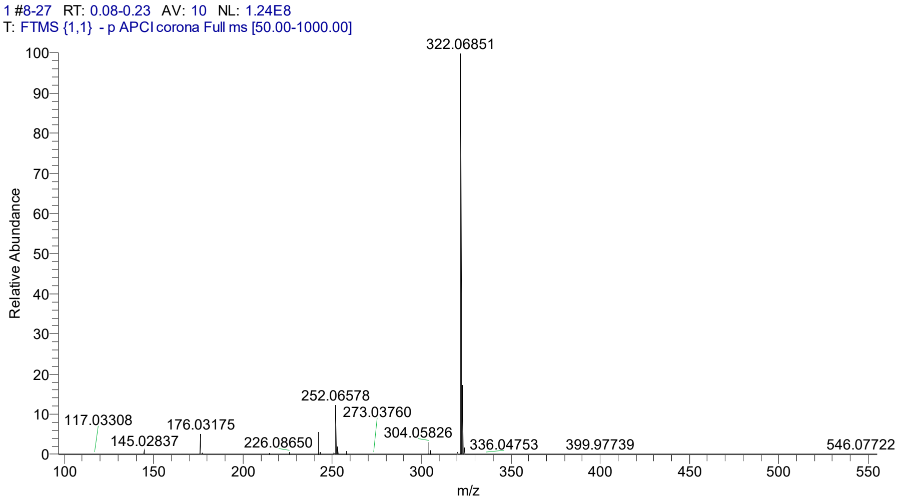

Figure S90. HRMS spectrum of the compound (**3ba**)

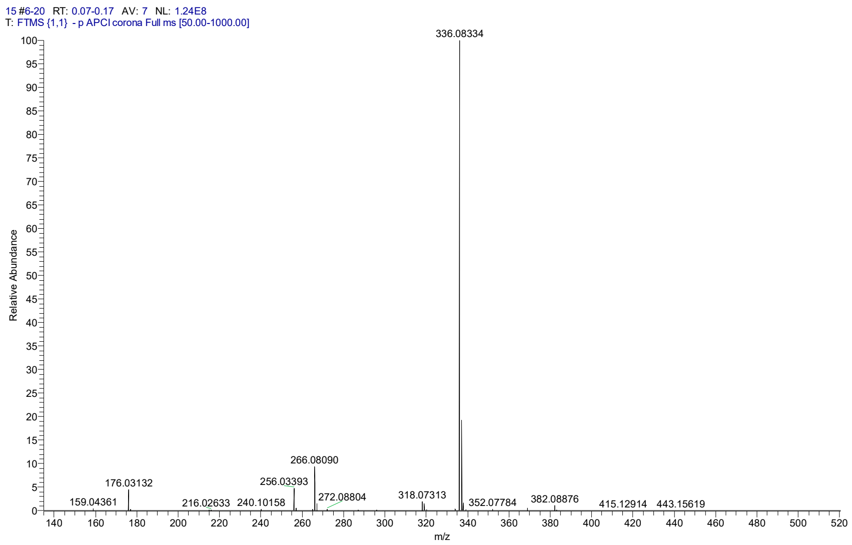

Figure S91. HRMS spectrum of the compound (**3ca**)

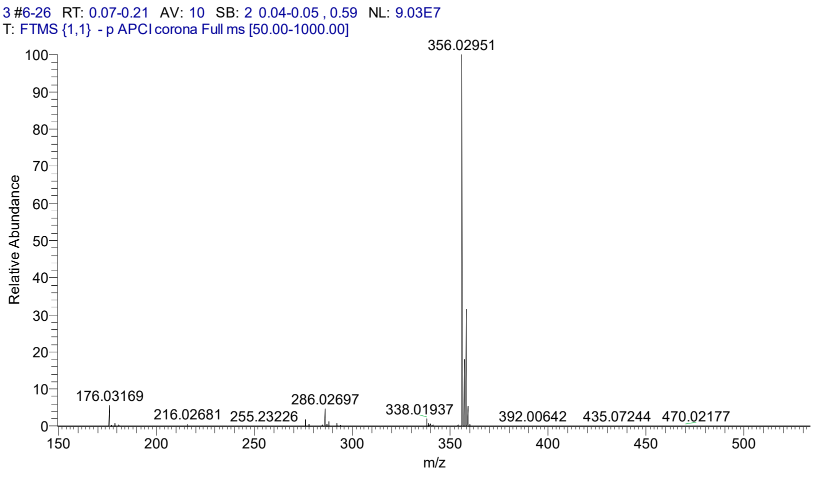

Figure S92. HRMS spectrum of the compound (**3da**)

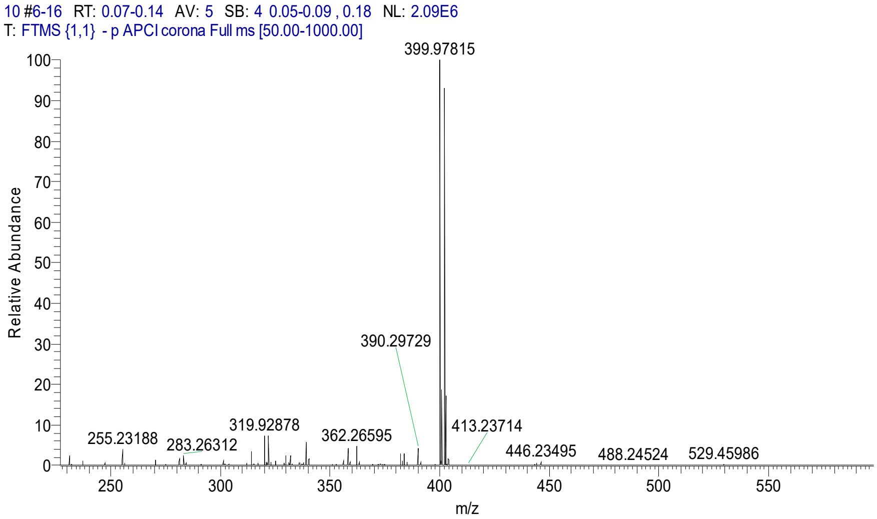

Figure S93. HRMS spectrum of the compound (**3ea**)

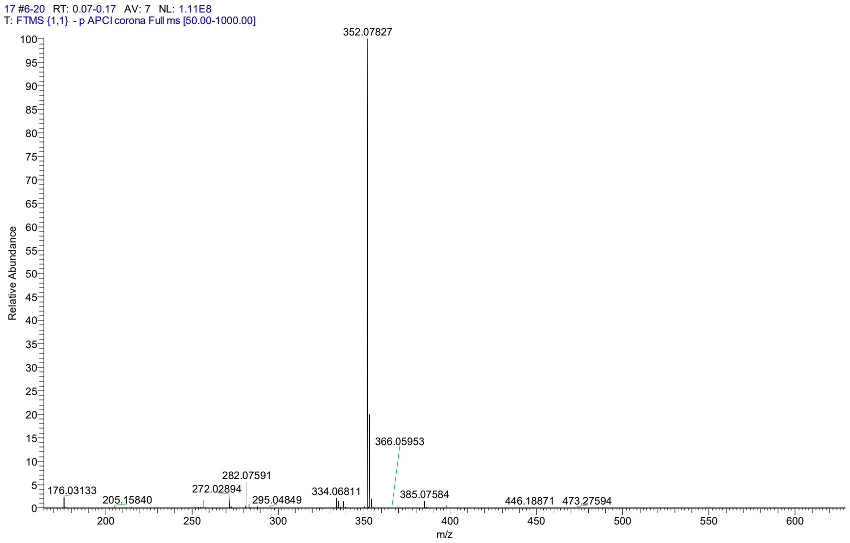

Figure S94. HRMS spectrum of the compound (**3fa**)

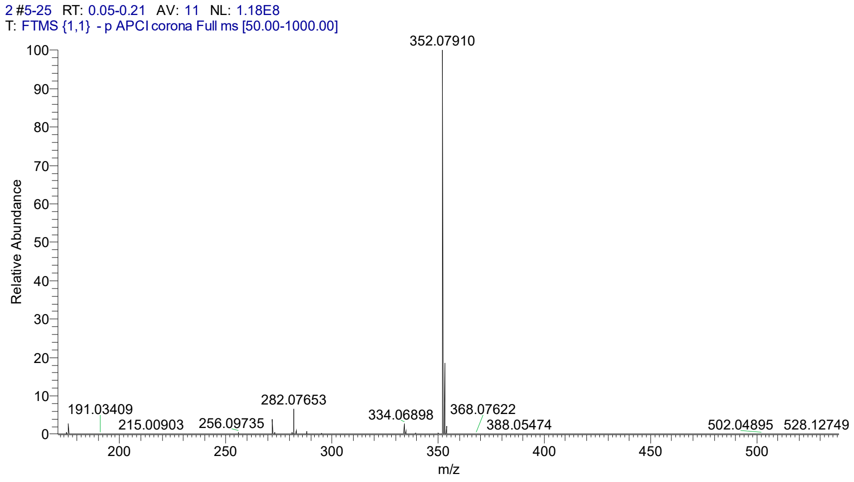

Figure S95. HRMS spectrum of the compound (**3ga**)

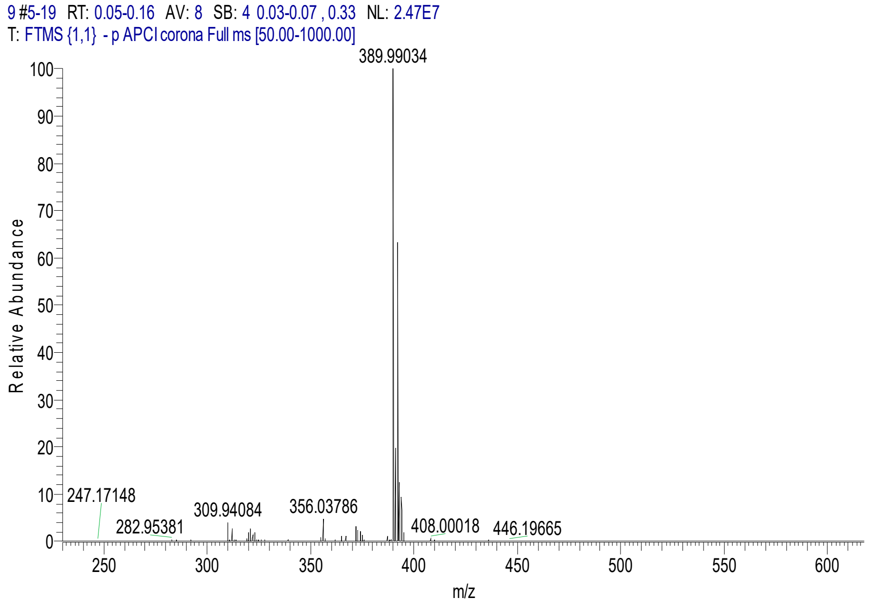

Figure S96. HRMS spectrum of the compound (**3ha**)

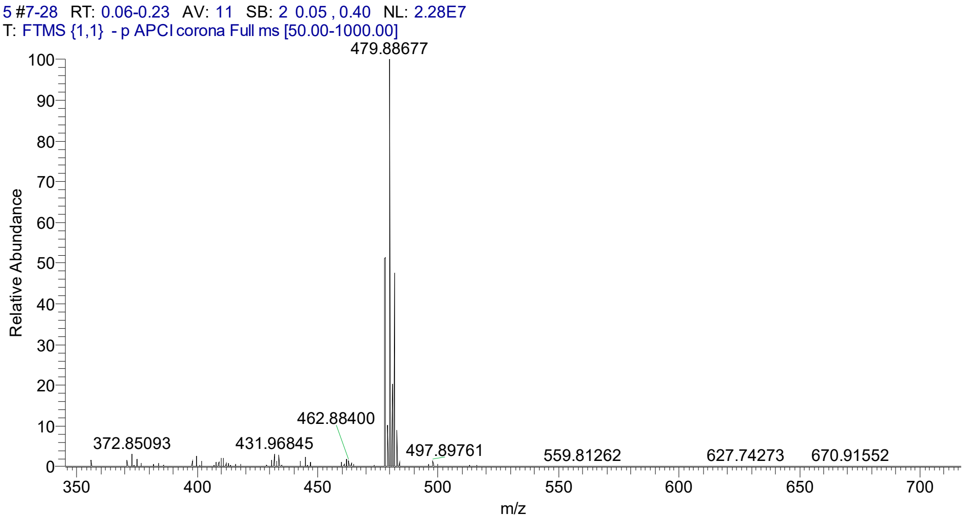

Figure S97. HRMS spectrum of the compound (**3ia**)

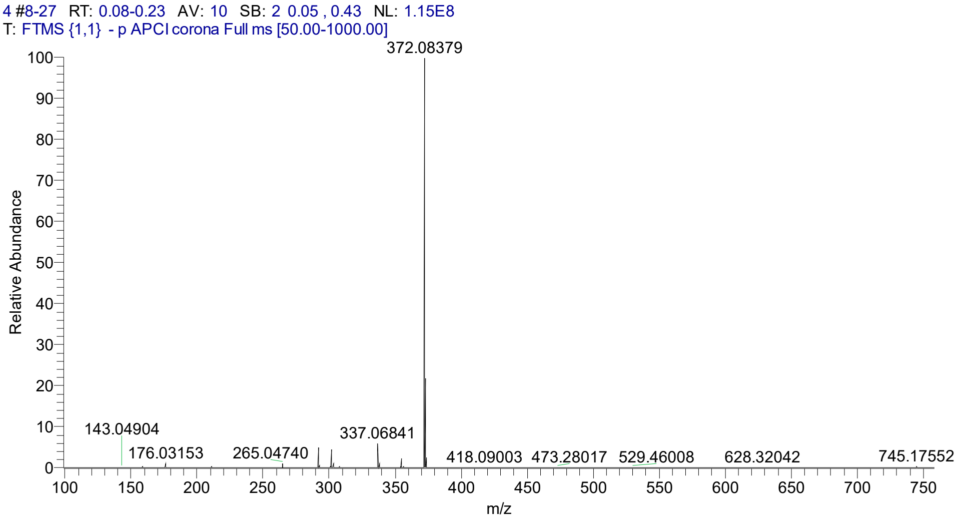

Figure S98. HRMS spectrum of the compound (**3ab**)

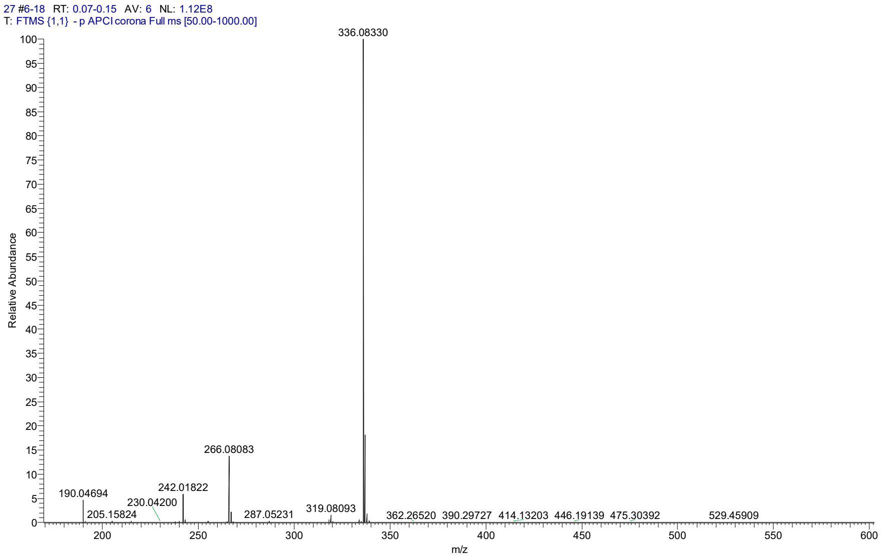

Figure S99. HRMS spectrum of the compound (**3fb**)

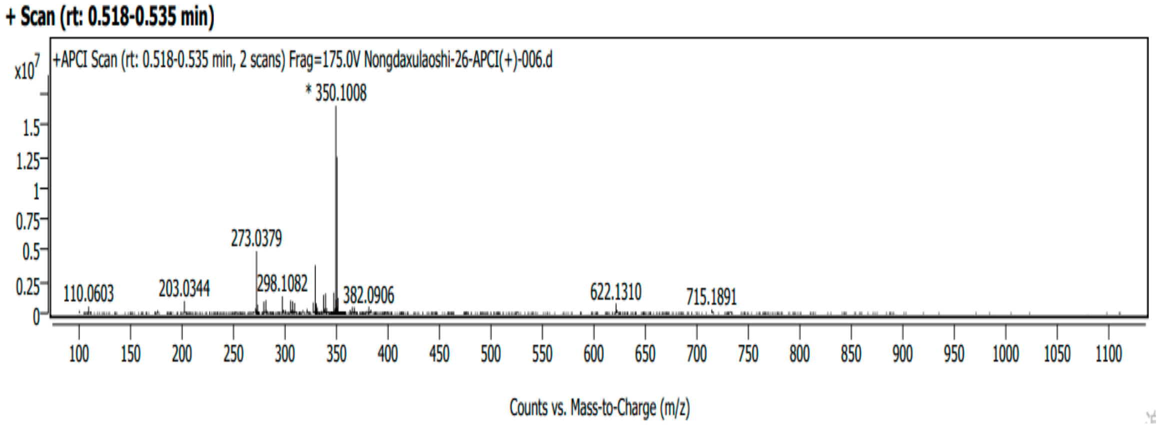

Figure S100. HRMS spectrum of the compound (**3ac**)

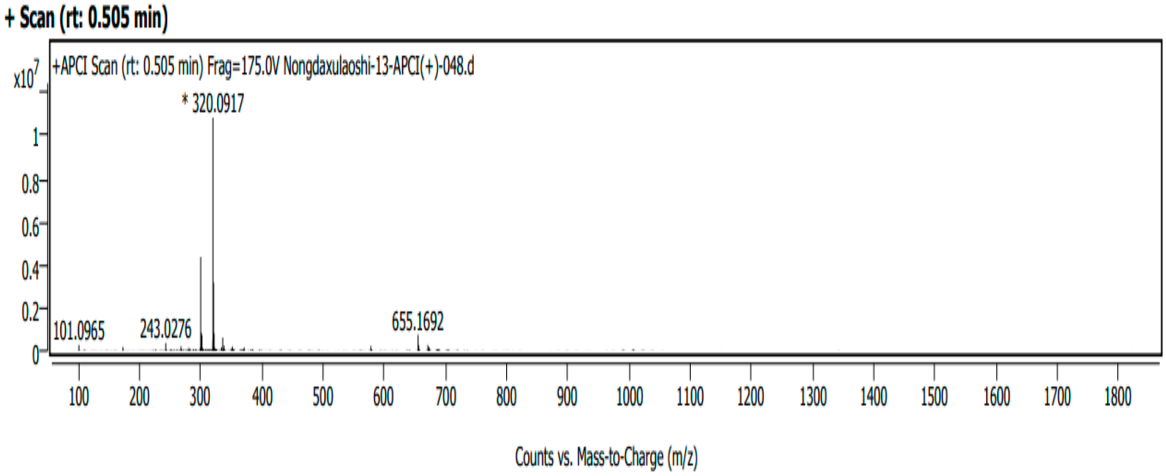

Figure S101. HRMS spectrum of the compound (**3bc**)

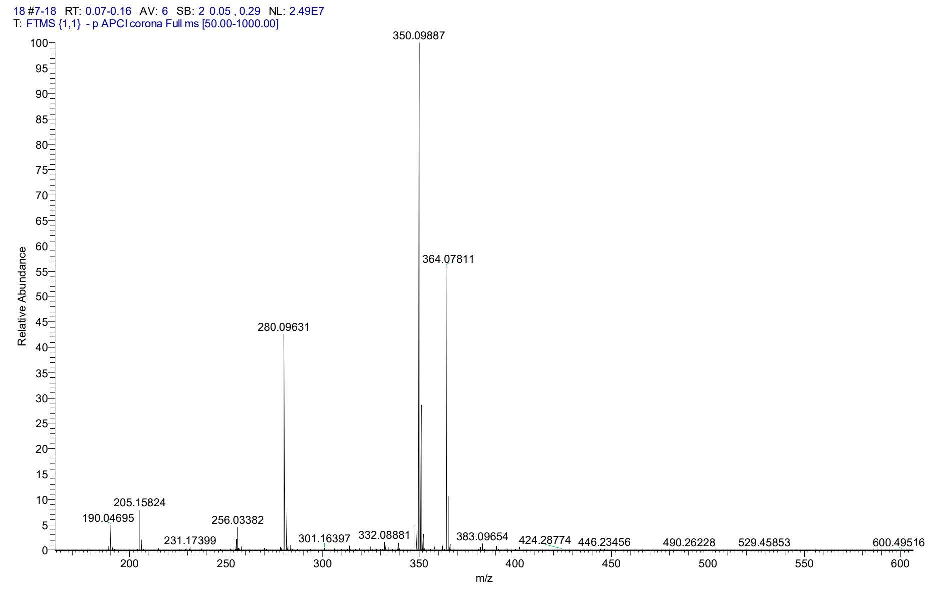

Figure S102. HRMS spectrum of the compound (**3cc**)

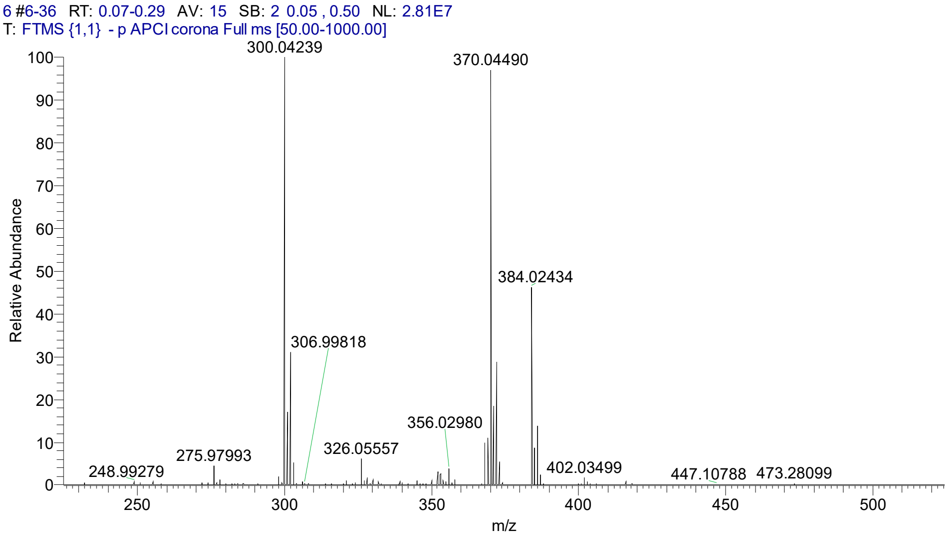

Figure S103. HRMS spectrum of the compound (**3dc**)

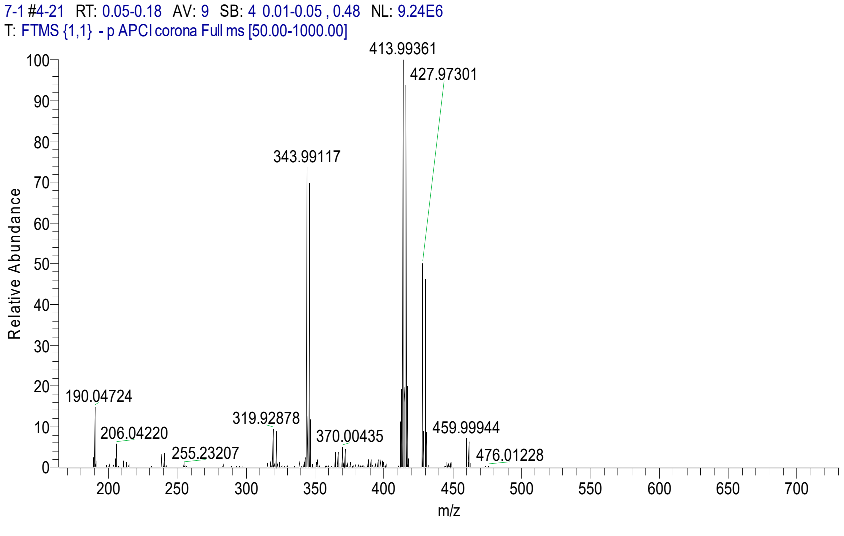

Figure S104. HRMS spectrum of the compound (**3ec**)

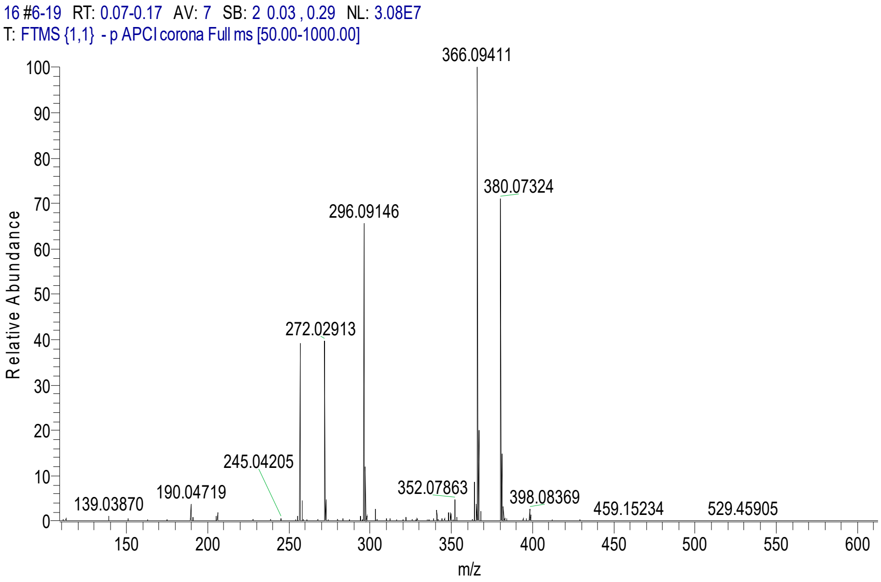

Figure S105. HRMS spectrum of the compound (**3fc**)

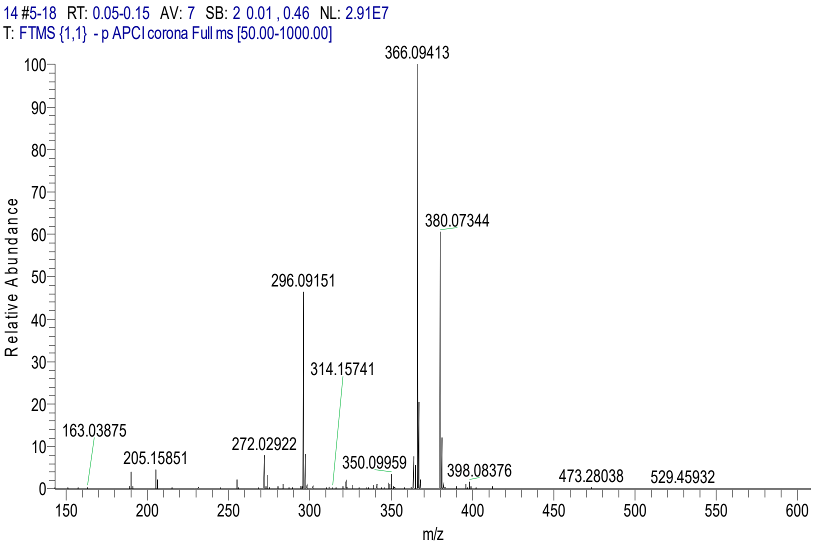

Figure S106. HRMS spectrum of the compound (**3gc**)

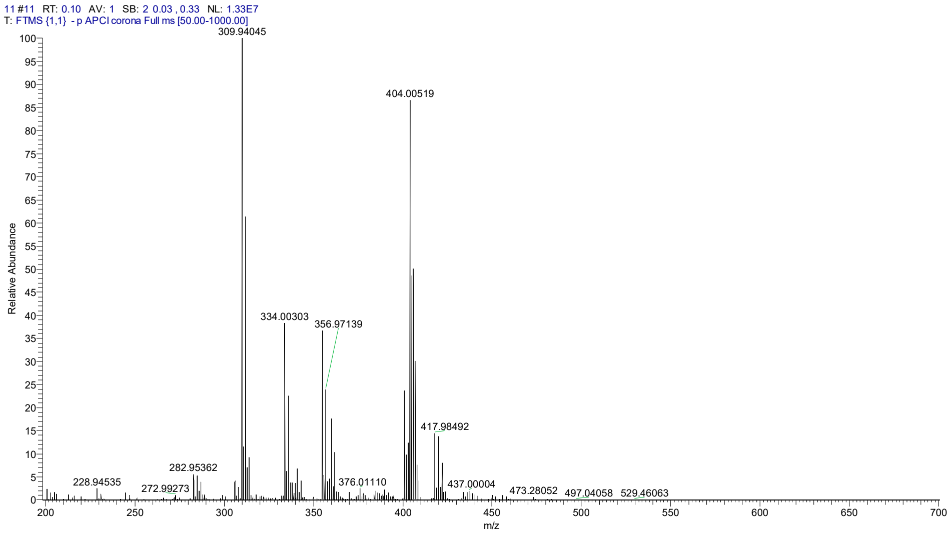

Figure S107. HRMS spectrum of the compound (**3hc**)

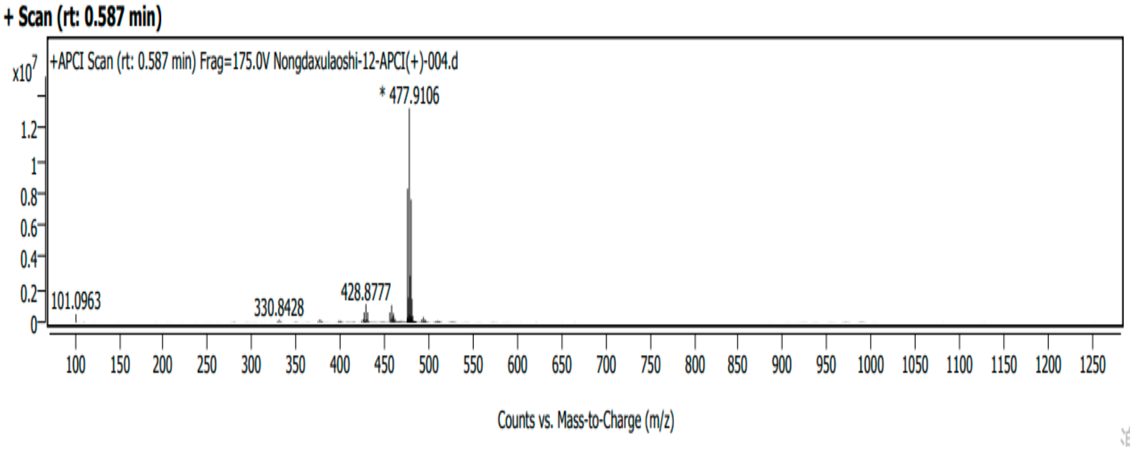

Figure S108. HRMS spectrum of the compound (**3ic**)

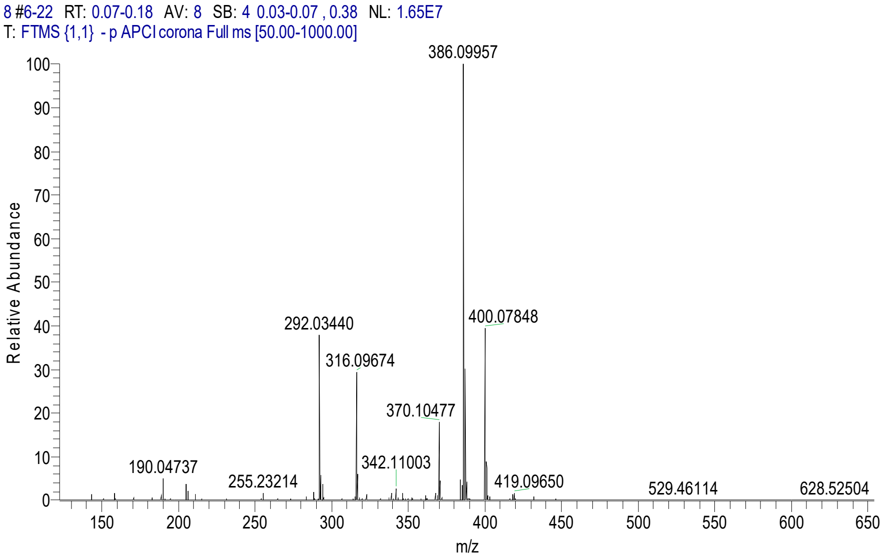

Figure S109. HRMS spectrum of the compound (**3ad**)

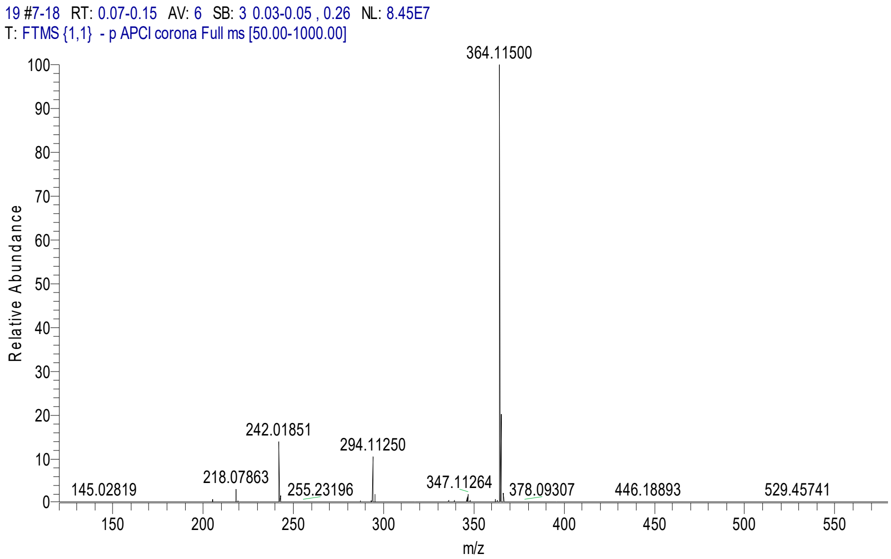

Figure S110. HRMS spectrum of the compound (**3bd**)

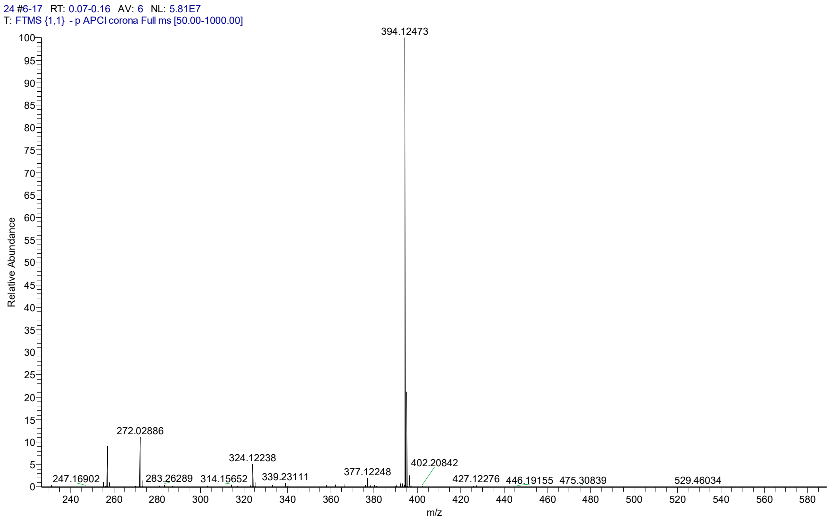

Figure S111. HRMS spectrum of the compound (**3cd**)

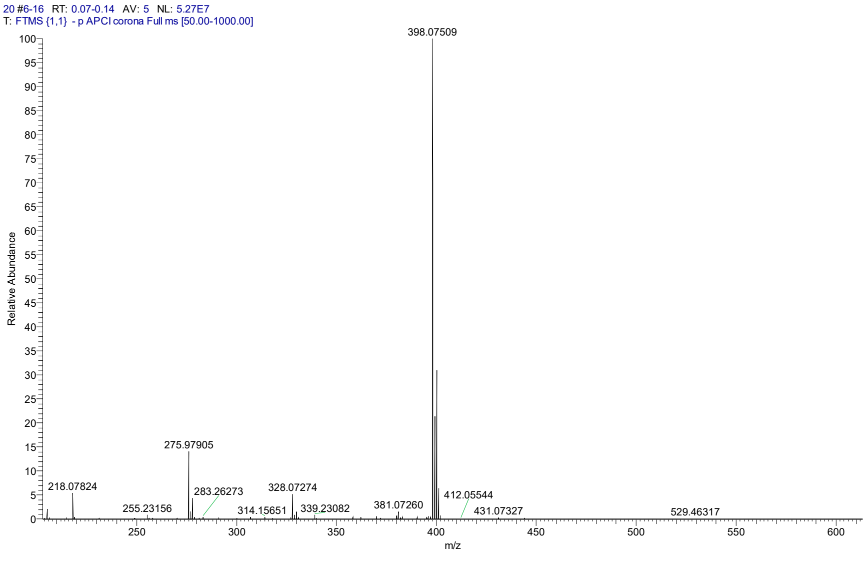

Figure S112. HRMS spectrum of the compound (**3dd**)

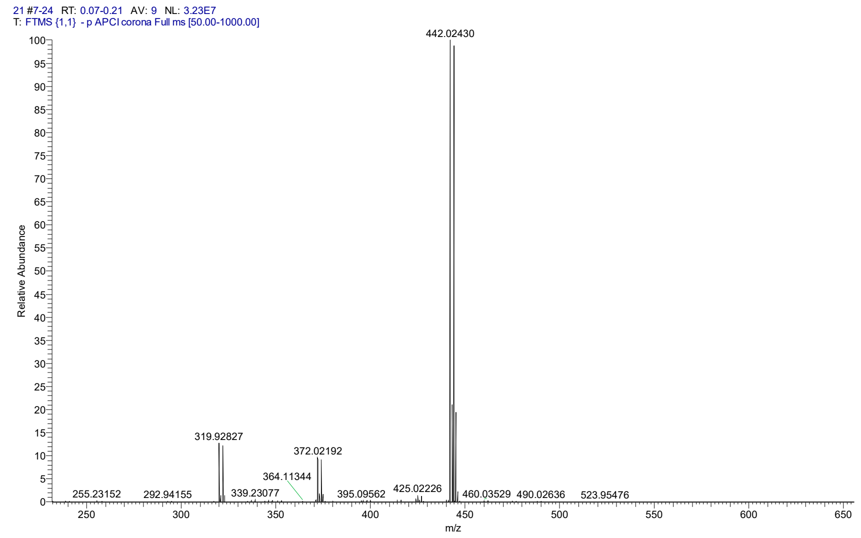

Figure S113. HRMS spectrum of the compound (**3ed**)

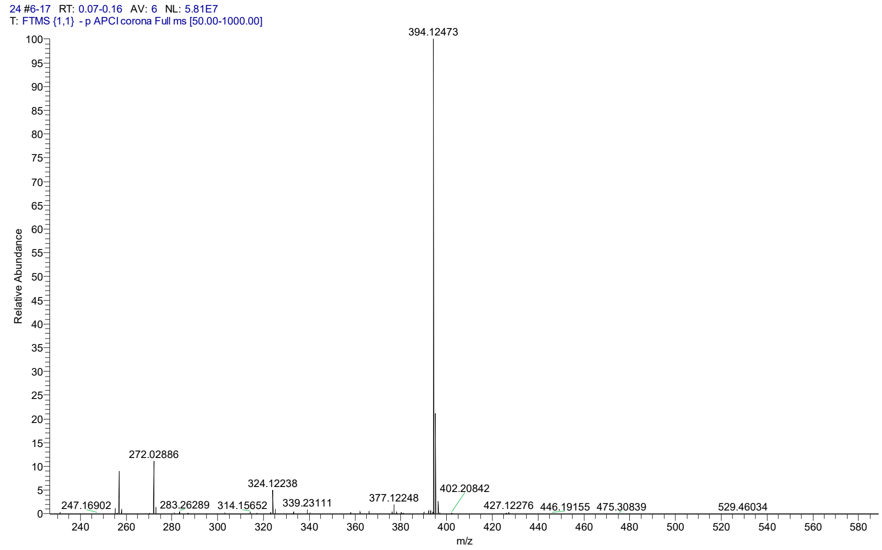

Figure S114. HRMS spectrum of the compound (**3gd**)

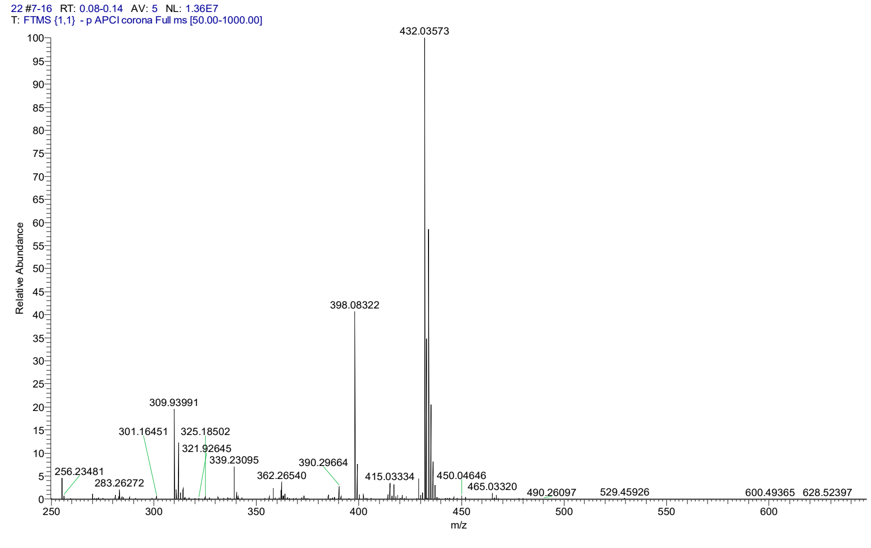

Figure S115. HRMS spectrum of the compound (**3hd**)

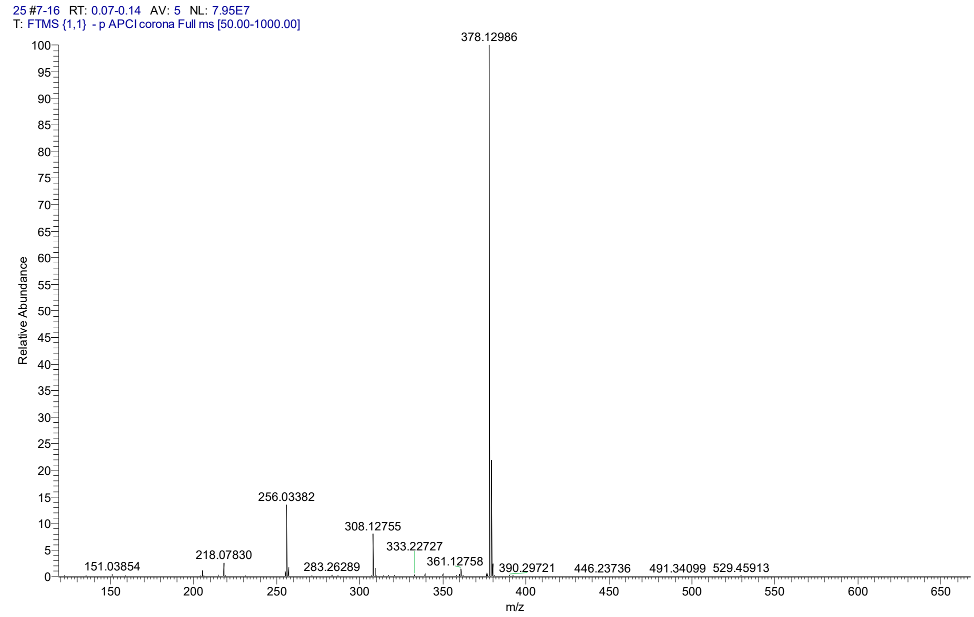

Figure S116. HRMS spectrum of the compound (**3ae**)

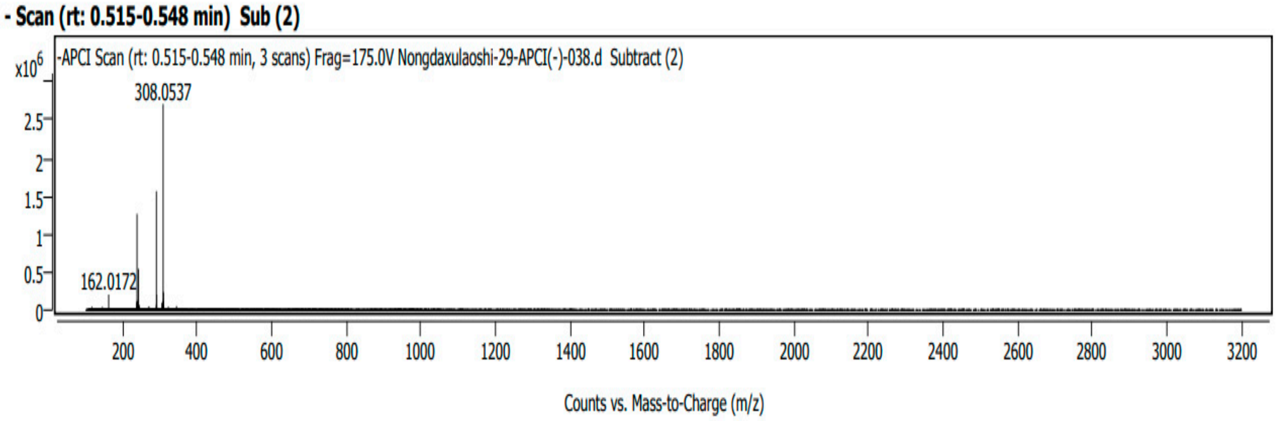

Figure S117. HRMS spectrum of the compound (**3af**)

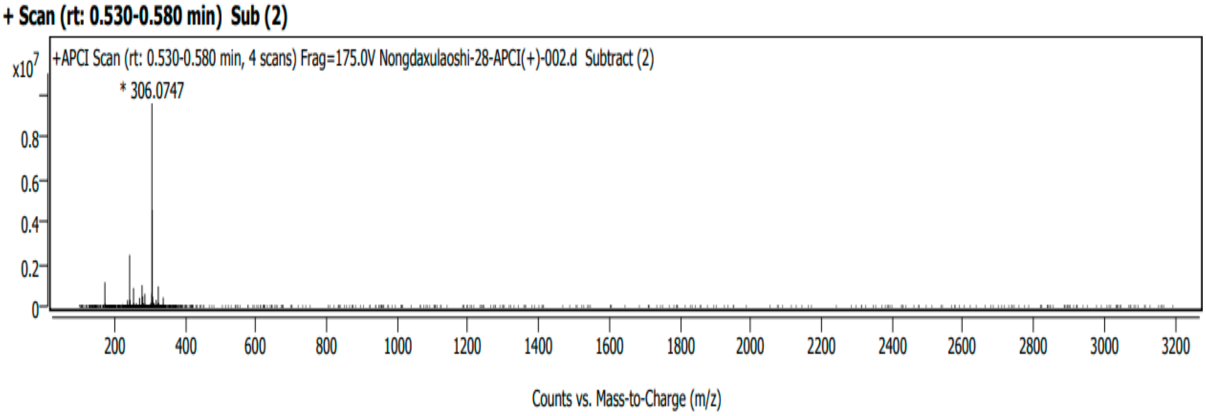

Supplement: Supplementary file 1 [file molecules-28-00260-s001.zip › molecules-2103455-Supplementary Information.pdf]
